# Asymmetric Catalytic Access to Piperazin-2-ones and Morpholin-2-ones in a One-Pot Approach: Rapid Synthesis of an Intermediate to Aprepitant

**DOI:** 10.1021/acs.joc.2c02491

**Published:** 2023-02-21

**Authors:** Sara Meninno, Alessandra Lattanzi

**Affiliations:** Dipartimento di Chimica e Biologia “A. Zambelli”, Università di Salerno, Via Giovanni Paolo II, 84084 Fisciano, Italy

## Abstract

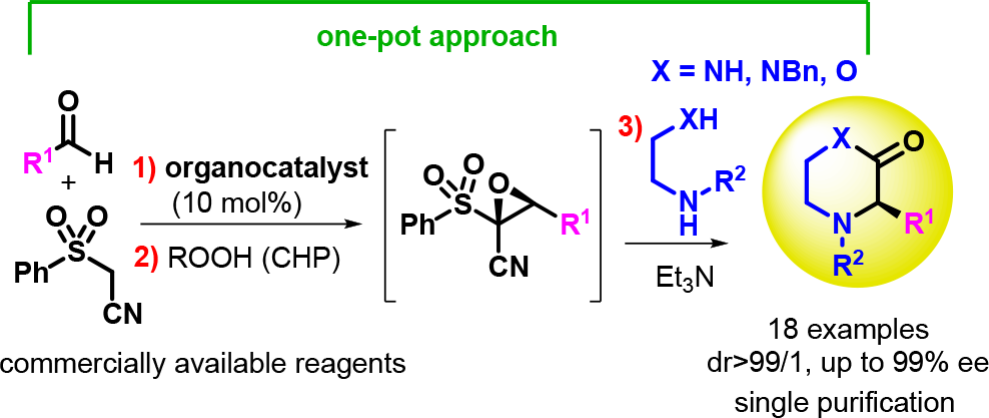

A one-pot Knoevenagel reaction/asymmetric epoxidation/domino ring-opening cyclization (DROC) has been developed from commercial aldehydes, (phenylsulfonyl)acetonitrile, cumyl hydroperoxide, 1,2-ethylendiamines, and 1,2-ethanol amines to provide 3-aryl/alkyl piperazin-2-ones and morpholin-2-ones in yields of 38 to 90% and up to 99% ee. Two out of the three steps are stereoselectively catalyzed by a quinine derived urea. The sequence has been applied for a short enantioselective entry to a key intermediate, in both absolute configurations, involved in the synthesis of the potent antiemetic drug Aprepitant.

The past decade experienced an increasing growth of one-pot asymmetric cascade processes applied to the synthesis of biologically active targets and drugs.^1^ The development of these protocols is highly appealing, especially in view of potential industrial applications, where purification of the intermediates for each step is avoided and consequently economic and time costs as well as the environmental impact are conveniently minimized.^2^ This approach appears particularly suited for the synthesis of heterocyclic compounds, which are abundant structural cores in natural products and pharmaceuticals.^3^ Stereocontrolled preparations of heterocyclic drugs or key intermediates to access natural products have been elegantly carried out under one-pot operations, as exemplified by (−)-oseltamivir phosphate (Tamiflu),^4^ a bicyclic intermediate to prostaglandins,^5^*Daphniphyllum* alkaloids,^6^ or amino alcohols.^7^

Among the chiral nonracemic medium sized heterocyclic scaffolds, piperazin-2-ones^8^ and morpholines/morpholin-2-ones^9^ are endowed with several important bioactivies and are building blocks for peptide synthesis as illustrated for (−)-Praziquantel a potent antihelminthic drug,^10^ serine protease inhibitor Pseudotheonamide A1,^11^ and Aprepitant, approved by the FDA to prevent nausea and vomiting in cancer drug therapy (Figure 1).^12^

**Figure 1:**
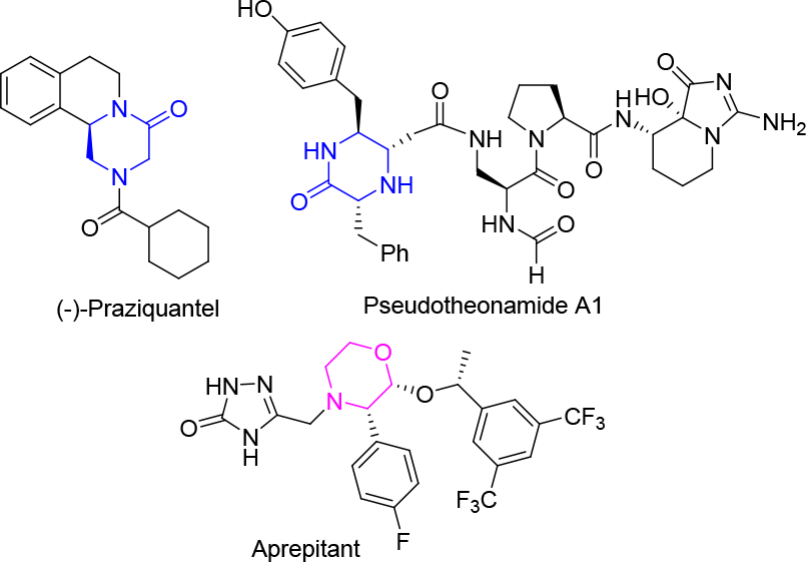
Optically active piperazin-2-one and morpholine/morpholin-2-one containing drugs and bioactive compounds.

Although chiral pool and auxiliary based procedures have been developed,^13^ the asymmetric catalytic methodologies reported so far to prepare piperazin-2-ones are limited and rare for C3-substituted morpholin-2-ones (Scheme 1). Ir-^14^ or Pd-^15^ catalyzed hydrogenation of unsaturated piperazin-2-ones represents a versatile strategy to access piperazin-2-ones, bearing stereogenic centers at different positions of the heterocycle in a good to high level of steroselectivity (Scheme 1(a)).

**Scheme 1:**
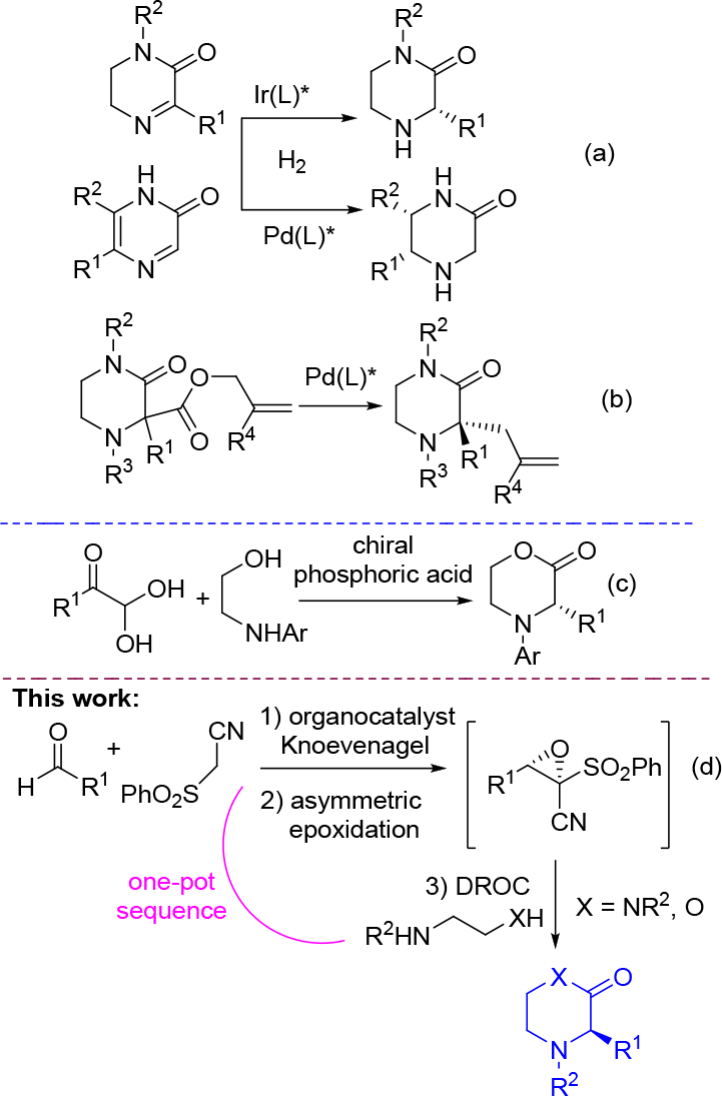
Strategies for the Asymmetric Catalytic Synthesis of Piperazin-2-ones and Morpholin-2-ones

Interestingly, the Pd-catalyzed decarboxylative allylic alkylation of racemic piperazin-2-ones enables obtaining piperazin-2-ones bearing a C3-quaternary stereocenter in high enantioselectivity (Scheme 1(b)).^16^ A few miscellaneous asymmetric approaches to piperazin-2-ones were also reported under different catalytic conditions.^17^

Concerning the synthesis of C3-substituted morpholin-2-ones, chiral auxiliary tools have been exploited using enantiopure 1,2-amino alcohols and aryl glyoxals in a Brønsted acid catalyzed condensation and rearrangement to morpholinones.^18^

Only recently, Zhu and coauthors illustrated the first catalytic process using the chiral phosphoric acid promoted reaction of aryl/alkylglyoxals and 2-(arylamino)ethan-1-ols.^19^ The domino process consists of [4 + 2] heteroannulation followed by a 1,2-aryl/alkyl shift of the resulting cyclic α-iminium hemiacetal to give the C3-substituted morpholin-2-ones in good to high enantioselectivity (Scheme 1(c)).

Chiral nonracemic epoxides are among the most effective intermediates applied in the asymmetric synthesis of bioactive compounds, including heterocycles.^20^ Often, in these procedures, the epoxide intermediates are isolated and then subjected to the following ring-opening step in another reaction vessel, as usually done in a stop and go synthesis.

In view of the great advantages of carrying out a one-pot process, we recently focused our efforts on the enantioselective preparation of dihydroquinoxalinones, a scaffold of interest in medicinal chemistry.^21^ Key to the success of the process was the identification of 1-phenylsulfonyl-1-cyano epoxides as new masked α-halo acyl halide synthons, involved in an organocatalytic one-pot sequence, performed in a single solvent, using commercially available reagents.

On the grounds of these results, we envisaged the possibility to develop the first general catalytic and one-pot asymmetric synthesis of C3-substituted piperazin-2-ones and morpholin-2-ones via a sequential quinine-derived urea (eQNU) catalyzed Knoevenagel reaction/asymmetric epoxidation followed by a domino ring-opening cyclization (DROC)^22^ with 1,2-ethylendiamines and 2-benzylamino ethanol (Scheme 1(d)).

We commenced the study working under previously optimized conditions,^21^ using commercially available aromatic aldehydes and phenylsulfonyl(acetonitrile) for the first step in the presence of 10 mol % of **eQNU**. Cumyl hydroperoxide (CHP) was successively added for the asymmetric epoxidation of the in situ formed electron-poor *E*-alkenes, followed by the final addition of *N*,*N*′-dibenzylethylendiamine or ethylendiamine necessary for the DROC process (Table 1).

**Table 1:**
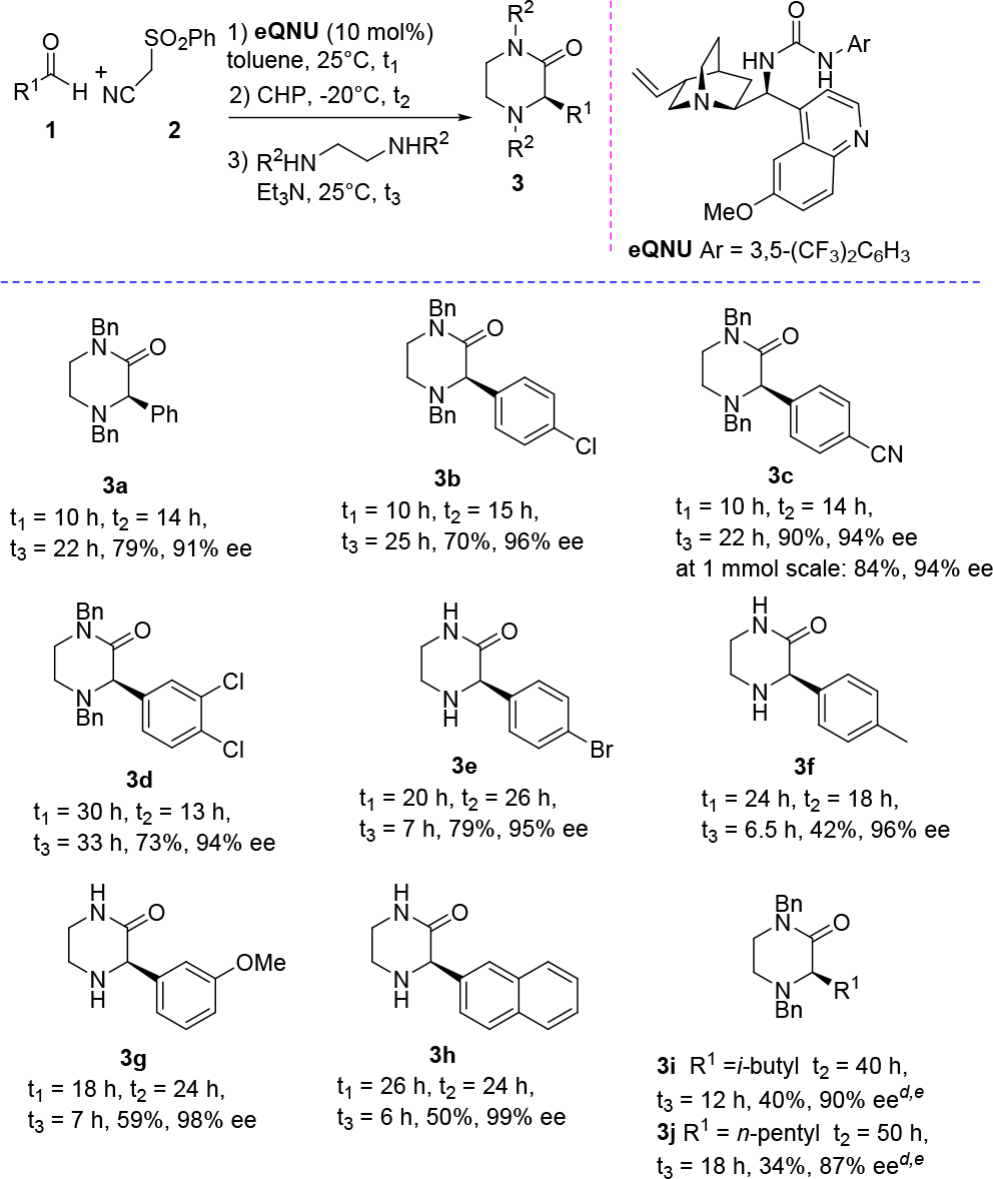
One-Pot Enantioselective Organocatalytic Synthesis of Piperazin-2-ones^a^^–^c

a Reaction conditions: a) Knoevenagel step: (phenylsulfonyl)acetonitrile (0.1 mmol), aldehyde (0.1 mmol), and eQNU (0.01 mmol) in anhydrous toluene (*C* = 0.3 M). b) Epoxidation step: dilution of the reaction with toluene (*C* = 0.02 M) and lowering at −20 °C, then adding CHP (0.11 mmol). DROC step: c) addition of 1,2-ethylendiamine (0.12 mmol) and Et_3_N (0.2 mmol) at 25 °C.

b Yield of isolated product after chromatography.

c HPLC analysis on a chiral stationary phase.

d The process was performed starting from the alkene.

e The DROC step was carried out at 50 °C.

Pleasingly, the formation of the bis *N*-protected (*R*)-piperazin-2-ones **3a**–**d**, bearing halogen atoms or a cyano group substituted phenyl ring smoothly proceeded in high overall yield and ee values (up to 96% ee). The reaction proceeded with a comparable outcome for compound **3c** when scaling up the corresponding aldehyde to a 1 mmol amount.

Similar results were achieved when aromatic aldehydes bearing a halogen atom, methoxy and methyl groups at *para*- or *meta*-positions, and naphthylaldehyde were used together with ethylendiamine in the DROC step. The corresponding NH-free heterocycles **3e**–**h** were obtained in good yield and excellent enantioselectivity (up to 99% ee).

The installation of an alkyl group at the stereogenic center has been shortly assessed, using *N*,*N*′-dibenzylethylendiamine and starting the one-pot sequence from the corresponding electron-poor alkene, given the challenges experienced when performing the Knoevenagel reaction directly from aliphatic aldehydes and (phenylsulfonyl)acetonitrile.^21^ Piperazin-2-ones **3i**,**j**, bearing branched or linear alkyl groups, were obtained in acceptable overall yields with the ee values maintained, attesting the versatiliy of this methodology for the asymmetric synthesis of C3-aryl/alkyl substituted piperazin-2-ones.

We next investigated the applicability of the one-pot process to prepare C3-substituted morpholin-2-ones, working under the same conditions but adding in the final step 2-benzylamino ethanol (Table 2).

**Table 2:**
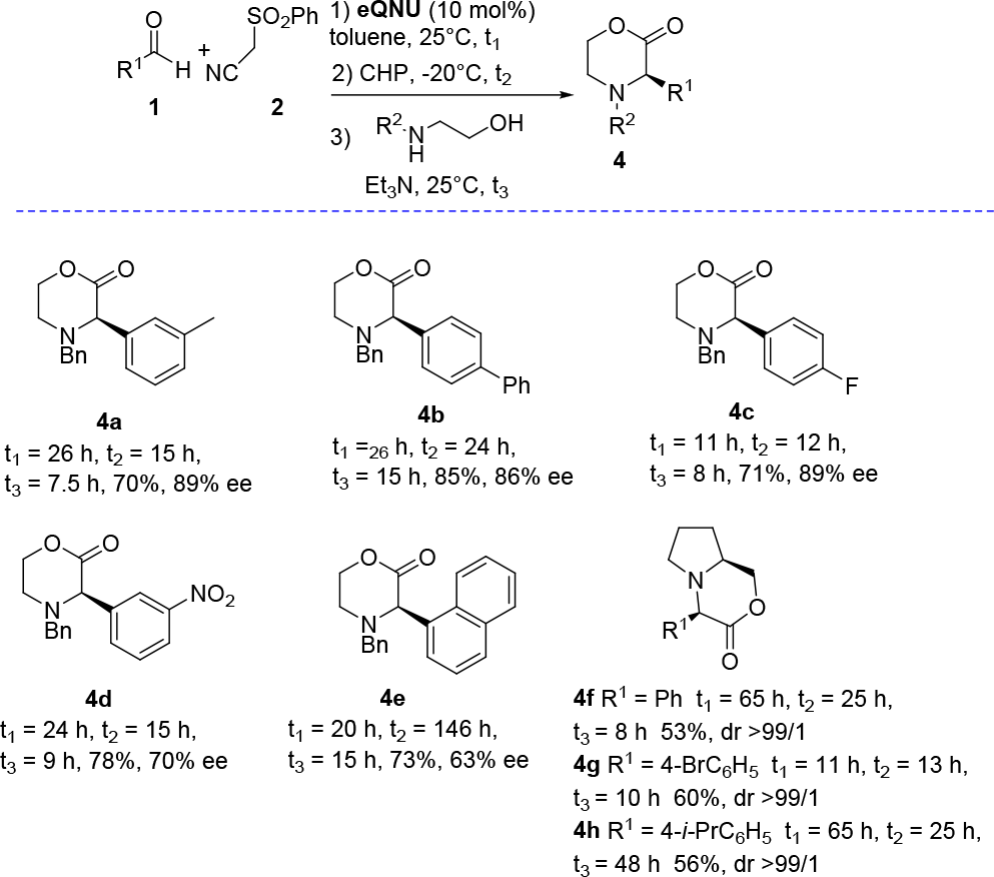
One-Pot Stereoselective Organocatalytic Synthesis of Morpholin-2-ones^a^^–^c

a Reaction conditions as reported in Table 1.

b Yield of the isolated product after chromatography.

c HPLC analysis on a chiral stationary phase.

Pleasingly, (*R*)-morpholin-2-ones **4a**–**d**, bearing the C3-phenyl group substituted with alkyl, phenyl, fluorine atom, and nitro groups at *meta*- or *para*- positions, were recovered in high yield and fairly good enantioselectivity (up to 89% ee). Morpholin-2-one **4d** showed a lower ee value (70% ee), likely ascribed to partial racemization due to increased acidity of the proton at the stereocenter, dictated by the presence of the nitro group.^23^ When more sterically demanding 1-naphthyl aldehyde was used as the starting reagent, the corresponding morpholin-2-one **4e** was isolated in 73% yield and 63% ee.

Next, optically pure l-prolinol was introduced in the DROC step, with a view to provide the first highly enantio- and diastereoselective preparation of bicyclic morpholin-2-ones. Mlostoń and co-workers recently illustrated an alternative one-pot approach to this class of morpholin-2-ones reacting l-prolinol with arylglyoxal to give 2-aroyl-1,3-oxazolidines. The latter underwent an acid catalyzed cascade ring-opening reaction, followed by a 1,2-aryl shift and lactonization to fused morpholin-2-ones **4**.^24^ The cascade process yielded products **4** with moderate to high dr ratios, according to the substitution pattern of the aromatic ring installed at the stereocenter. Our protocol delivered the *endo*-oriented aryl group heterocycles **4f**–**h** as the only diastereoisomer, in optically pure form. The enantioselective epoxidation followed by the epoxide ring-opening reaction secured a highly stereocontrolled pathway to bicyclic morpholin-2-ones **4f**–**h**, which were isolated in good overall yield.

3-Aryl substituted morfolin-2-ones **4** are scaffolds of interest in the pharmaceutical industry and useful building blocks to construct the privileged morpholine core. In 1998^12^ and 2002,^25^ chemists at Merck reported synthetic protocols to the drug Aprepitant, marketed as EMEND, involving either optically enriched *S*- or *R*-**4c** as the key intermediate. The morpholin-2-one **4c** was synthesized using l-α-amino acids as reagents from the chiral pool.^26^

In Table 2, we have reported the shortest and first one-pot catalytic preparation of intermediate (*R*)-**4c** in 71% yield and 89% ee. Being the final drug (*S*)-configured at the stereogenic center of interest, we spent our efforts to access optically enriched (*S*)-**4c**. To achieve this goal, the asymmetric epoxidation of the corresponding alkene **5**, using pseudoenantiomeric eQD-based organocatalysts, was preliminarily investigated (Table 3).

**Table 3:**
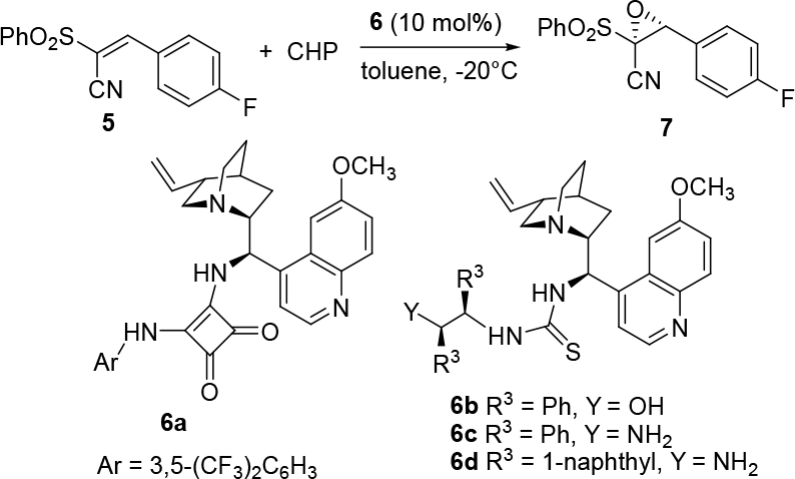
Asymmetric Epoxidation of Alkene **5**^a^

entry	**6**	*t* (h)	yield (%)^b^	ee (%)^c^
1	**6a**	20	49	21
2	**6b**	15	35	10
3	**6c**	16	97	58
4	**6d**	15	80	76
5^d^	**6d**	48	98	72
6^e^	**6d**	35	57	86
7^f^	**6d**	24	50^g^	78

a Reaction conditions: alkene **5** (0.1 mmol), **6** (0.01 mmol), and CHP (0.11 mmol) in anhydrous toluene (5 mL).

b Yield determined by ^1^H NMR analysis of the crude reaction mixture comparing signals of the product with the residual alkene.

c HPLC analysis on a chiral stationary phase.

d Reaction carried out with 5 mol % of **6d**.

e Reaction carried out at −50 °C.

f One-pot reaction starting from *p*-F-benzaldehyde and (phenylsulfonyl)acetonitrile as reported in Table 2, with the Knoevenagel step carried out for 22 h.

g The yield of isolated epoxide is underestimated, due to an unmeasured residual fraction of epoxide coeluted with cumyl alcohol.

The eQD-derived squaramide **6a** afforded, under usual conditions, the expected (*R*,*R*)-epoxide **7** in moderate yield and low ee value (entry 1). This result is in agreement with previous observations attesting eQD-based thioureas to be less efficient in terms of enantiocontrol than eQN-based analogs.^21^ Hence, we thought to employ multifunctionalized organocatalysts based on the eQD-skeleton, bearing additional chiral portions and H-bonding groups, to modulate the activity and stereocontrol.^27^

The epoxidation using catalyst **6b** led to poor conversion and enantioselectivity (entry 2), whereas the introduction of a diamine core in catalyst **6c** was found to be beneficial, providing high conversion to the epoxide which showed 58% ee (entry 3). The importance of an additional H-bonding group in the chiral framework is evident when comparing data in entries 2 and 3, with the NH_2_ group being more effective than the OH group. We were delighted to observe a further improvement up to 76% ee, when using catalyst **6d,** bearing a more sterically demanding 1-naphthyl substituted diamine fragment (entry 4). Besides the sterics of the diamine residue, the primary NH_2_ group in catalysts **6c**,**d** is likely to be involved in additional H-bonding interactions with the phenyl sulfonyl group of the alkene favorably affecting the stereocontrol.^28^

The reaction catalyzed by 5 mol % of catalyst **6d** proceeded to completion after a longer reaction time, and a slightly decreased ee value was observed (entry 5). Working at −50 °C with 10 mol % of **6d** slowed down the process, but the ee value was raised to 86% (entry 6).

The one-pot process was then investigated starting from *p*-fluorobenzaldehyde and phenylsulfonyl(acetonitrile) to form alkene **5**, and then the epoxidation was carried out at −20 °C (entry 7). The epoxide was isolated in 50% yield and 78% ee. No loss of stereoselectivity has been observed when comparing this outcome with that in entry 4, indicating the suitability of **6d** to catalyze the Knoevenagel condensation.

Next, the best reaction conditions observed in the asymmetric epoxidation of alkene **5** (entry 6, Table 3) were used to prepare (*S*)-**4c** in a one-pot synthesis (Scheme 2(a)).

**Scheme 2:**
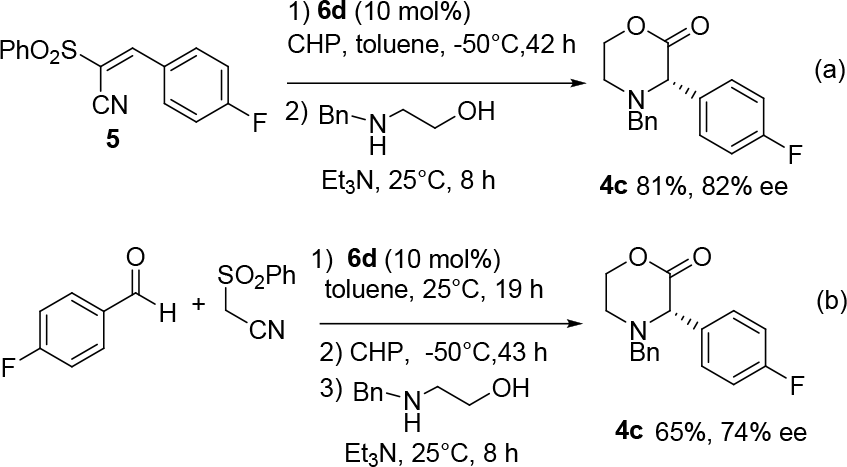
One-Pot Enantioselective Syntheses of (*S*)-Morpholin-2-one **4c**^*a*^

Starting from alkene **5**, the heterocycle was successfully isolated in 81% yield and 82% ee, a result which favorably competes with a previously reported catalytic stop and go sequence to produce (*S*)-**4c**.^29^ Finally, the three-step sequential protocol, performed from the aldehyde, was investigated (Scheme 2(b)). In this case, the heterocycle **4c** was isolated in good yield and 74% ee.

In conclusion, a first telescoped catalytic synthesis of 3-aryl/alkyl piperazin-2-ones and 3-aryl morpholin-2-ones was successfully accomplished using commercially available reagents and readily available urea and thiourea catalysts based on Cinchona alkaloids. The straightforward protocol enables the preparation of these classes of useful heterocycles in good to high yield and enantioselectivity. The versatility of the process has been confirmed by a rapid access to both enantioenriched morpholin-2-ones, key intermediates for the synthesis of the drug Aprepitant. Further applications of the one-pot strategy, involving 1-phenylsulfonyl-1-cyano epoxide intermediates in asymmetric synthesis, will be the subject of forthcoming reports.

## Data Availability

The data underlying this study are available in the published article and its Supporting Information.

## Supporting Information Available

General experimental procedures, characterization data, copies of NMR spectra, and HPLC traces for compounds **3**, **4**, and **7** (PDF)
FAIR data, including the primary NMR FID files, for compounds **3a**–**j**, **4a**–**h**, **7** (ZIP)
