# Supplementary material for: Asymmetric Catalytic Access to Piperazin-2-ones and Morpholin-2-ones in a One-Pot Approach: Rapid Synthesis of an Intermediate to Aprepitant
Source: J Org Chem. 2023 Feb 21;88(12):7888–92. doi: 10.1021/acs.joc.2c02491 (PMC10278953; doi:10.1021/acs.joc.2c02491)
Supplement: Supplementary file 1 — jo2c02491_si_001.pdf [file jo2c02491_si_001.pdf]

# Asymmetric Catalytic Access to Piperazin-2-ones and Morpholin-2-ones in a One-Pot Approach: Rapid Synthesis of an Intermediate to Aprepitant

Sara Meninno, and Alessandra Lattanzi*

[a] *Dipartimento di Chimica e Biologia "A. Zambelli", Università di Salerno, Via Giovanni Paolo II, I-84084, Fisciano, Italy*

*Corresponding author email: lattanzi@unisa.it*

**General Methods** ........ **S2**

**Experimental Procedures and Compounds Characterization** ........ **S3**

**General procedure for the racemic synthesis of heterocycles 3a-3j, 4a-4h** ........ **S3**

**General procedure for one-pot asymmetric Knoevenagel/epoxidation/ring-opening reaction to heterocycles 3a-3j, 4a-4h** ........ **S3**

**Scale-up asymmetric synthesis of (*R*)-4-(1,4-dibenzyl-3-oxopiperazin-2-yl)benzonitrile (3c)** ........ **S4**

**NMR spectra** ........ **S14**

**HPLC chromatograms** ........ **S52**

## General Methods

All reactions requiring dry or inert conditions were conducted in flame-dried glassware under a positive pressure of nitrogen. Anhydrous toluene was purchased from Aldrich and used as received, all other solvents were dried over molecular sieves. Molecular sieves (Aldrich Molecular Sieves, 3 Å, 1.6 mm pellets) were activated under vacuum at 200 °C overnight. Reactions were monitored by thin layer chromatography (TLC) on Macherey-Nagel pre-coated silica gel plates (0.25 mm) and visualized by UV light and phosphomolybdic acid or ninhydrin stains. Flash chromatography was performed on Merck silica gel (60, particle size: 0.040–0.063 mm). $^{1}$H NMR and $^{13}$C NMR spectra were recorded on Bruker Avance III HD 600, Bruker Avance-400, Bruker Avance-300 spectrometer in $CDCl_3$. Chemical shifts for protons are reported using residual solvent protons ($\delta$ = 7.26 ppm for $CDCl_3$) as internal standard. Carbon spectra were referenced to the shift of the $^{13}$C signal of $CDCl_3$ ($\delta$ = 77.0 ppm).

The following abbreviations are used to indicate the multiplicity in NMR spectra: s - singlet; d - doublet; t - triplet; q - quartet; dd – double doublet; ddd – doublet of doublet of doublets; dt – doublet of triplets; td – triplet of doublets; tdd – triplet od doublet of doublets; m - multiplet; bs - broad signal.

Optical rotation of compounds was performed on a Jasco P-2000 digital polarimeter using WI (Tungsten-Halogen) lamp (λ = 589 nm). High resolution mass spectra (HRMS) were acquired using a Bruker solariX XR Fourier transform ion cyclotron resonance mass spectrometer (Bruker Daltonik GmbH, Bremen, Germany) equipped with a 7 T refrigerated actively-shielded superconducting magnet. The samples were ionized in positive ion mode using a MALDI ionization source. Melting points were measured with a Stuart Model SMP 30 melting point apparatus and are uncorrected.

All starting materials (unless otherwise noted) were purchased from Merck-SigmaAldrich or TCI-Europe and used as received.

Aldehydes were purchased from TCI-Europe and (phenylsulfonyl)acetonitrile, L-2-pyrrolidinemethanol, *N*,*N'*-dibenzylethylenediamine, ethylenediamine, *N*-benzylaminoethanol were purchased from Merck-SigmaAldrich and they were used as received. Enantiomeric ratio of heterocycles **3a-3j, 4a-4e** was determined by HPLC (Waters-Breeze 2487, UV dual λ absorbance detector and 1525 Binary HPLC Pump) using Daicel chiral columns. Catalysts **eQNU**[1] and **6d**[2] are known compound, which were prepared according to the literature.

[1] (a) Miyaji, R.; Asano K.; Matsubara, S. *Org. Lett.* **2013**, *15*, 3658. (b) Amere, M.; Lasne, M.-C.; Rouden, J. *Org. Lett.* **2007**, *9*, 2621. (c) Wu, W.; Min, L.; Zhu, L.; Lee, C.-S. *Adv. Synth. Catal.* **2013**, *353*, 1135.

[2] Meninno, S.; Zullo, L.; Overgaard, J.; Lattanzi, A. *Adv. Synth. Catal.* **2017**, *359*, 913.

## Experimental Procedures and Compounds Characterization

### General procedure for the racemic synthesis of heterocycles 3a-3j, 4a-4h

The starting alkenes are known compounds, they were prepared according to literature.[3,4]

In a sample vial containing the opportune alkene (0.10 mmol) and anhydrous toluene (0.5 mL), TBHP (~5.5 M in decane, 24 μL, 0.13 mmol) and triethylamine (4 μL, 0.030 mmol) were added. The reaction mixture was stirred until consumption of the alkene (TLC eluent PE/ethyl acetate 8/2). Then, the reaction was diluted with toluene (1.5 mL) and *N,N'*-dibenzylethylenediamine for the synthesis of dibenzylpiperazinones **3a-d**, **3i, j**, ethylenediamine for the synthesis of N-H free piperazinones **3e-h**, 2-benzylaminoethanol for the synthesis of morpholinones **4a-e** (0.12 mmol) and triethylamine (28 μL, 0.20 mmol) were added. The reaction mixture was stirred at room temperature (at 50 °C for compounds **3i, j**, by heating the reaction mixture with an oil bath), monitored by TLC and the crude was purified as indicated below for the corresponding enantioenriched products.

### General procedure for one-pot asymmetric Knoevenagel/epoxidation/ring-opening reaction to heterocycles 3a-3j, 4a-4h

In a sample vial containing (phenylsulfonyl)acetonitrile (18.5 mg, 0.10 mmol) and **eQNU** (5.8 mg, 0.010 mmol) in anhydrous toluene (0.35 mL), the opportune aldehyde **1a-d** (0.10 mmol) was added. The reaction was stirred at 30 °C for 10-30 hours (by heating the reaction mixture with an oil bath), monitored by TLC (eluent PE/ethyl acetate 8/2). After completion, toluene (4.7 mL) was added. The synthesis of dibenzylpiperazinones **3i, j** was carried out starting from aliphatic alkenes, which were synthesized from isovaleraldehyde and hexanal, respectively, according to literature,[4] because the catalyst poorly catalyzed the Knoevenagel reaction. Then 1.2 equivalents of cumene hydroperoxide (tech. 80%, 22 μL, 0.12 mmol) was added at – 20 °C and the solution was stirred at -20 °C until consumption of the alkene (TLC eluent PE/ ethyl acetate 8/2). Then *N,N'*-dibenzylethylenediamine for the synthesis of dibenzylpiperazinones **3a-d**, **3i, j**, ethylenediamine for the synthesis of N-H free piperazinones **3e-h**, 2-benzylaminoethanol or L-prolinol for the synthesis of morpholinones **4a-h** (0.12 mmol) and triethylamine (33 μL, 0.20 mmol) were added and the reaction mixture was stirred for 6-33 hours at room temperature (at 50 °C for compounds **3i, j,** by heating the reaction mixture with an oil bath), monitored by TLC: eluent hexane/ethyl acetate 8/2 to check the conversion of the epoxide. The following TLC conditions were used to visualize the cyclic products:

[3] (a) Pandit, K. S.; Kupwade, R. V.; Chavan, P. V.; Desai, U. V.; Wadgaonkar, P. P.; Kodam, K. M. *ACS Sustainable Chem. Eng.* **2016**, *4*, 3450; (b) Rajkumar, S.; Shankland, K.; Goodman, J. M.; Cobb, A. J. A. *Org. Lett.* **2013**, *15*, 1386; (c) Nemcsok, T.; Rapi, Z.; Bagi, P.; Guan, Y. H.; Orbán, I.; Keglevich, G.; Bakó, P. *Tetrahedron* **2020**, *76*, 130965.

[4] (a) Yamashita, K.; Tanaka, T.; Hayashi, M. *Tetrahedron* **2005**, *61*, 7981; (b) Volpe, C.; Meninno, S.; Crescenzi, C.; Mancinelli, M.; Mazzanti, A.; Lattanzi, A. *Angew. Chem. Int. Ed.* **2021**, *60*, 23819.

- For dibenzylpiperazinones **3a-d**, **3i, j**: TLC eluent: hexane/ethyl acetate 8/2 or 7/3. The products are visualized by UV light and ninhydrin stain (pink or red spots).
- For N-H free piperazinones **3e-h**: TLC eluent: inhibitor-free THF or $CH_2Cl_2$/MeOH 95/5. The products are visualized by UV light and ninhydrin stain (brown or red spots).
- For morpholinones **4a-h**: TLC eluent: hexane/ethyl acetate 8/2 (6/4 for **4d**). The products are visualized by UV light and phosphomolybdic acid (dark blue spots) or ninhydrin stain (red spots).

- Purification of dibenzylpiperazinones **3a-d**, **3i,j**:
  After completion, the mixture was diluted with ethyl acetate (10 mL) and washed with saturated $NH_4Cl$ solution (2x10 mL) and brine (1x10 mL), dried over anhydrous $Na_2SO_4$ and concentrated under reduced pressure. The reaction mixture was purified by flash chromatography (eluent: hexane/ethyl acetate 95/5 to 80/20) to give enantioenriched pure products **3a-d**, **3i,j** in 38-70% yield and 87-96% ee.
- Purification of N-H free piperazinones **3e-h**: the crude mixture was purified directly by flash chromatography (eluent: THF (inhibitor-free) to remove the catalyst and then $CH_2Cl_2$/MeOH 99/1 or 98/2 to recover the heterocyclic product) affording the products **3e-h** in 42-79% yield and 93-99% ee.
- Purification of morpholinones **4a-h**: the crude mixture was purified directly by flash chromatography (eluent: hexane/ethyl acetate 98/2 to 80/20) to give enantioenriched pure products **4a-h** in 53-85% yield and 63-89% ee (**4a-e**).

**Scale-up asymmetric synthesis of (*R*)-4-(1,4-dibenzyl-3-oxopiperazin-2-yl)benzonitrile (3c)**

In a sample vial containing (phenylsulfonyl)acetonitrile (185 mg, 1 mmol) and **eQNU** (58 mg, 0.10 mmol) in anhydrous toluene (3.5 mL), aldehyde **1c** (130 mg, 1 mmol) was added. The reaction was stirred at 30 °C for 10 hours (by heating the reaction mixture with an oil bath), monitored by TLC (eluent PE/ethyl acetate 7/3). After completion, toluene (46 mL) was added. Then, cumene hydroperoxide (tech. 80%, 220 μL, 1.2 mmol) was added at – 20 °C and the solution was stirred at - 20 °C until consumption of the alkene (TLC eluent PE/ ethyl acetate 7/3). Then *N*,*N'*-dibenzylethylenediamine (290 μL, 1.2 mmol) and triethylamine (330 μL, 2 mmol) were added and the reaction mixture was stirred for 24 hours at room temperature monitored by TLC (eluent hexane/ethyl acetate 7/3, the product **3c** was visualized by UV light and ninhydrin stain (red spot)). After completion, the mixture was diluted with ethyl acetate (50 mL) and washed with saturated $NH_4Cl$ solution (2 x 50 mL) and brine (1 x 50 mL), dried over anhydrous $Na_2SO_4$ and concentrated under reduced pressure. The reaction mixture was purified by flash chromatography (eluent:

hexane/ethyl acetate 90/10, then $CHCl_3$ to elute the product) to give the enantioenriched pure product **3c** in 84% yield (320 mg) and 94% ee.

**(*R*)-1,4-dibenzyl-3-phenylpiperazin-2-one (3a)**

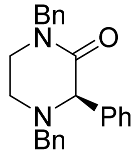


Data for this compound are consistent with those reported in the literature.[5]

Purified on flash silica gel (eluent: hexane/ethyl acetate 95/5 to 80/20). Yellow wax, 28.1 mg, 79% yield. $[\alpha]_D^{24}$ = -38.01 (c 0.26, $CHCl_3$), 91% ee. **$^1$H NMR** ($CDCl_3$, 600 MHz): $\delta$ 7.57 (d, J = 7.2 Hz, 2H), 7.40 (t, J = 7.6 Hz, 2H), 7.36 – 7.21 (m, 11H), 4.65 and 4.56 (ABq, J = 14.6 Hz, 2H), 4.15 (s, 1H), 3.76 (d, J = 13.5 Hz, 1H), 3.47 (td, J = 11.3, 4.1 Hz, 1H), 3.16 (d, J = 13.5 Hz, 1H), 3.12 (dt, J = 11.8, 3.3 Hz, 1H), 2.97 (dt, J = 12.1, 3.6 Hz, 1H), 2.49 (ddd, J = 12.1, 10.7, 3.6 Hz, 3H). **$^{13}$C{$^1$H} NMR** ($CDCl_3$, 150 MHz): $\delta$ 168.3, 139.4, 137.7, 136.8, 129.0, 128.7, 128.6, 128.4, 128.3, 128.2, 127.9, 127.5, 127.2, 71.2, 58.8, 50.2, 46.6, 45.8. HPLC analysis with Chiralcel OD column, 90:10 *n*-hexane:2-propanol, 0.5 mL/min, 220 nm; minor enantiomer $t_R$ = 28.7 min, major enantiomer $t_R$ = 23.5 min.

**(*R*)-1,4-dibenzyl-3-(4-chlorophenyl)piperazin-2-one (3b)**

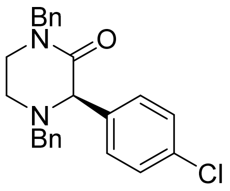


Data for this compound are consistent with those reported in the literature.[5c]

Purified on flash silica gel (eluent: hexane/ethyl acetate 95/5 to 80/20). Yellow wax, 27.3 mg, 70% yield. $[\alpha]_D^{24}$ = -48.0 (c 0.18, $CHCl_3$), 96% ee. **$^1$H NMR** ($CDCl_3$, 600 MHz): $\delta$ 7.52 (d, J = 8.4 Hz, 2H), 7.36 (d, J = 8.4 Hz, 2H), 7.34 – 7.27 (m, 5H), 7.26 – 7.19 (m, 5H), 4.62 and 4.53 (ABq, J = 14.6 Hz, 2H), 4.11 (s, 1H), 3.73 (d, J = 13.5 Hz, 1H), 3.46 (td, J = 11.2, 4.1 Hz, 1H), 3.15 (d, J = 13.5 Hz, 1H), 3.11 (dt, J = 11.7, 3.1 Hz, 1H), 2.96 (dt, J = 11.9, 3.5 Hz, 1H), 2.48 (td, J = 11.8, 3.5 Hz, 1H). **$^{13}$C{$^1$H} NMR** ($CDCl_3$, 150 MHz): $\delta$ 167.9, 138.0, 137.4, 136.6, 133.7, 130.3, 128.71, 128.67, 128.66, 128.34, 128.2, 127.6, 127.4, 70.5, 58.9, 50.3, 46.7, 45.8. HPLC analysis with Chiralcel OD-H column, 90:10 n-hexane:2-propanol, 1 mL/min, 220 nm; minor enantiomer $t_R$ = 15.0 min, major enantiomer $t_R$ = 12.1 min.

---

[5] (a) Baek, J.; Jang J. I.; Park, Y. S. *Bull. Korean Chem. Soc.* **2011**, *32*, 4067; (b) Jang, J. I.; Kang, S. Y.; Kang, K. H.; Park, Y. S. *Tetrahedron* **2011**, *67*, 6221; (c) Meninno, S.; Vidal-Albalat,A.; Lattanzi, A. *Org. Lett.* **2015**, *17*, 4348.

**(*R*)-4-(1,4-dibenzyl-3-oxopiperazin-2-yl)benzonitrile (3c)**

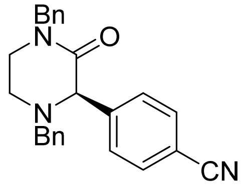


Data for this compound are consistent with those reported in the literature.[5c]

Purified on flash silica gel (eluent: hexane/ethyl acetate 95/5 to 80/20). Yellow wax, 34.3 mg, 90% yield. $[\alpha]_D^{26}$ = -73.7 (c 0.18, $CHCl_3$), 94% ee. **$^1$H NMR** ($CDCl_3$, 600 MHz): $\delta$ 7.73 (d, J = 8.2 Hz, 2H), 7.69 (d, J = 8.2 Hz, 2H), 7.36 – 7.27 (m, 5H), 7.25 – 7.18 (m, 5H), 4.63 and 4.51 (ABq, J = 14.6 Hz, 2H), 4.20 (s, 1H), 3.68 (d, J = 13.5 Hz, 1H), 3.48 (td, J = 11.4, 4.0 Hz, 1H), 3.19 (d, J = 13.5 Hz, 1H), 3.14 (dt, J = 11.9, 2.9 Hz, 1H), 2.99 (dt, J = 12.1, 3.4 Hz, 1H), 2.52 (td, J = 11.9, 3.5 Hz, 1H). **$^{13}$C{$^1$H} NMR** ($CDCl_3$, 150 MHz): $\delta$ 167.0, 145.1, 136.9, 136.3, 132.2, 129.7, 128.7, 128.6, 128.4, 128.1, 127.7, 127.5, 118.8, 111.7, 70.7, 59.1, 50.3, 46.7, 45.7. HPLC analysis with Chiralcel OD-H column, 80:20 n-hexane:2-propanol, 1 mL/min, 220 nm; minor enantiomer $t_R$ = 24.4 min, major enantiomer $t_R$ = 14.9 min.

**(*R*)-3-(4-bromophenyl)piperazin-2-one (3d)**

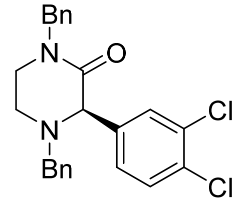


Purified on flash silica gel (eluent: hexane/ethyl acetate 95/5 to 80/20). Yellow wax, 31 mg, 73% yield. $[\alpha]_D^{24}$ = -56.7 (c 0.49, $CHCl_3$), 94% ee. **$^1$H NMR** ($CDCl_3$, 600 MHz): $\delta$ 7.70 (d, J = 1.8 Hz, 1H), 7.47-7.46 (m, 1H), 7.43 (dd, J = 8.2, 1.8 Hz, 1H), 7.35 – 7.27 (m, 5H), 7.25 – 7.19 (m, 5H), 4.64 and 4.50 (ABq, J = 14.6 Hz, 2H), 4.10 (s, 1H), 3.73 (d, J = 13.4 Hz, 1H), 3.46 (td, J = 11.4, 4.0 Hz, 1H), 3.17 (d, J = 13.5 Hz, 1H), 3.12 (dt, J = 11.9, 3.1 Hz, 1H), 2.97 (dt, J = 12.1, 3.5 Hz, 1H), 2.49 (td, J = 12.0, 3.6 Hz, 1H). **$^{13}$C{$^1$H} NMR** ($CDCl_3$, 150 MHz): $\delta$ 167.3, 139.8, 137.0, 136.5, 132.6, 131.9, 130.8, 130.4, 128.7, 128.4, 128.2, 127.7, 127.5, 70.0, 59.0, 50.3, 46.6, 45.7. HPLC analysis with Chiralcel OD-H column, 90:10 n-hexane:2-propanol, 1 mL/min, 220 nm; minor enantiomer $t_R$ = 18.5 min, major enantiomer $t_R$ = 13.4 min. **HRMS (MALDI-FT ICR)** m/z $[M+H]^+$ calcd for $C_{24}H_{23}{}^{35}Cl_2N_2O$: 425.1182, found: 425.1212; calcd for $C_{24}H_{23}{}^{35}Cl\,{}^{37}ClN_2O$: 427.1158, found: 427.1189; calcd for $C_{24}H_{23}{}^{37}Cl_2N_2O$: 429.1141, found: 429.1172.

**(*R*)-3-(4-bromophenyl)piperazin-2-one (3e)**

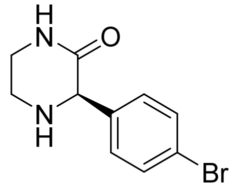


Data for this compound are consistent with those reported in the literature.[6]

Purified on flash silica gel (eluent: THF (inhibitor-free) to remove the catalyst and then $CH_2Cl_2$/MeOH 98/2 to recover the heterocyclic product. White solid, 20.2 mg, 79% yield. $[\alpha]_D^{24}$ = -32.2 (c 0.35, $CHCl_3$), 95% ee. **$^1$H NMR** ($CDCl_3$, 600 MHz): $\delta$ 7.47 (d, J = 8.3 Hz, 2H), 7.32 (d, J = 8.3 Hz, 2H), 7.00 (s, 1H), 4.51 (s, 1H), 3.54 – 3.42 (m, 1H), 3.36 – 3.27 (m, 1H), 3.13 (dt, J = 11.9, 4.0 Hz, 1H), 3.05 (ddd, J = 12.6, 9.0, 4.0 Hz, 1H). **$^{13}$C{$^1$H} NMR** ($CDCl_3$, 150 MHz): $\delta$ 170.4, 138.3, 131.5, 130.1, 121.9, 63.2, 43.1, 41.2. HPLC analysis with Chiralpak IC column, 80:20 n-hexane:2-propanol, 1 mL/min, 220 nm; minor enantiomer $t_R$ = 39.9 min, major enantiomer $t_R$ = 29.6 min.

**(*R*)-3-(p-tolyl)piperazin-2-one (3f)**

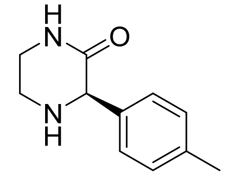


Data for this compound are consistent with those reported in the literature.[6]

Purified on flash silica gel (eluent: THF (inhibitor-free) to remove the catalyst and then $CH_2Cl_2$/MeOH 99/1 to recover the heterocyclic product. White wax, 8.0 mg, 42% yield. $[\alpha]_D^{24}$ = -18.1 (c 0.36, $CHCl_3$), 96% ee. **$^1$H NMR** ($CDCl_3$, 600 MHz): $\delta$ 7.30 (d, J = 7.9 Hz, 2H), 7.16 (d, J = 7.8 Hz, 2H), 6.39 (bs, 1H), 4.54 (s, 1H), 3.58 – 3.50 (m, 1H), 3.42 – 3.33 (m, 1H), 3.16 (dt, J = 12.5, 4.3 Hz, 1H), 3.06 (ddd, J = 12.7, 8.7, 4.1 Hz, 1H), 2.33 (s, 3H). **$^{13}$C{$^1$H} NMR** ($CDCl_3$, 150 MHz): $\delta$ 170.9, 137.6, 136.3, 129.2, 128.2, 63.8, 43.4, 41.2, 21.1. HPLC analysis with Chiralcel OD-H column, 90:10 n-hexane:2-propanol, 1 mL/min, 210 nm; minor enantiomer $t_R$ = 39.9 min, major enantiomer $t_R$ = 43.0 min.

**(*R*)-1,4-dibenzyl-3-(3,4-dichlorophenyl)piperazin-2-one (3g)**

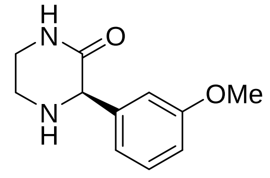

Data for this compound are consistent with those reported in the literature.[6]

Purified on flash silica gel (eluent: THF (inhibitor-free) to remove the catalyst and then $CH_2Cl_2$/MeOH 98/2 to recover the heterocyclic product. Pale yellow wax, 12.2 mg, 59% yield. $[\alpha]_D^{26}$ = -33.3 (c 0.14, $CHCl_3$), 98% ee. **$^1$H NMR** ($CDCl_3$, 600 MHz): $\delta$ 7.29 – 7.25 (m, 1H), 7.03 – 6.96 (m, 2H), 6.85 (dd, J = 8.2, 2.0 Hz, 1H), 6.51 (Bs, 1H), 4.56 (s, 1H), 3.81 (s, 3H), 3.57 – 3.50 (m, 1H), 3.42 – 3.35 (m, 1H), 3.17 (dt, J = 12.3, 4.3 Hz, 1H), 3.07 (ddd, J = 12.6, 8.6, 4.2 Hz, 1H). **$^{13}$C{$^1$H} NMR** ($CDCl_3$, 150 MHz): $\delta$ 170.5, 159.7, 140.6, 129.6, 120.7, 113.9, 113.6, 63.8, 55.2, 43.2, 41.0. HPLC analysis with Chiralpak AD-H column, 90:10 n-hexane:2-propanol, 1 mL/min, 210 nm; minor enantiomer $t_R$ = 32.7 min, major enantiomer $t_R$ = 33.6 min.

**(*R*)-3-(naphthalen-2-yl)piperazin-2-one (3h)**

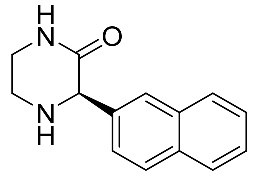

Data for this compound are consistent with those reported in the literature.[6]

Purified on flash silica gel (eluent: THF (inhibitor-free) to remove the catalyst and then $CH_2Cl_2$/MeOH 98/2 to recover the heterocyclic product. White solid, 11.3 mg, 50% yield. $[\alpha]_D^{26}$ = -28.8 (c 0.35, $CHCl_3$), 99% ee. **$^1$H NMR** ($CDCl_3$, 600 MHz): $\delta$ 7.91 – 7.78 (m, 4H), 7.56 (d, J = 8.3 Hz, 1H), 7.49 – 7.42 (m, 2H), 6.09 (bs, 1H), 4.76 (s, 1H), 3.66 – 3.57 (m, 1H), 3.47 – 3.40 (m, 1H), 3.22 (dt, J = 12.3, 4.5 Hz, 1H), 3.14 (ddd, J = 12.6, 8.8, 4.1 Hz, 1H). **$^{13}$C{$^1$H} NMR** ($CDCl_3$, 150 MHz): $\delta$ 170.5, 136.6, 133.3, 133.1, 128.4, 128.0, 127.6, 127.4, 126.2, 126.1, 126.0, 64.2, 43.5, 41.3. HPLC analysis with Chiralpak AD-H column, 80:20 n-hexane:2-propanol, 1 mL/min, 220 nm; minor enantiomer $t_R$ = 16.9 min, major enantiomer $t_R$ = 14.9 min.

**(*R*)-1,4-dibenzyl-3-isobutylpiperazin-2-one (3i)**

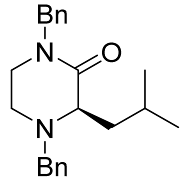

Purified on flash silica gel (eluent: hexane/ethyl acetate 95/5 to 90/10). Pale yellow liquid, 13.5 mg, 40% yield. $[\alpha]_D^{24}$ = -4.7 (c 0.44, $CHCl_3$), 90% ee. **$^1$H NMR** ($CDCl_3$, 600 MHz): $\delta$ 7.38 – 7.26 (m, 10H), 4.66 and 4.56 (ABq, J = 14.5 Hz, 2H), 3.86 (d, J = 13.2 Hz, 1H), 3.50 (d, J = 13.2 Hz, 1H), 3.29 (ddd, J = 12.2, 7.9, 4.5 Hz, 1H), 3.25 – 3.23 (dd, J = J = 6.1 Hz, 1H), 3.13 – 3.07 (m, 1H), 3.04 (ddd, J = 13.0, 8.0, 4.6 Hz, 1H), 2.51 (dt, J = 13.0, 4.8 Hz, 1H), 1.94 (tt, J = 13.3, 6.7 Hz, 1H), 1.86

[6] Wang,Y.; Liu, Y.; Li, K.; Yang, G.; Zhang, W. *Adv. Synth. Catal.* **2017**, *359*, 1933.

(dt, J = 13.6, 6.7 Hz, 1H), 1.74 (ddd, J = 13.5, 7.7, 5.5 Hz, 1H), 0.92 (d, J = 6.6 Hz, 3H), 0.85 (d, J = 6.6 Hz, 3H). **$^{13}$C{$^{1}$H} NMR** ($CDCl_3$, 150 MHz): $\delta$ 171.2, 138.2, 137.0, 128.8, 128.6, 128.3, 128.2, 127.5, 127.3, 62.9, 58.0, 50.1, 43.8, 43.4, 39.6, 24.9, 22.9, 22.3. HPLC analysis with Chiralcel OD-H column, 90:10 n-hexane:2-propanol, 1 mL/min, 220 nm; minor enantiomer $t_R$ = 10.5 min, major enantiomer $t_R$ = 7.8 min. **HRMS (MALDI-FT ICR)** m/z $[M+H]^+$ calcd for $C_{22}H_{29}N_2O$: 337.2274, found: 337.2303.

**(*R*)-1,4-dibenzyl-3-pentylpiperazin-2-one (3j)**

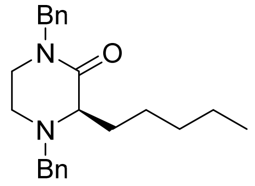


Purified on flash silica gel (eluent: hexane/ethyl acetate 95/5 to 90/10). Yellow liquid, 11.9 mg, 34% yield. $[\alpha]_D^{24}$ = -2.6 (c 0.50, $CHCl_3$), 87% ee. **$^{1}$H NMR** ($CDCl_3$, 300 MHz): $\delta$ 7.39 – 7.19 (m, 10H), 4.70 and 4.52 (ABq, J = 14.6 Hz, 2H), 3.94 (d, J = 13.5 Hz, 1H), 3.33 (d, J = 13.4 Hz, 1H), 3.26 – 3.04 (m, 3H), 2.92 (dt, J = 12.4, 4.5 Hz, 1H), 2.41 (ddd, J = 12.4, 8.4, 4.1 Hz, 1H), 2.07 (ddd, J = 15.9, 10.4, 4.8 Hz, 1H), 1.95 – 1.76 (m, 1H), 1.65 – 1.47 (m, 1H), 1.45 – 1.16 (m, 5H), 0.89 (t, J = 6.8 Hz, 3H). **$^{13}$C{$^{1}$H} NMR** ($CDCl_3$, 75 MHz): $\delta$ 170.3, 138.2, 136.9, 128.8, 128.6, 128.3, 128.1, 127.4, 127.2, 65.0, 58.3, 50.2, 45.7, 44.7, 31.9, 30.5, 24.9, 22.7, 14.1. HPLC analysis with Chiralcel OD-H column, 90:10 n-hexane:2-propanol, 1 mL/min, 220 nm; minor enantiomer $t_R$ = 8.7 min, major enantiomer $t_R$ = 7.4 min. **HRMS (MALDI-FT ICR)** m/z $[M+H]^+$ calcd for $C_{23}H_{31}N_2O$: 351.2431, found: 351.2460.

**(*R*)-4-benzyl-3-(m-tolyl)morpholin-2-one (4a)**

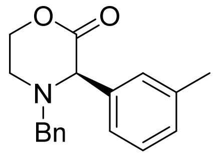


Purified on flash silica gel (eluent: hexane/ethyl acetate 98/2 to 90/10). Colorless wax, 19.7 mg, 70% yield. $[\alpha]_D^{26}$ = -98.1 (c 0.50, $CHCl_3$), 89% ee. **$^{1}$H NMR** ($CDCl_3$, 600 MHz): $\delta$ 7.39-7.37 (m, 2H), 7.34 – 7.23 (m, 6H), 7.16 (d, J = 7.5 Hz, 1H), 4.56 (td, J = 11.0, 3.1 Hz, 1H), 4.37 (ddd, J = 10.8, 3.0, 2.4 Hz, 1H), 4.22 (s, 1H), 3.79 (d, J = 13.4 Hz, 1H), 3.17 (d, J = 13.4 Hz, 1H), 3.00 (dt, J = 12.8, 2.7 Hz, 1H), 2.64 (ddd, J = 12.8, 11.3, 3.2 Hz, 1H), 2.40 (s, 3H). **$^{13}$C{$^{1}$H} NMR** ($CDCl_3$, 150 MHz): $\delta$ 168.9, 138.3, 137.3, 137.0, 129.7, 129.2, 128.8, 128.6, 128.4, 127.5, 125.9, 70.6, 68.6, 58.8, 46.8, 21.5. HPLC analysis with Chiralcel OD-H column, 90:10 n-hexane:2-propanol, 1 mL/min, 220 nm; minor enantiomer $t_R$ = 14.8 min, major enantiomer $t_R$ = 16.4 min. **HRMS (MALDI-FT ICR)** m/z $[M+H]^+$ calcd for $C_{18}H_{20}NO_2$: 282.1489, found: 282.1499.

**(*R*)-3-([1,1'-biphenyl]-4-yl)-4-benzylmorpholin-2-one (4b)**

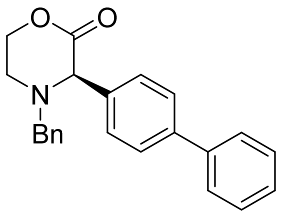

Purified on flash silica gel (eluent: hexane/ethyl acetate 98/2 to 90/10). White solid, 29.2 mg, 85% yield, mp 103 °C (Decomp.). $[\alpha]_D^{24}$ = -92.0 (c 0.55, $CHCl_3$), 86% ee. **$^1$H NMR** ($CDCl_3$, 400 MHz): $\delta$ 7.71 – 7.54 (m, 6H), 7.44 (t, J = 7.6 Hz, 2H), 7.39 – 7.23 (m, 6H), 4.59 (td, J = 11.0, 2.8 Hz, 1H), 4.39 (dt, J = 10.8, 2.5 Hz, 1H), 4.31 (s, 1H), 3.84 (d, J = 13.4 Hz, 1H), 3.21 (d, J = 13.4 Hz, 1H), 3.03 (dt, J = 12.8, 2.5 Hz, 1H), 2.67 (td, 12.8, 2.8 Hz, 1H). **$^{13}$C{$^1$H} NMR** ($CDCl_3$, 100 MHz): $\delta$ 168.8, 141.4, 140.6, 136.9, 136.4, 129.3, 128.8, 128.7, 128.4, 127.51, 127.47, 127.3, 127.1, 70.2, 68.7, 58.8, 46.8. HPLC analysis with Chiralcel OD-H column, 90:10 n-hexane:2-propanol, 1 mL/min, 254 nm; minor enantiomer $t_R$ = 21.1 min, major enantiomer $t_R$ = 26.2 min. **HRMS (MALDI-FT ICR)** m/z $[M+H]^+$ calcd for $C_{23}H_{22}NO_2$: 344.1645, found: 344.1673.

**(*R*)-4-benzyl-3-(4-fluorophenyl)morpholin-2-one (4c)**

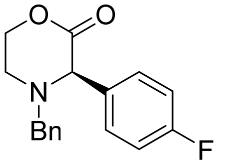

Data for this compound are consistent with those reported in the literature.[7]

(*S*)-**4c** was prepared according to general procedure for one-pot asymmetric Knoevenagel/epoxidation/ring-opening reaction to heterocycles **3a-3j**, **4a-4h** by using catalyst **6d** (6.8 mg, 10 mol%) and starting from (phenylsulfonyl)acetonitrile (18.5 mg, 0.10 mmol) and p-fluorobenzaldehyde **1c** (11 µL, 0.10 mmol). They were reacted in toluene (0.35 mL) at room temperature for 19 h. When the formation of the alkene was completed, the mixture was diluted with toluene (4.7 mL) and 1.2 equivalents of cumene hydroperoxide (tech. 80%, 22 µL, 0.12 mmol) was added at – 50 °C and the solution was stirred at -50 °C for 43 h. Then 2-benzylaminoethanol (95%, 18 µL, 0.12 mmol) and triethylamine (33 µL, 0.20 mmol) were added and the reaction mixture was stirred for 8 h at room temperature, monitored by TLC (eluent: hexane/ethyl acetate 7/3 – visualized by UV light and phosphomolybdic acid (dark blue spot), Rf ≈ 0.5). The crude mixture was purified directly by flash chromatography (eluent: hexane/ethyl acetate 95/5 to 90/10) to give enantioenriched pure morpholinone (*S*)-**4c** in 65% yield and 74% ee.

[7] Mazuela, J.; Antonsson, T.; Johansson, M. J.; Knerr, L.; Marsden, S. P. *Org. Lett.* **2017**, *19*, 5541.

Purified on flash silica gel (eluent: hexane/ethyl acetate 95/5 to 90/10). Pale yellow liquid, 20.2 mg, 71% yield. $[\alpha]_D^{24}$ = -97.0 (c 0.50, $CHCl_3$), 89% ee (*R*); $[\alpha]_D^{24}$ = +87.7 (c 0.50, $CHCl_3$), 82% ee (*S*). **$^1$H NMR** ($CDCl_3$, 600 MHz): $\delta$ 7.59 – 7.54 (m, 2H), 7.32 (m, 2H), 7.28 (d, J = 7.2 Hz, 1H), 7.24 (d, J = 7.2 Hz, 2H), 7.10 (t, J = 8.6 Hz, 2H), 4.54 (td, J = 11.1, 3.1 Hz, 1H), 4.37 (dt, J = 11.0, 2.6 Hz 1H), 4.24 (s, 1H), 3.76 (d, J = 13.4 Hz, 1H), 3.17 (d, J = 13.4 Hz, 1H), 3.00 (dt, J = 12.9, 2.6 Hz, 1H), 2.66 (ddd, J = 12.9, 11.4, 3.2 Hz, 1H). **$^{13}$C{$^1$H} NMR** ($CDCl_3$, 150 MHz): $\delta$ 168.6, 162.7 (d, $J^1_{CF}$ = 248 Hz), 136.7, 133.2 (q, $J^4_{CF}$ = 3 Hz), 130.5 (d, $J^3_{CF}$ = 8 Hz), 128.8, 128.5, 127.6, 115.7 (d, $J^2_{CF}$ = 21 Hz), 69.7, 68.6, 58.8, 46.9. HPLC analysis with Chiralpak AD-H column, 90:10 n-hexane:ethanol 1 mL/min, 220 nm; minor enantiomer (*S*) $t_R$ = 17.4 min, major enantiomer (*R*) $t_R$ = 24.8 min.

**(*R*)-4-benzyl-3-(3-nitrophenyl)morpholin-2-one (4d)**

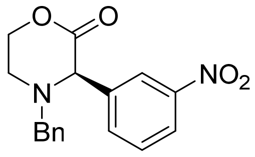

Purified on flash silica gel (eluent: hexane/ethyl acetate 95/5 to 80/20). Yellow wax, 24.4 mg, 78% yield. $[\alpha]_D^{24}$ = -68.8 (c 0.50, $CHCl_3$), 70% ee. **$^1$H NMR** ($CDCl_3$, 600 MHz): $\delta$ 8.53 (t, J = 1.8 Hz, 1H), 8.23-8.21 (m, 1H), 7.93 (d, J = 7.7 Hz, 1H), 7.59 (t, J = 7.9 Hz, 1H), 7.34-7.32 (m, 2H), 7.29-7.26 (m, 1H), 7.24-7.23 (m, 2H), 4.60 (td, J = 11.3, 3.0 Hz, 1H), 4.42 (dt, J = 11.0, 2.5 Hz, 1H), 4.39 (s, 1H), 3.74 (d, J = 13.3 Hz, 1H), 3.25 (d, J = 13.4 Hz, 1H), 3.06 (dt, J = 12.9, 2.5 Hz, 1H), 2.73 (ddd, J = 13.1, 11.6, 3.2 Hz, 1H). **$^{13}$C{$^1$H} NMR** ($CDCl_3$, 150 MHz): $\delta$ 167.5, 148.5, 139.7, 136.0, 135.2, 129.7, 128.8, 128.7, 127.9, 123.63, 123.57, 69.5, 68.7, 59.2, 46.9. HPLC analysis with Chiralpak AD-H column, 90:10 n-hexane:ethanol, 1 mL/min, 220 nm; minor enantiomer $t_R$ = 33.1 min, major enantiomer $t_R$ = 35.0 min. **HRMS (MALDI-FT ICR)** m/z $[M+H]^+$ calcd for $C_{17}H_{17}N_2O_4$: 313.1183, found: 313.1190.

**(*R*)-4-benzyl-3-(naphthalen-1-yl)morpholin-2-one (4e)**

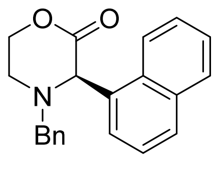

Purified on flash silica gel (eluent: hexane/ethyl acetate 95/5 to 90/10). Colorless wax, 23.2 mg, 73% yield. $[\alpha]_D^{24}$ = -79.3 (c 0.50, $CHCl_3$), 63% ee. **$^1$H NMR** ($CDCl_3$, 600 MHz): $\delta$ 8.39 (d, J = 8.5 Hz, 1H), 7.88 (dd, J = 12.3, 8.4 Hz, 2H), 7.71 (d, J = 7.0 Hz, 1H), 7.61 – 7.55 (m, 1H), 7.54 – 7.46 (m, 2H), 7.25 – 7.18 (m, 3H), 7.11 (d, J = 6.9 Hz, 2H), 4.77 (td, J = 11.4, 3.3 Hz, 1H), 4.73 (s, 1H), 4.48 (ddd, J = 11.0, 3.2, 1.6 Hz, 1H), 3.71 (d, J = 13.3 Hz, 1H), 3.08 (d, J = 13.3 Hz, 1H)

overlapped with 3.12-3.04 (m, 1H), 2.69 (td, J = 12.4, 3.4 Hz, 1H). **$^{13}C\{^1H\}$ NMR** ($CDCl_3$, 150 MHz): $\delta$ 168.5, 137.1, 134.4, 132.9, 131.4, 129.6, 129.0, 128.88, 128.85, 128.4, 127.4, 126.3, 125.9, 125.1, 124.5, 70.3, 68.7, 58.9, 47.7. HPLC analysis with Chiralpak AS-H column, 70:30 n-hexane:2-propanol, 0.8 mL/min, 220 nm; minor enantiomer $t_R$ = 24.4 min, major enantiomer $t_R$ = 19.4 min. **HRMS (MALDI-FT ICR)** m/z $[M+H]^+$ calcd for $C_{21}H_{20}NO_2$: 318.1489, found: 318.1518.

**(4*R*,8a*S*)-4-phenyltetrahydro-1H-pyrrolo[2,1-c][1,4]oxazin-3(4H)-one (4f)**

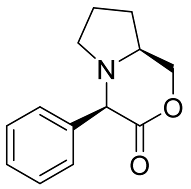

Data for this compound are consistent with those reported in the literature.[8]

Purified on flash silica gel (eluent: hexane/ethyl acetate 90/10 to 80/20). Yellow wax, 11.5 mg, 53% yield. $[\alpha]_D^{24}$ = -106.3 (c 0.25, $CH_2Cl_2$), dr >99/1, >99% ee. **$^1H$ NMR** ($CDCl_3$, 600 MHz): $\delta$ 7.42 (m, 2H), 7.38 – 7.29 (m, 3H), 4.53 (dd, J = 10.2, 3.3 Hz, 1H), 4.33 (dd, J = J = 10.4 Hz, 1H), 4.07 (s, 1H), 2.88 (td, J = 8.8, 2.5 Hz, 1H), 2.78 (tdd, J = 10.1, 6.6, 3.3 Hz, 1H), 2.07 (dd, J = 18.0, 9.0 Hz, 1H), 2.02 – 1.80 (m, 3H), 1.54 (ddd, J = 21.3, 11.5, 6.7 Hz, 1H). **$^{13}C\{^1H\}$ NMR** ($CDCl_3$, 150 MHz): $\delta$ 168.9, 137.4, 128.7, 128.5, 128.3, 73.6, 72.2, 58.5, 53.0, 26.3, 22.3.

**(4*R*,8a*S*)-4-(4-bromophenyl)tetrahydro-1H-pyrrolo[2,1-c][1,4]oxazin-3(4H)-one (4g)**

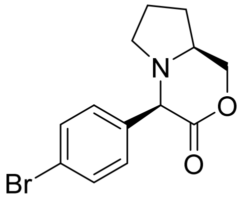

Data for this compound are consistent with those reported in the literature.[8]

Purified on flash silica gel (eluent: hexane/ethyl acetate 90/10 to 80/20). Pale yellow solid, 17.8 mg, 60% yield. $[\alpha]_D^{24}$ = -111.2 (c 0.38, $CH_2Cl_2$), dr >99/1, >99% ee. **$^1H$ NMR** ($CDCl_3$, 600 MHz): $\delta$ 7.48 (d, J = 8.3 Hz, 2H), 7.31 (d, J = 8.3 Hz, 2H), 4.52 (dd, J = 10.2, 3.1 Hz, 1H), 4.30 (dd, J = J = 10.4 Hz, 1H), 4.03 (s, 1H), 2.86 (td, J = 8.9, 2.3 Hz, 1H), 2.77 (tdd, J = 10.0, 6.6, 3.2 Hz, 1H), 2.04 (dd, J = 17.9, 8.9 Hz, 1H), 2.01 – 1.80 (m, 3H), 1.53 (ddd, J = 21.5, 11.3, 6.9 Hz, 1H). **$^{13}C\{^1H\}$ NMR** ($CDCl_3$, 150 MHz): $\delta$ 168.2, 136.4, 131.6, 130.3, 122.2, 73.6, 71.4, 58.4, 52.9, 26.2, 22.2.

[8] Mlostoń, G.; Wróblewska, A.; Linden, A.; Heimgartner, H. *Asian J. Org. Chem.* **2015**, *4*, 770.

**(4*R*,8a*S*)-4-(4-isopropylphenyl)tetrahydro-1H-pyrrolo[2,1-c][1,4]oxazin-3(4H)-one (4h)**

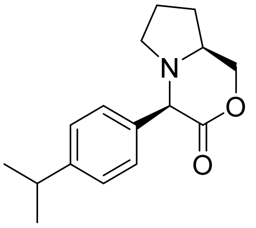


Purified on flash silica gel (eluent: hexane/ethyl acetate 90/10 to 80/20). Colorless solid, 14.5 mg, 56% yield. **Mp** 75.2 – 78.2 °C. $[\alpha]_D^{24}$ = -104.4 (c 0.5, $CHCl_3$), dr >99/1, >99% ee. **$^1$H NMR** ($CDCl_3$, 300 MHz): $\delta$ 7.32 (d, J = 8.1 Hz, 2H), 7.20 (d, J = 8.1 Hz, 2H), 4.52 (dd, J = 10.1, 3.3 Hz, 1H), 4.32 (dd, J = J = 10.3 Hz, 1H), 4.04 (s, 1H), 2.98 – 2.83 (m, 2H), 2.77 (tdd, J = 10.1, 6.5, 3.2 Hz, 1H), 2.06 (dd, J = 17.7, 8.9 Hz, 1H), 2.00 – 1.77 (m, 3H), 1.64 – 1.44 (m, 1H), 1.24 (d, J = 6.9 Hz, 6H). **$^{13}$C{$^1$H} NMR** ($CDCl_3$, 75 MHz): $\delta$ 169.1, 148.8, 134.7, 128.5, 126.6, 73.6, 72.0, 58.5, 53.1, 33.8, 26.2, 23.91, 23.88, 22.2. **HRMS (MALDI-FT ICR)** m/z $[M+H]^+$ calcd for $C_{16}H_{22}NO_2$: 260.1645, found: 260.1631.

**(2*R*,3*R*)-3-(4-fluorophenyl)-2-(phenylsulfonyl)oxirane-2-carbonitrile (7)**

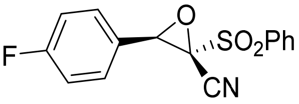


Purified on flash silica gel (eluent: hexane/ethyl acetate 100/0 to 85/15). Yellow wax, 15.2 mg, 50% yield. $[\alpha]_D^{24}$ = +10.5 (c 0.25, $CHCl_3$), 78% ee. **$^1$H NMR** ($CDCl_3$, 600 MHz): $\delta$ 8.09 – 8.01 (m, 2H), 7.88 – 7.81 (m, 1H), 7.71 (t, J = 8.0 Hz, 2H), 7.43 – 7.37 (m, 2H), 7.15 (t, J = 8.6 Hz, 2H), 5.03 (s, 1H). **$^{13}$C{$^1$H} NMR** ($CDCl_3$, 150 MHz): $\delta$ 164.2 (d, $^1J_{CF}$ = 249 Hz), 136.0, 134.2, 129.9, 128.8 (d, $^3J_{CF}$ = 9 Hz), 124.4 (d, $^4J_{CF}$ = 3 Hz), 116.4 (d, $^2J_{CF}$ = 23 Hz), 115.3, 110.9, 67.4, 63.1. HPLC analysis with Chiralcel OD column, 90:10 *n*-hexane:2-propanol, 1 mL/min, 254 nm; minor enantiomer $t_R$ = 11.0 min, major enantiomer $t_R$ = 10.1 min. **HRMS (MALDI-FT ICR)** m/z $[M+H]^+$ calcd for $C_{15}H_{11}FNO_3S$: 304.0365, found: 304.0372.

**NMR spectra**

$^{1}H$ NMR in $CDCl_3$ (600 MHz)

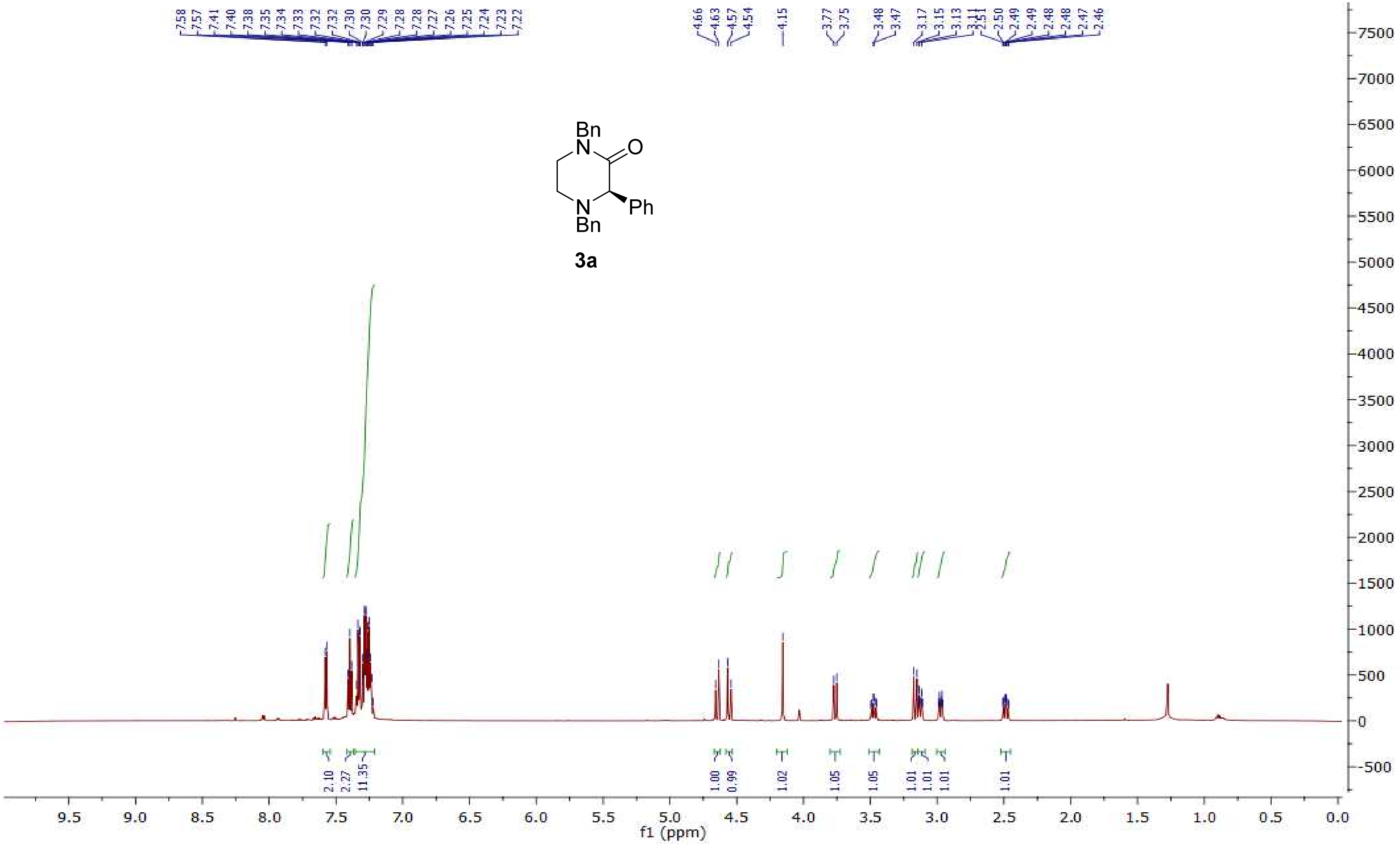

**$^{13}$C{$^{1}$H}** NMR in $CDCl_3$ (150 MHz)

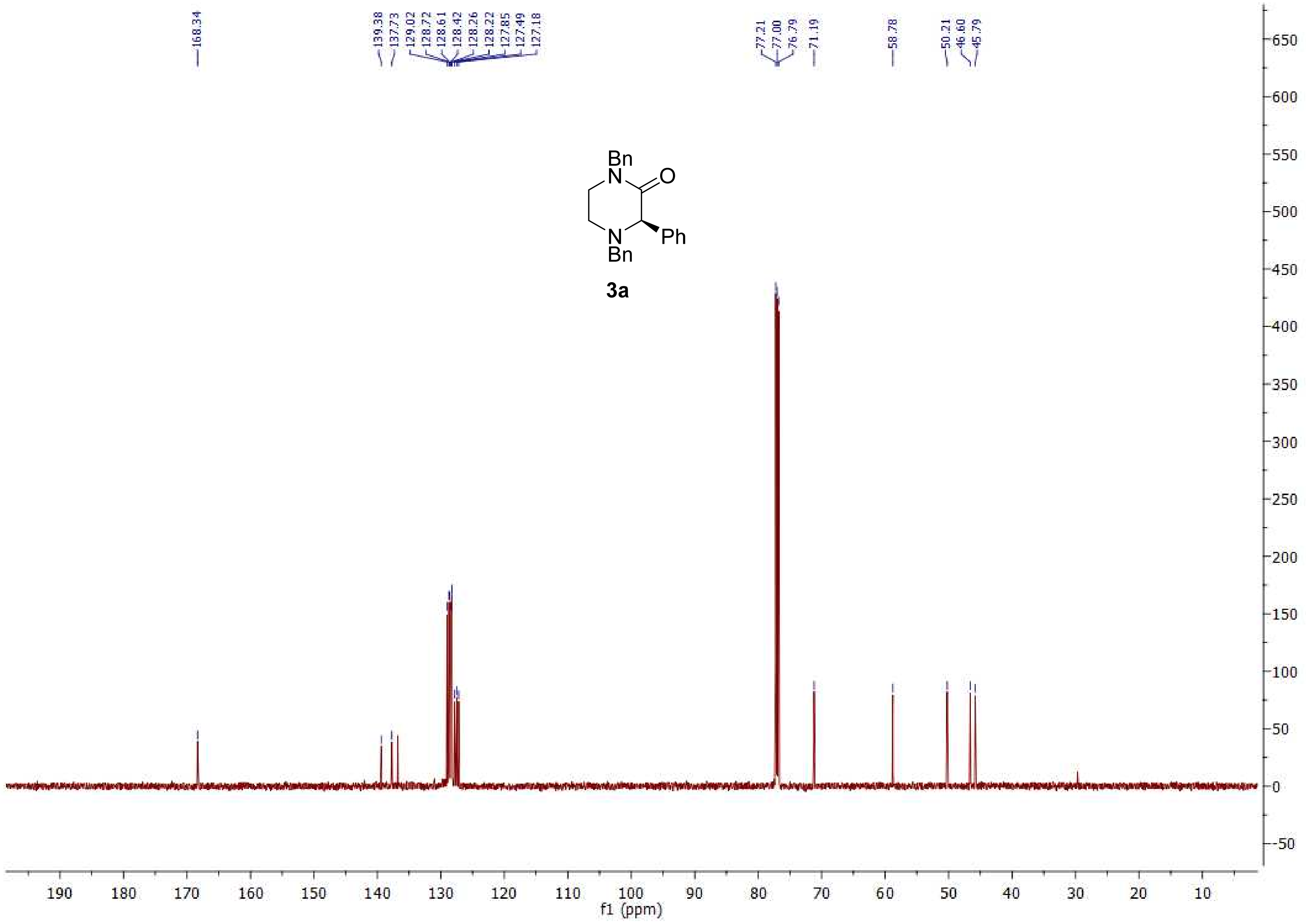

$^{1}$H NMR in $CDCl_3$ (600 MHz)

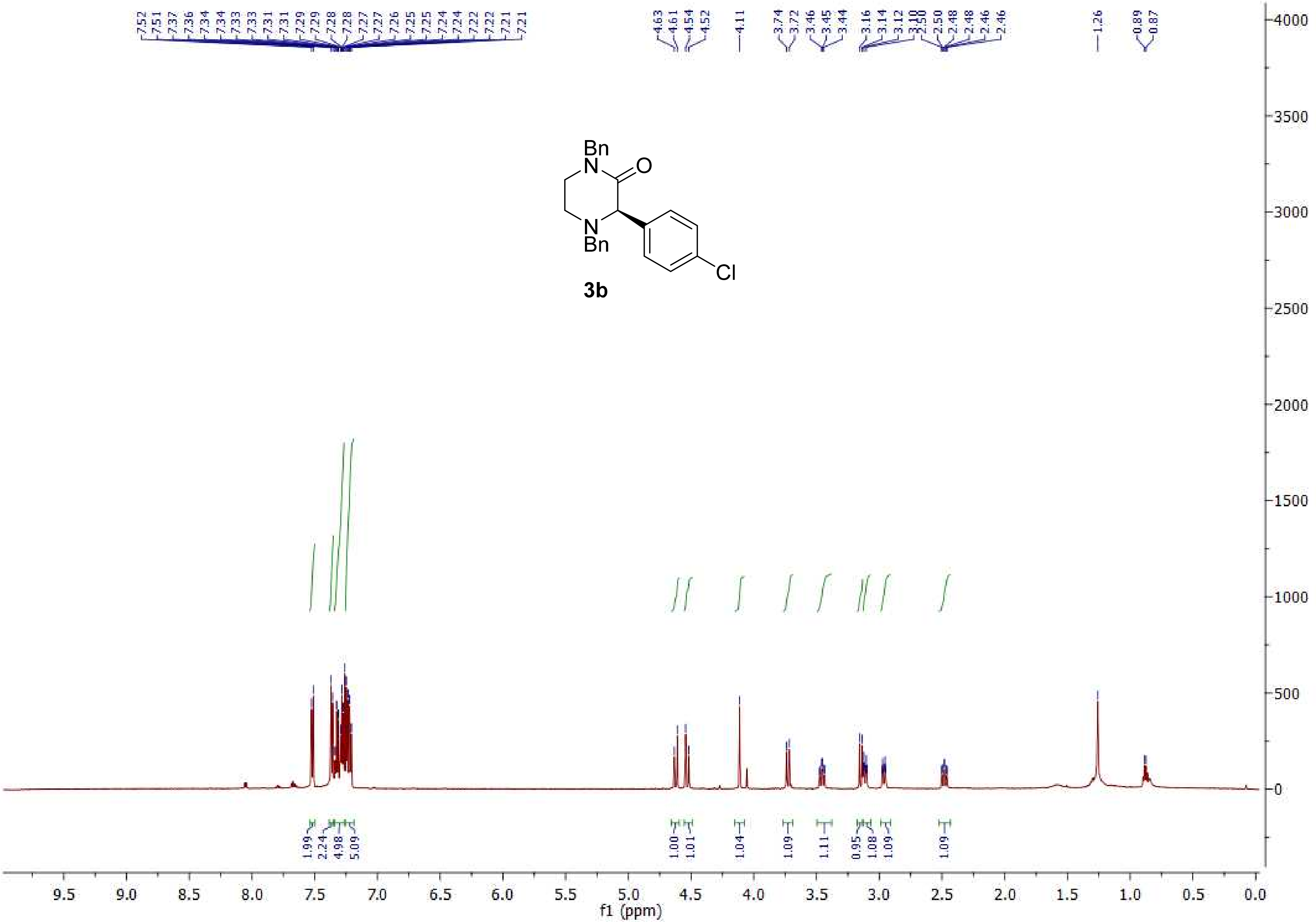

$^{13}$**C{$^{1}$H}** NMR in $CDCl_3$ (150 MHz)

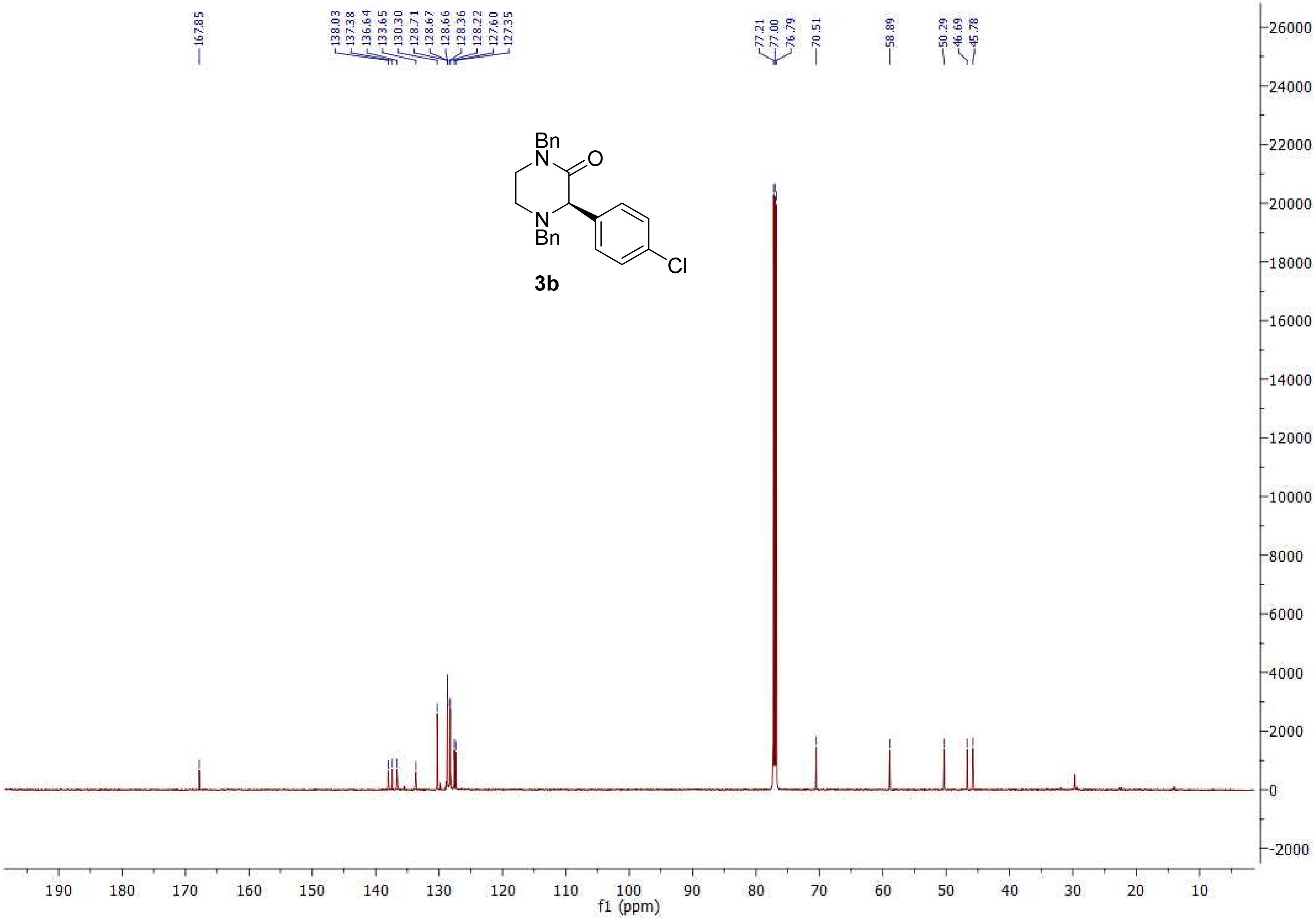

$^{1}$H NMR in $CDCl_3$ (600 MHz)

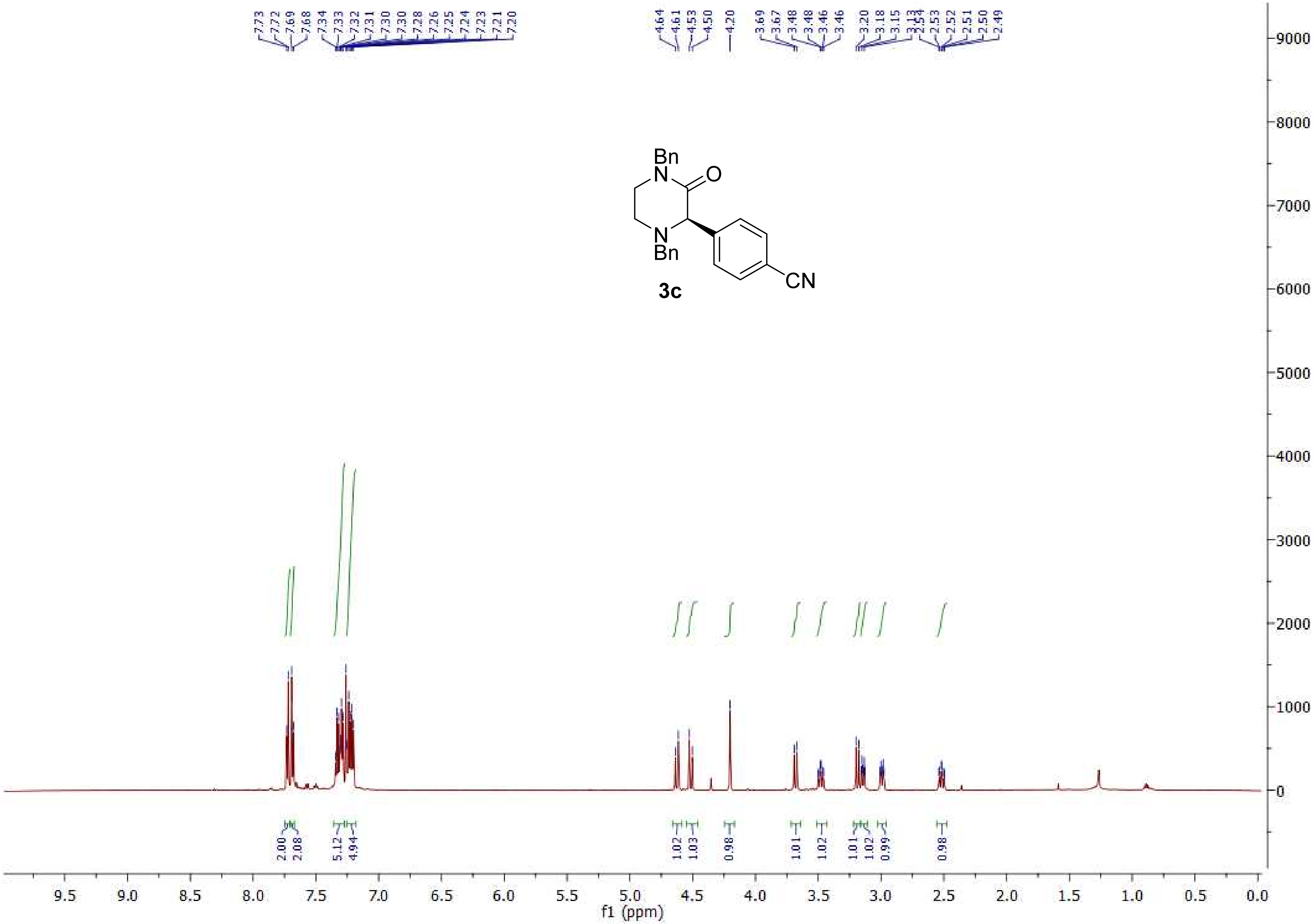

$^{13}$**C{$^{1}$H}** NMR in $CDCl_3$ (150 MHz)

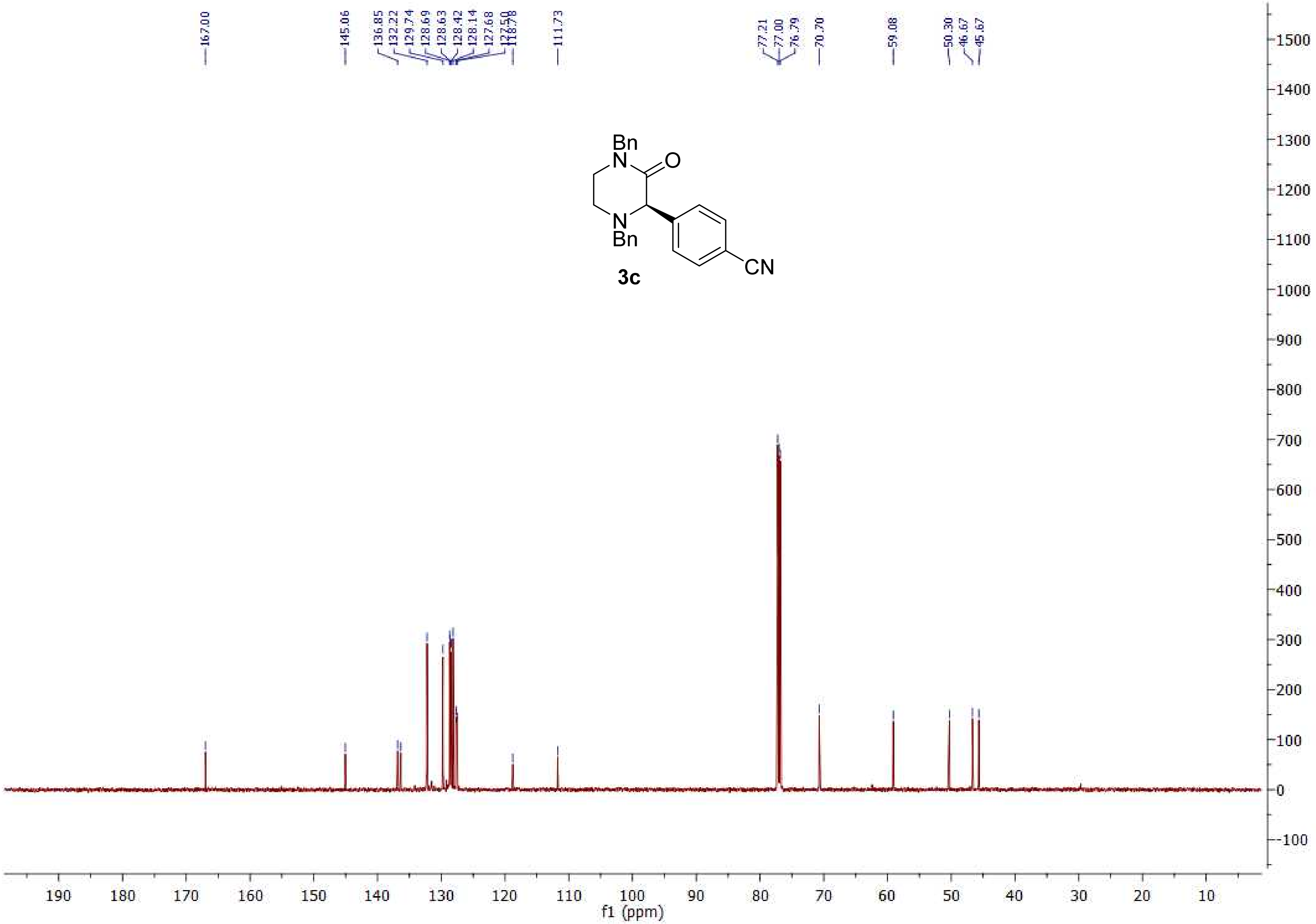

<sup></sup>

$^{1}$H NMR in $CDCl_3$ (600 MHz)

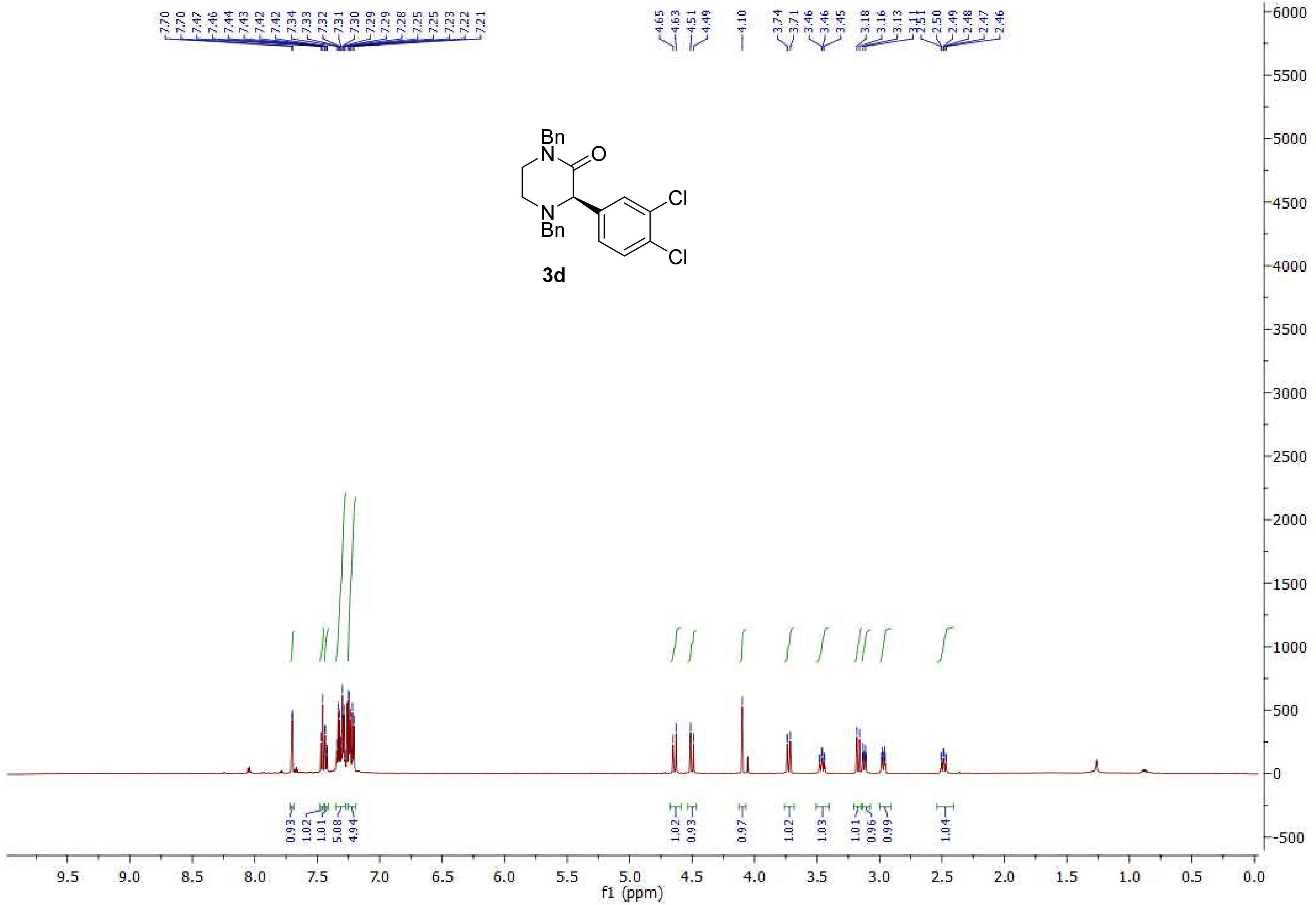

**$^{13}C\{^{1}H\}$** NMR in $CDCl_3$ (150 MHz)

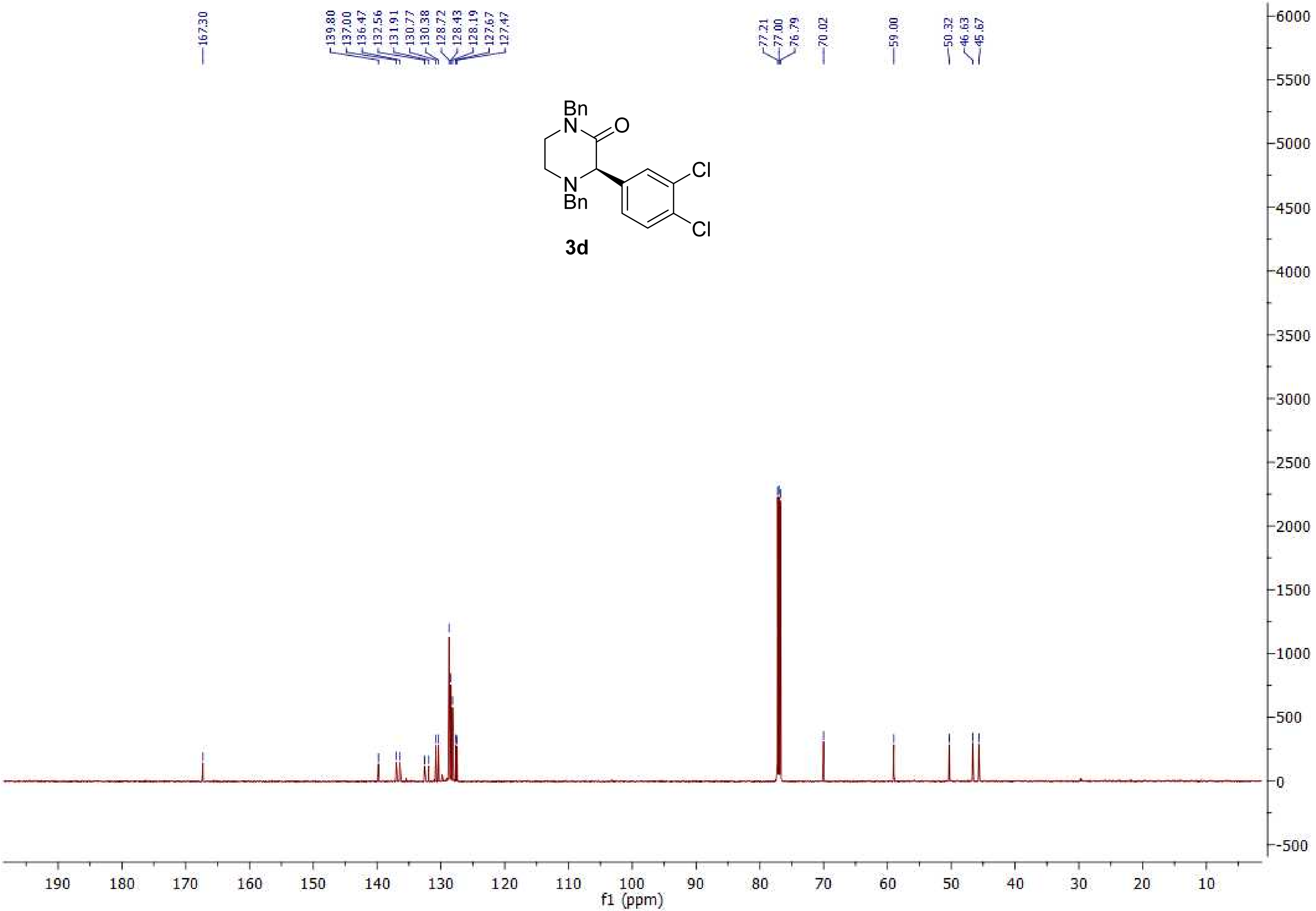

$^1H$ NMR in $CDCl_3$ (600 MHz)

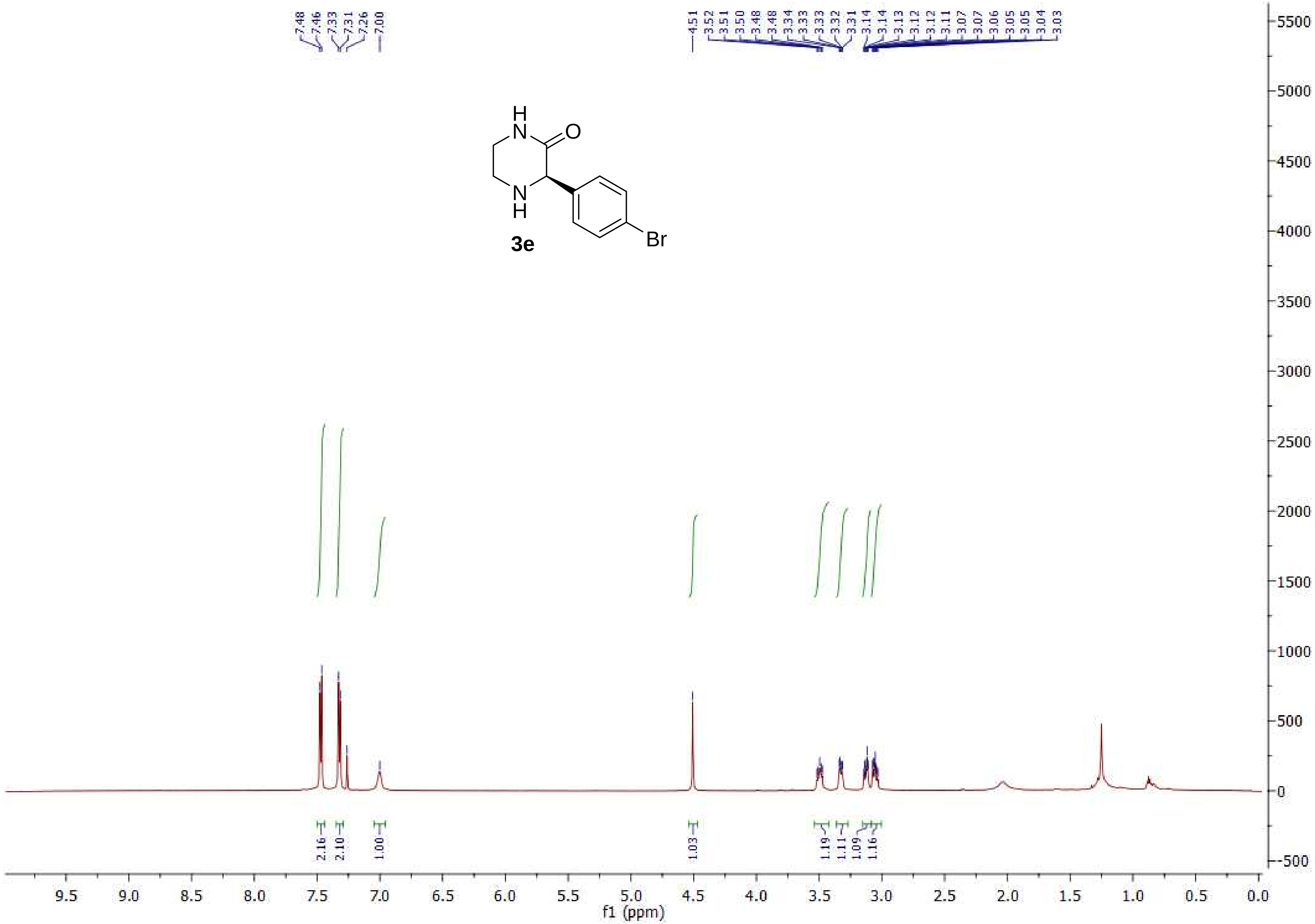

$^{13}$**C{$^{1}$H}** NMR in $CDCl_3$ (150 MHz)

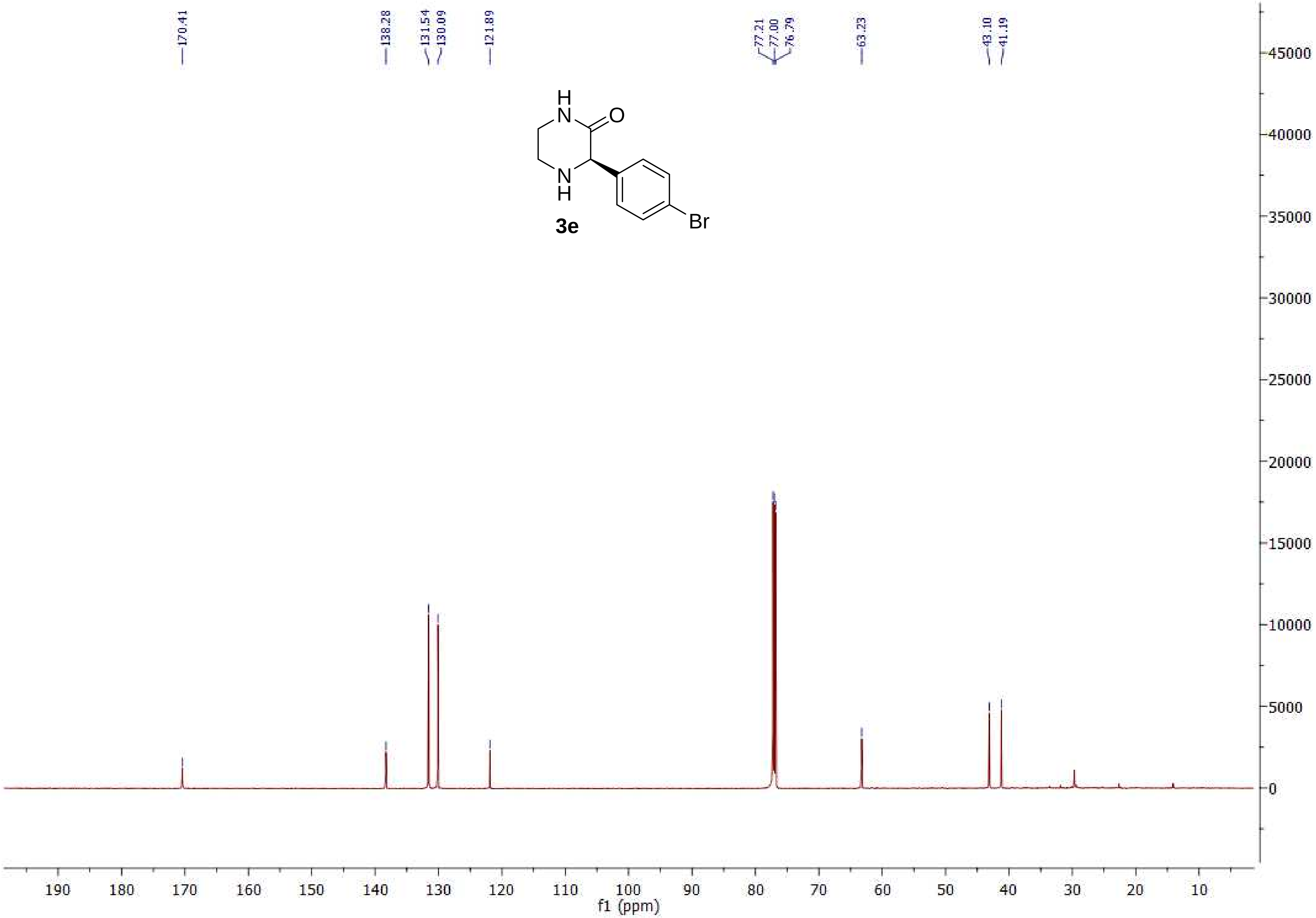

$^{1}$H NMR in $CDCl_3$ (600 MHz)

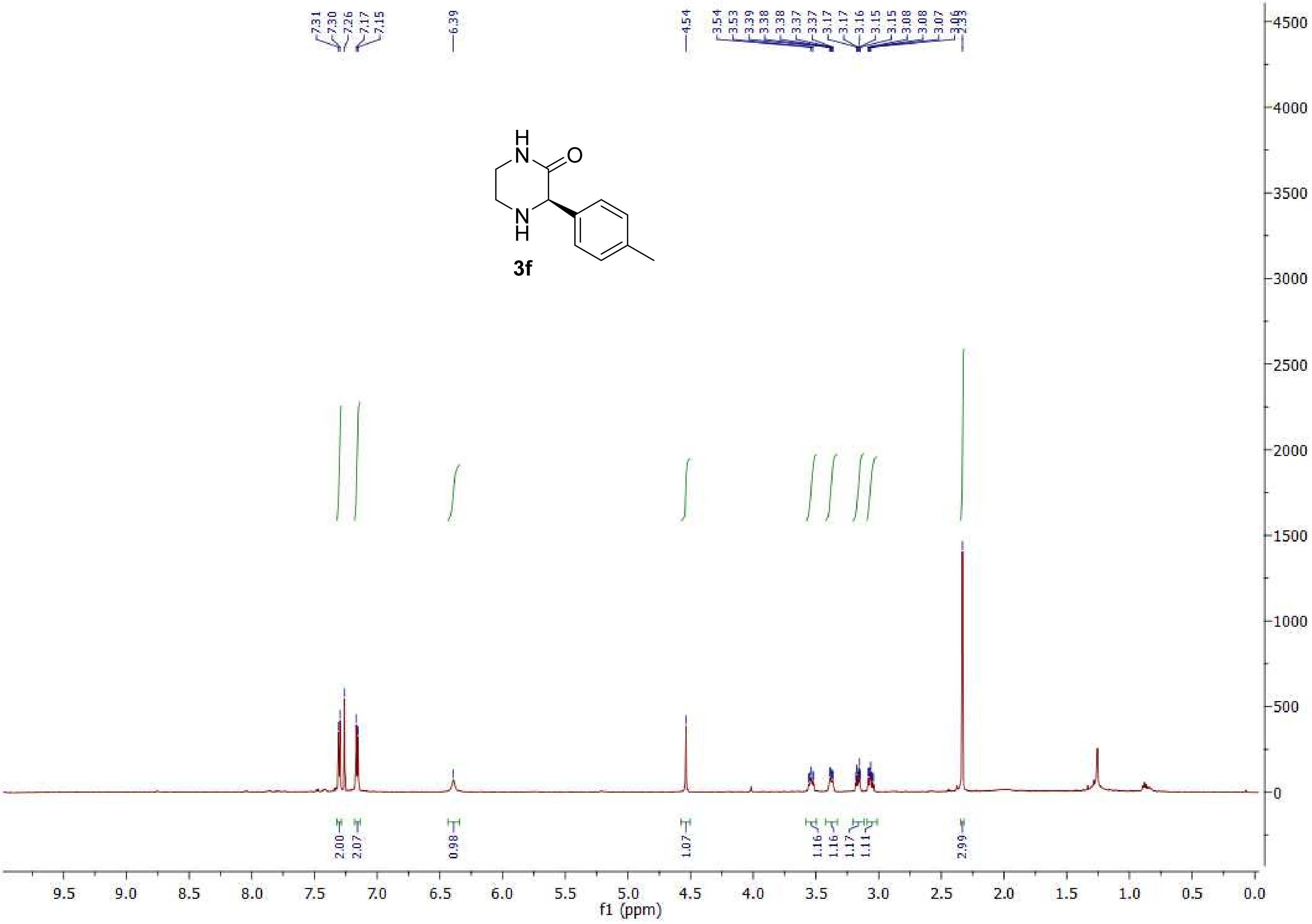

$^{13}$**C{$^{1}$H}** NMR in $CDCl_3$ (150 MHz)

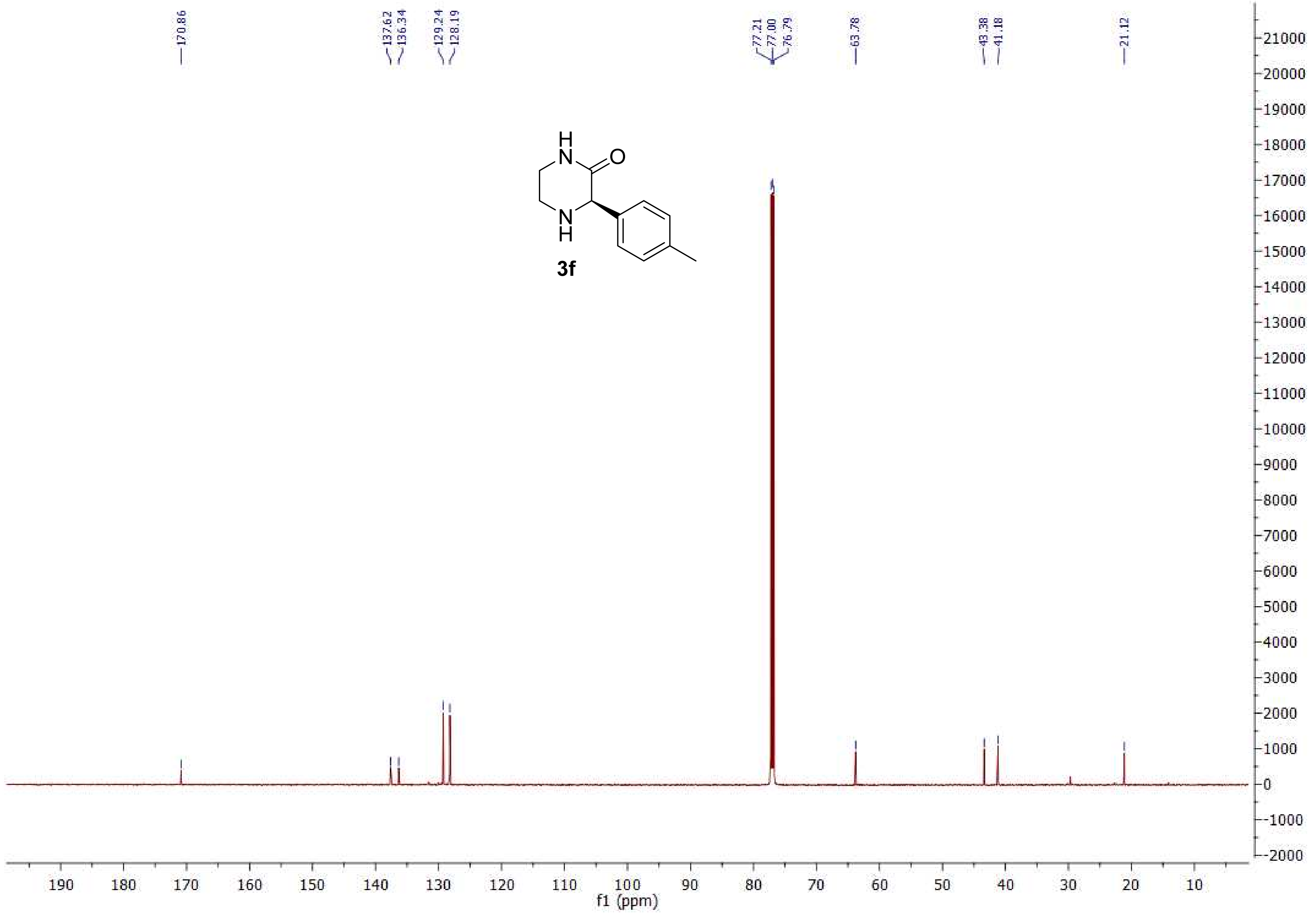

$^{1}H$ NMR in $CDCl_3$ (600 MHz)

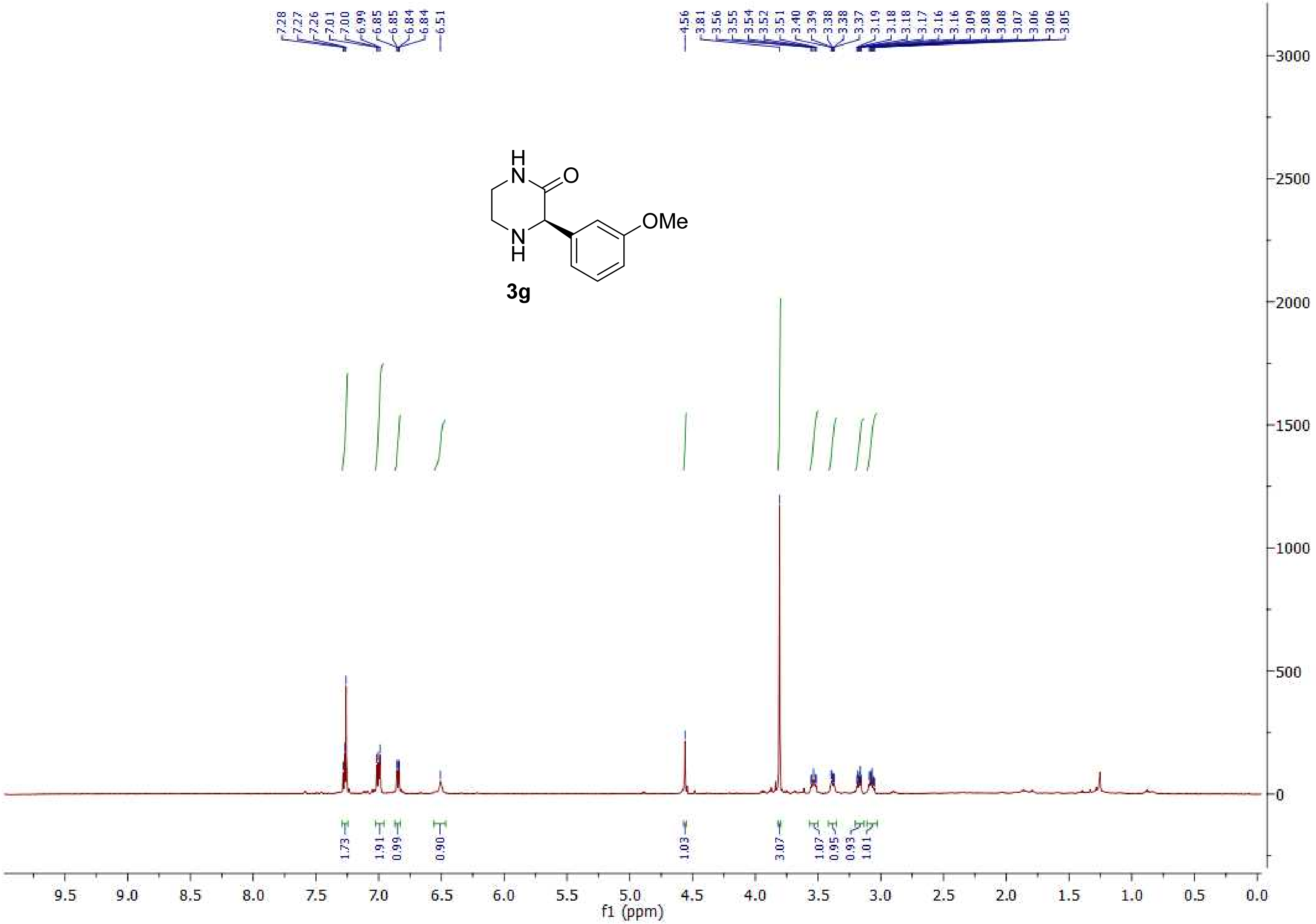

**$^{13}$C{$^{1}$H}** NMR in $CDCl_3$ (150 MHz)

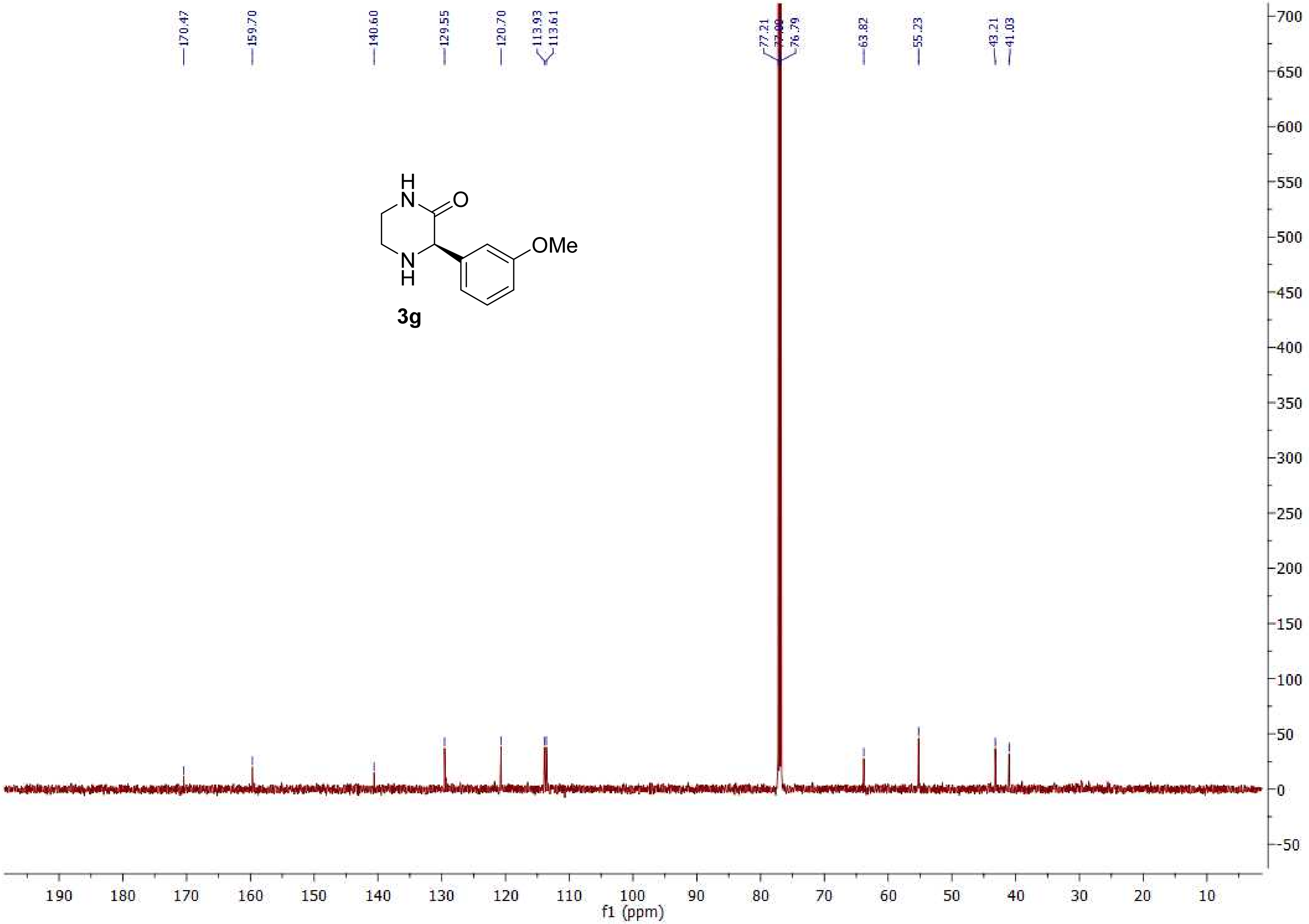

$^{1}$H NMR in $CDCl_3$ (600 MHz)

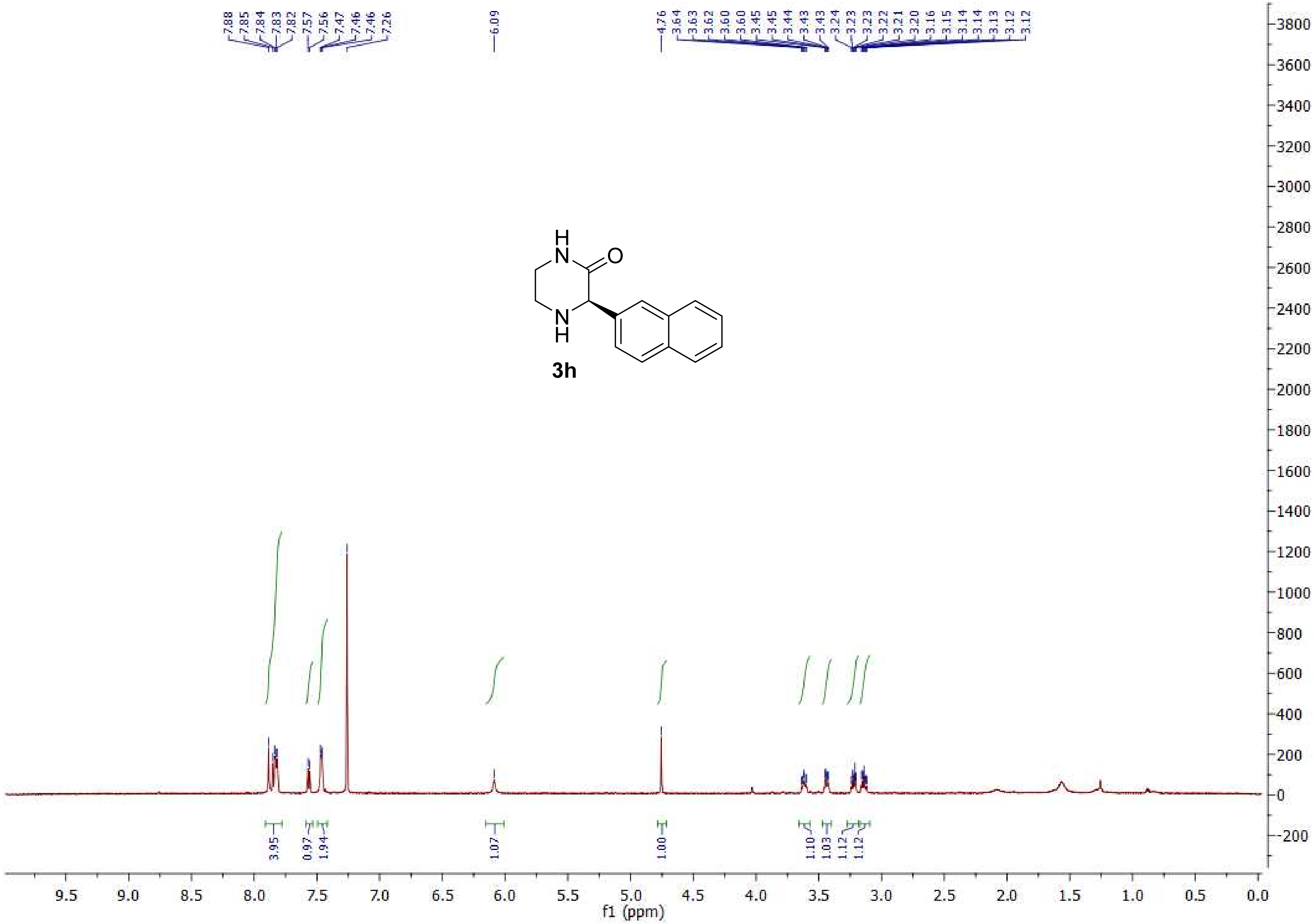


3h

**$^{13}$C{$^{1}$H}** NMR in $CDCl_3$ (150 MHz)

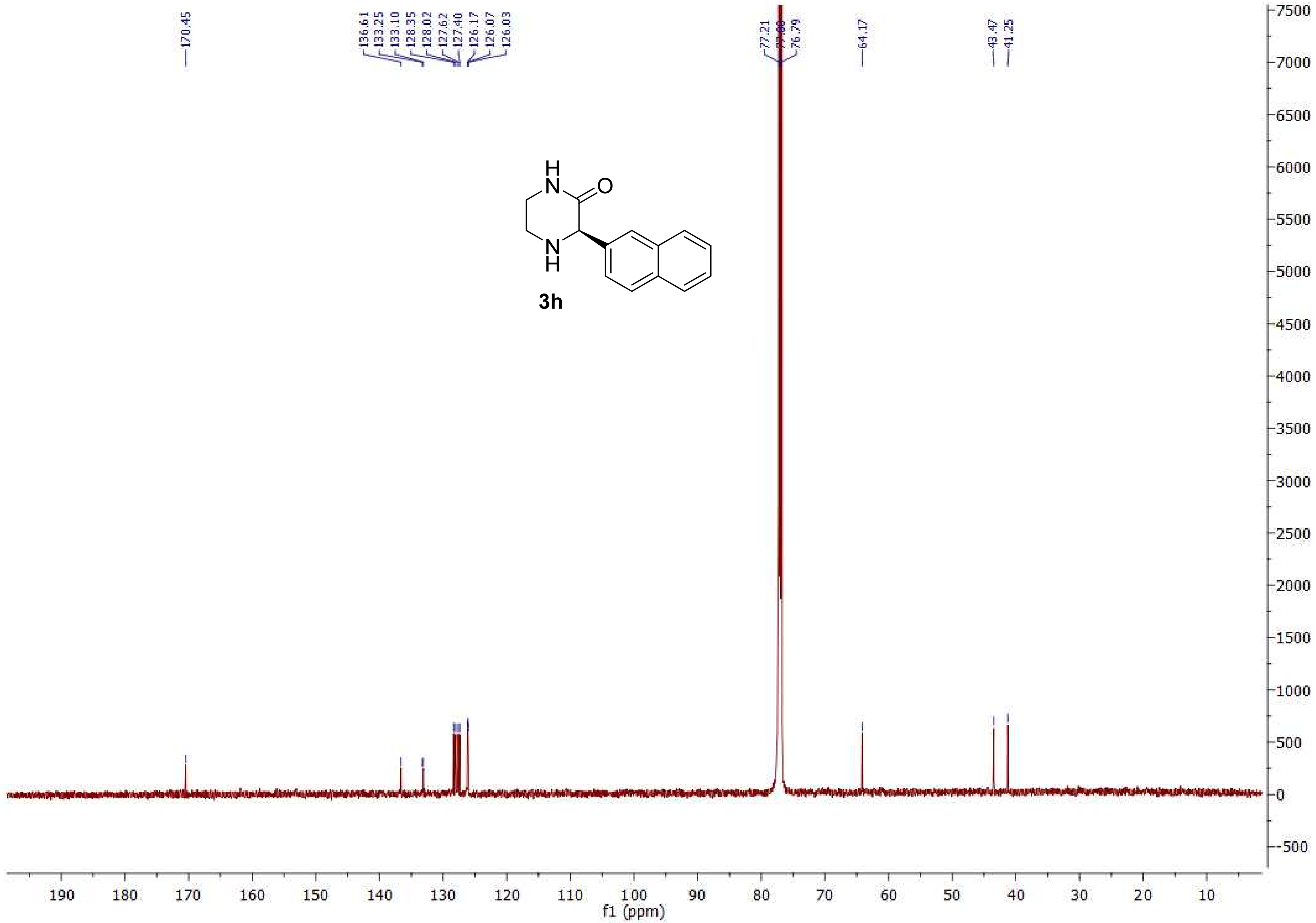

$^{1}H$ NMR in $CDCl_3$ (600 MHz)

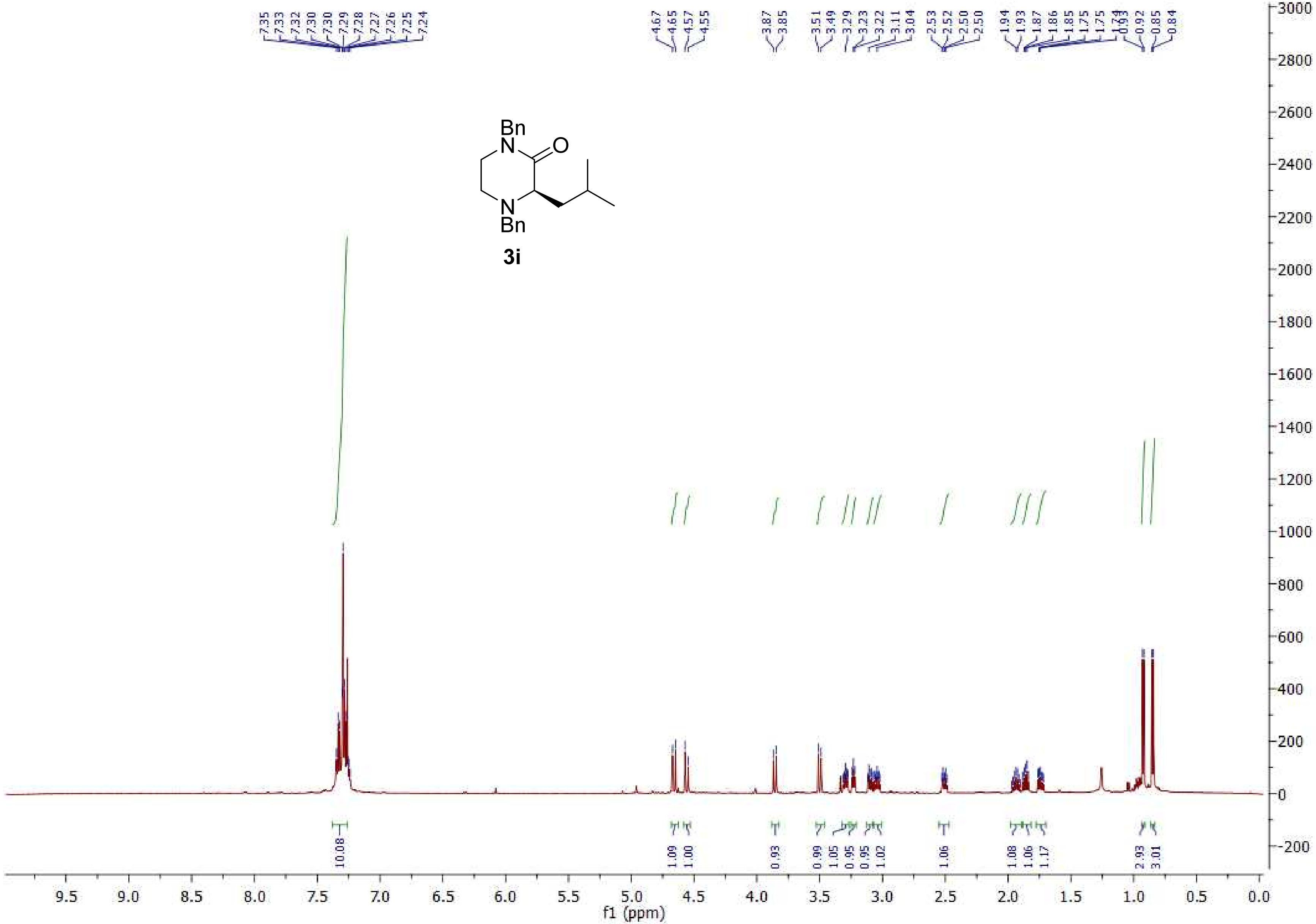

$^{13}$**C{$^{1}$H}** NMR in $CDCl_3$ (150 MHz)

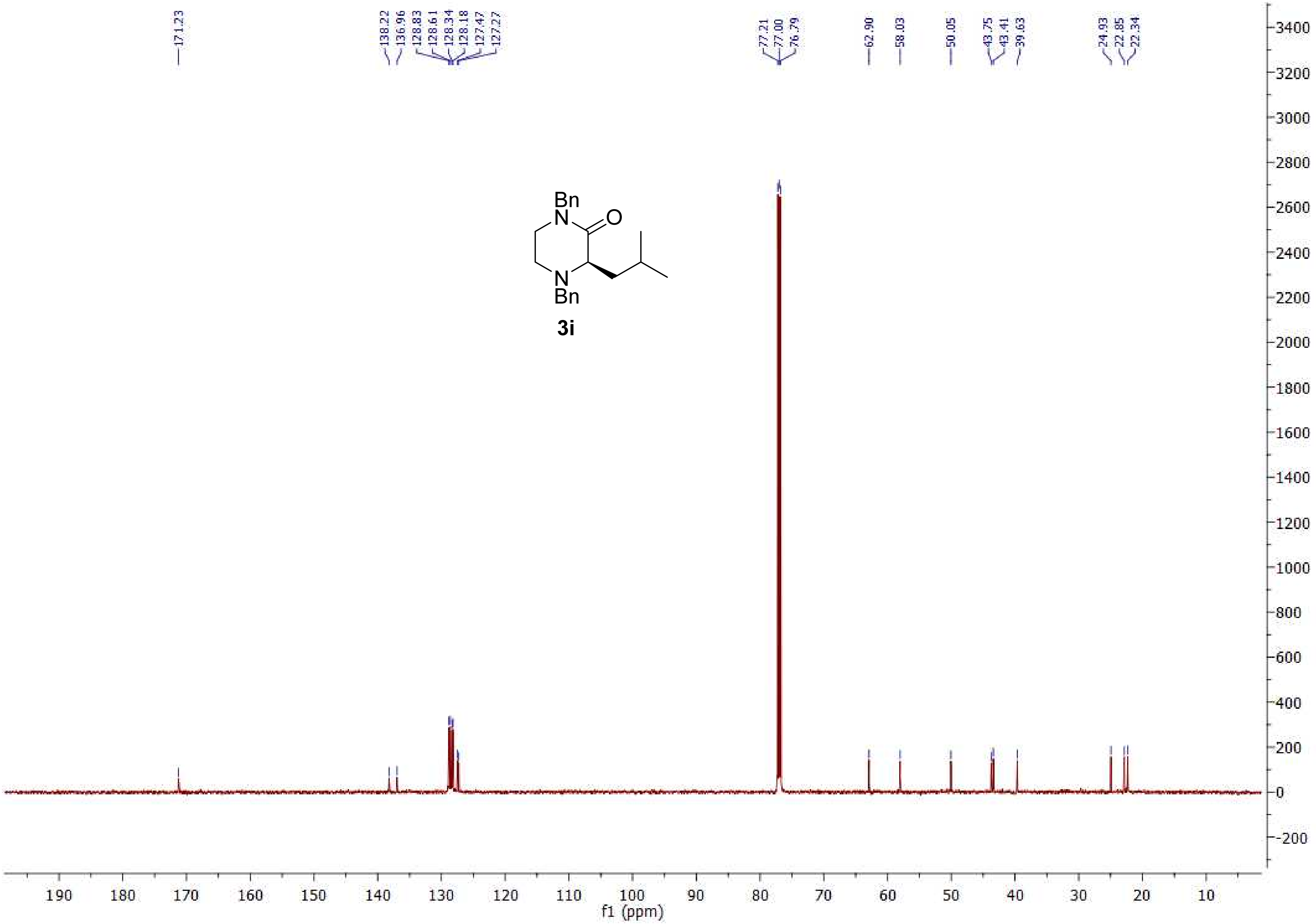

$^{1}$H NMR in $CDCl_3$ (300 MHz)

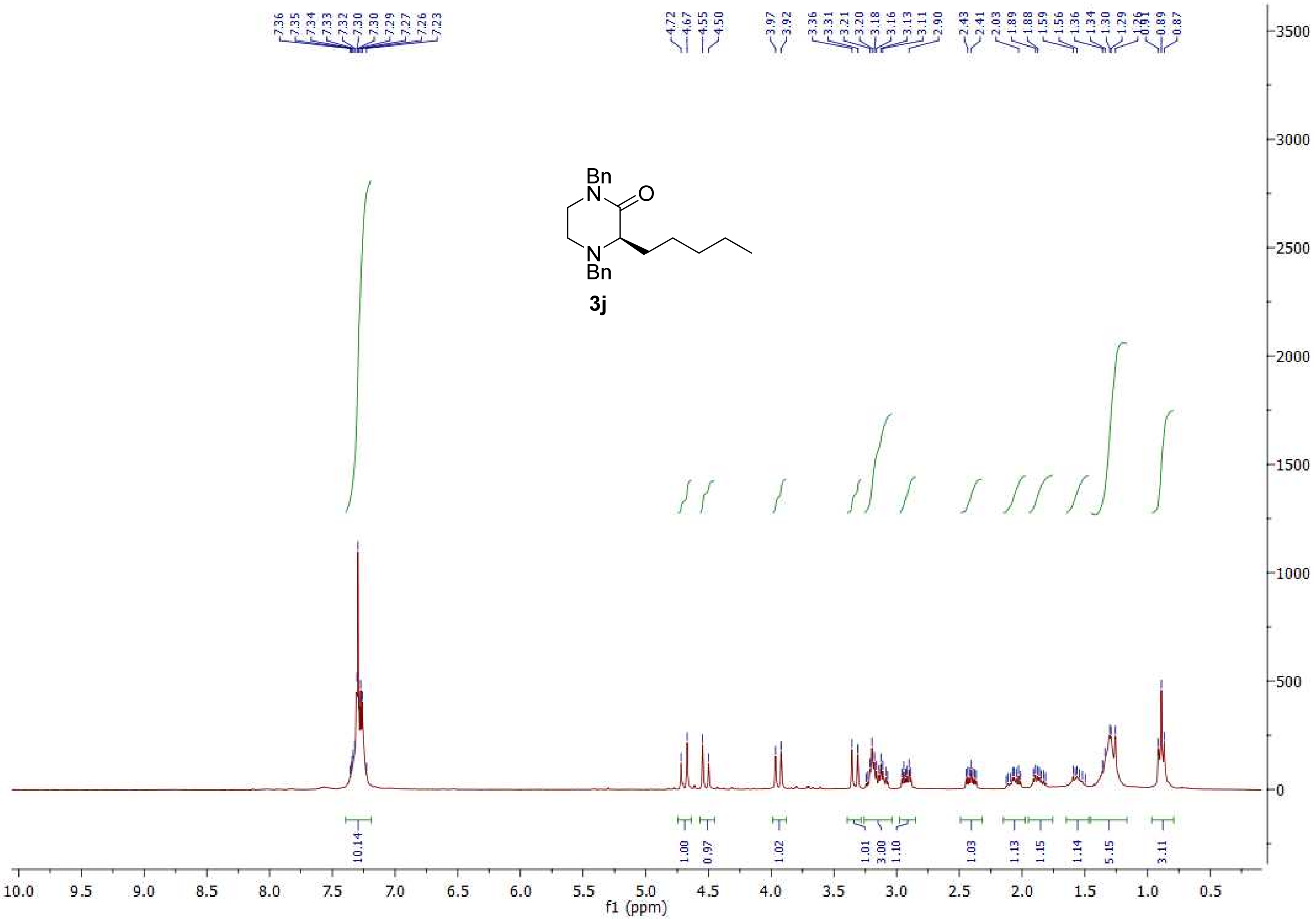

**$^{13}$C{$^{1}$H}** NMR in $CDCl_3$ (75 MHz)

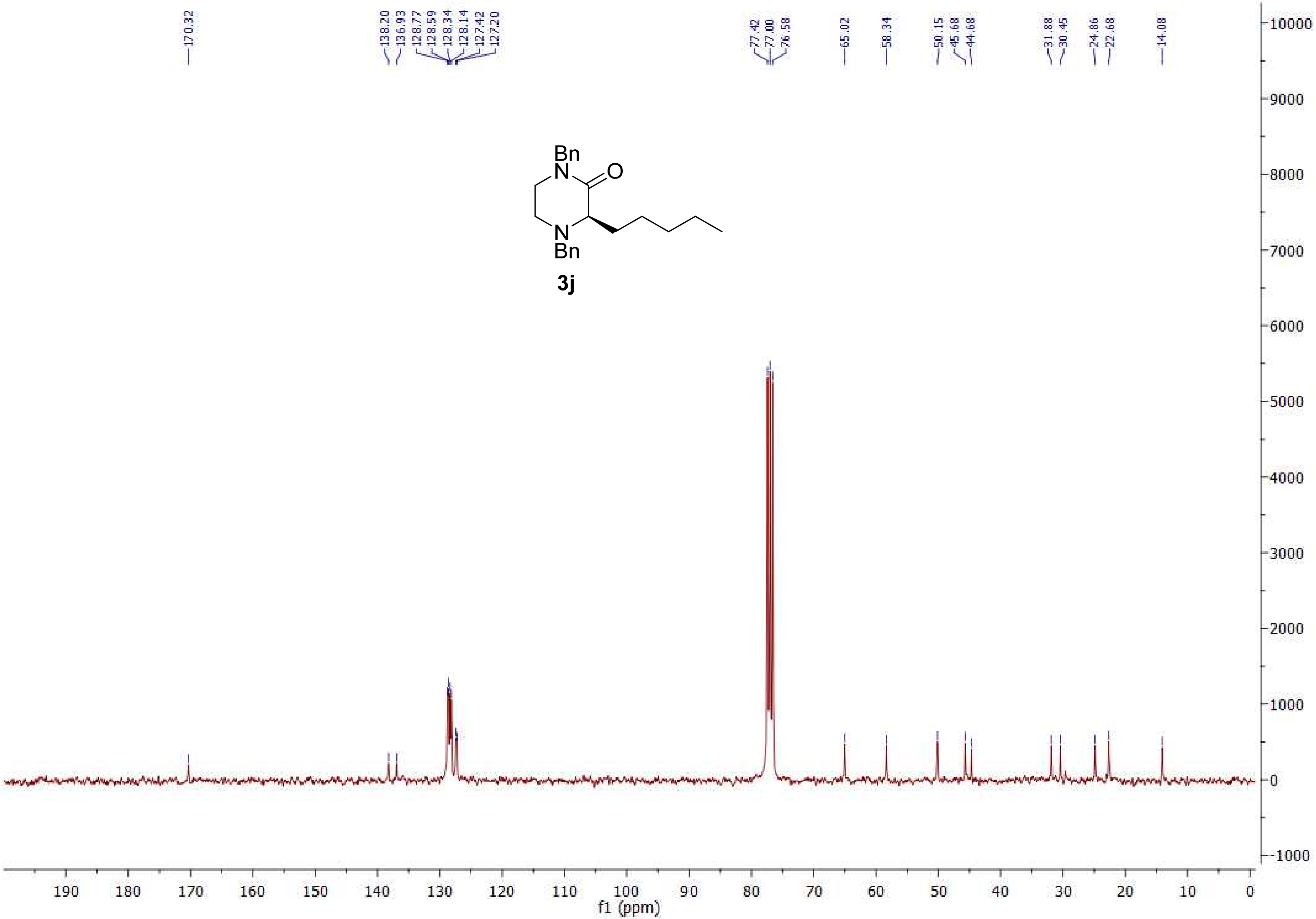


**3j**

$^1$H NMR in $CDCl_3$ (600 MHz)

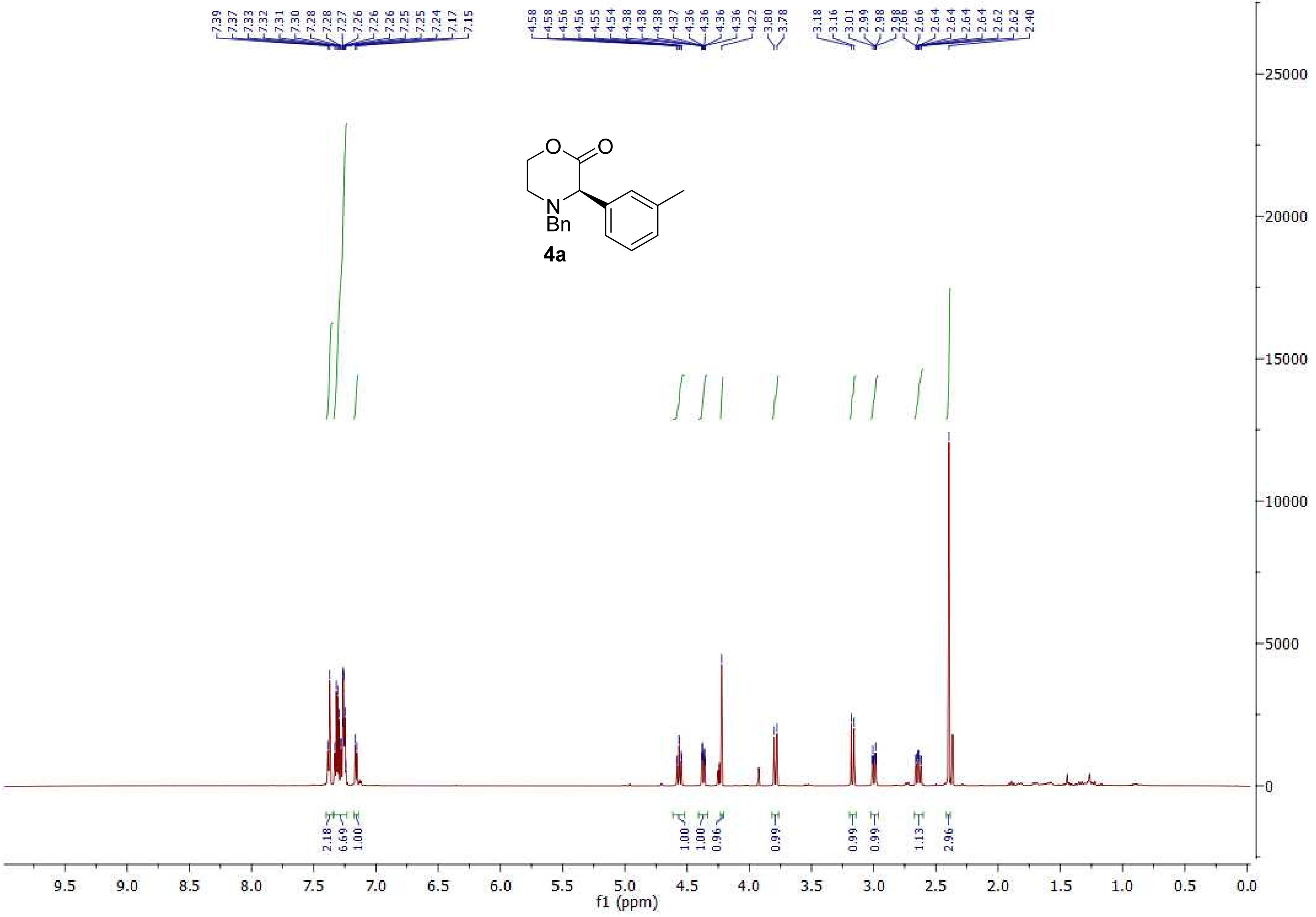

**$^{13}$C{$^{1}$H}** NMR in $CDCl_3$ (150 MHz)

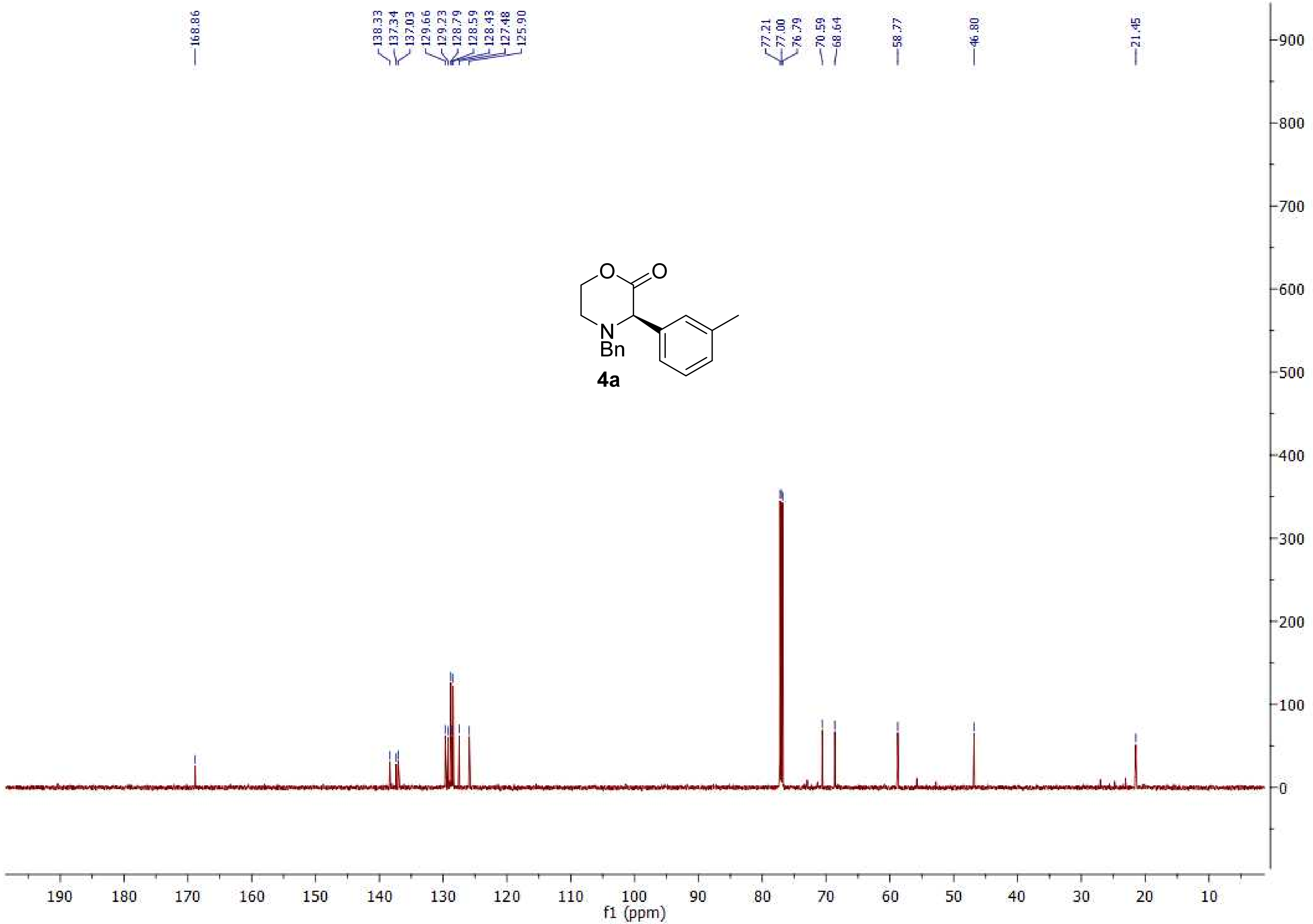

$^{1}$H NMR in $CDCl_3$ (400 MHz)

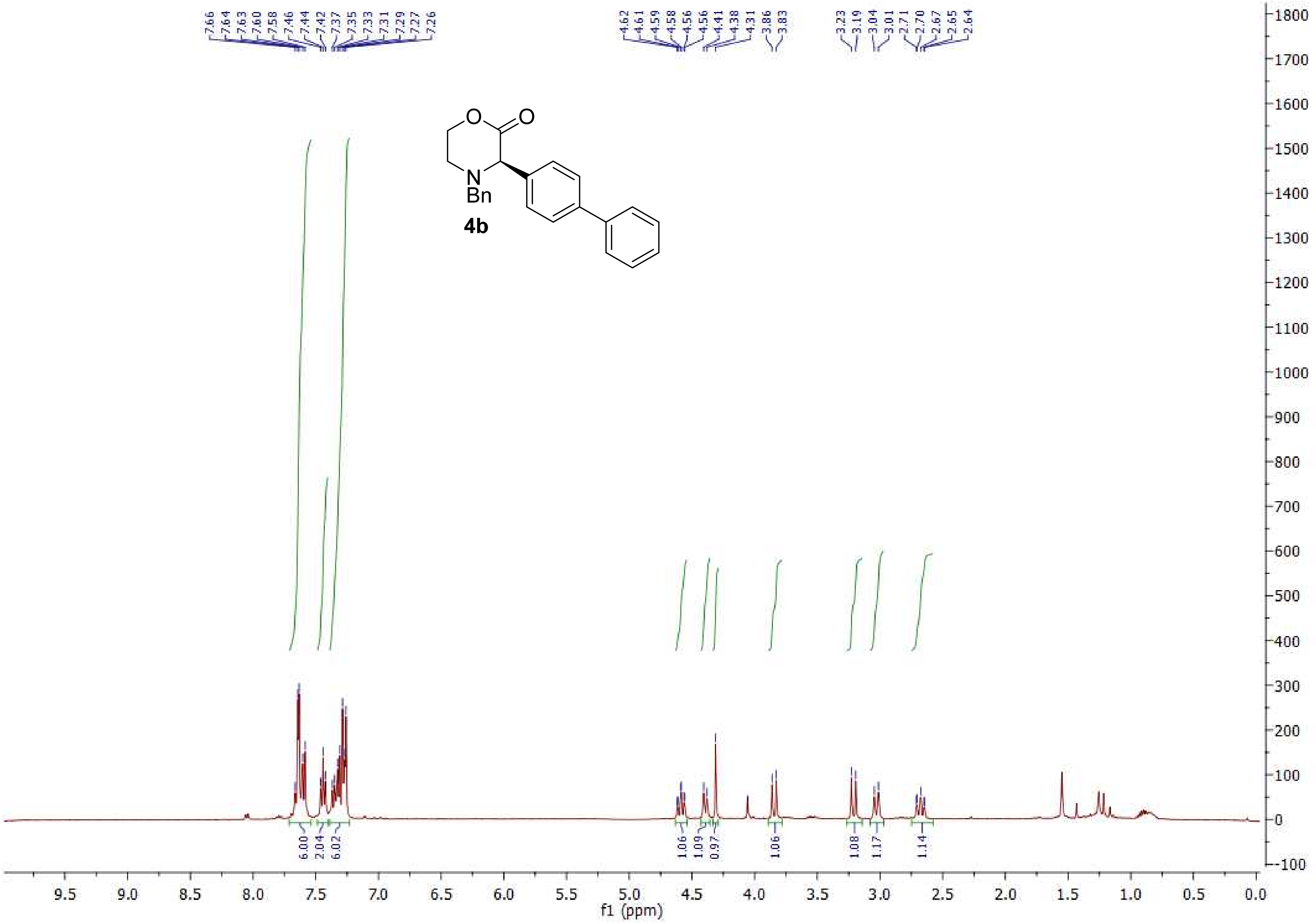

**$^{13}$C{$^{1}$H}** NMR in $CDCl_3$ (100 MHz)

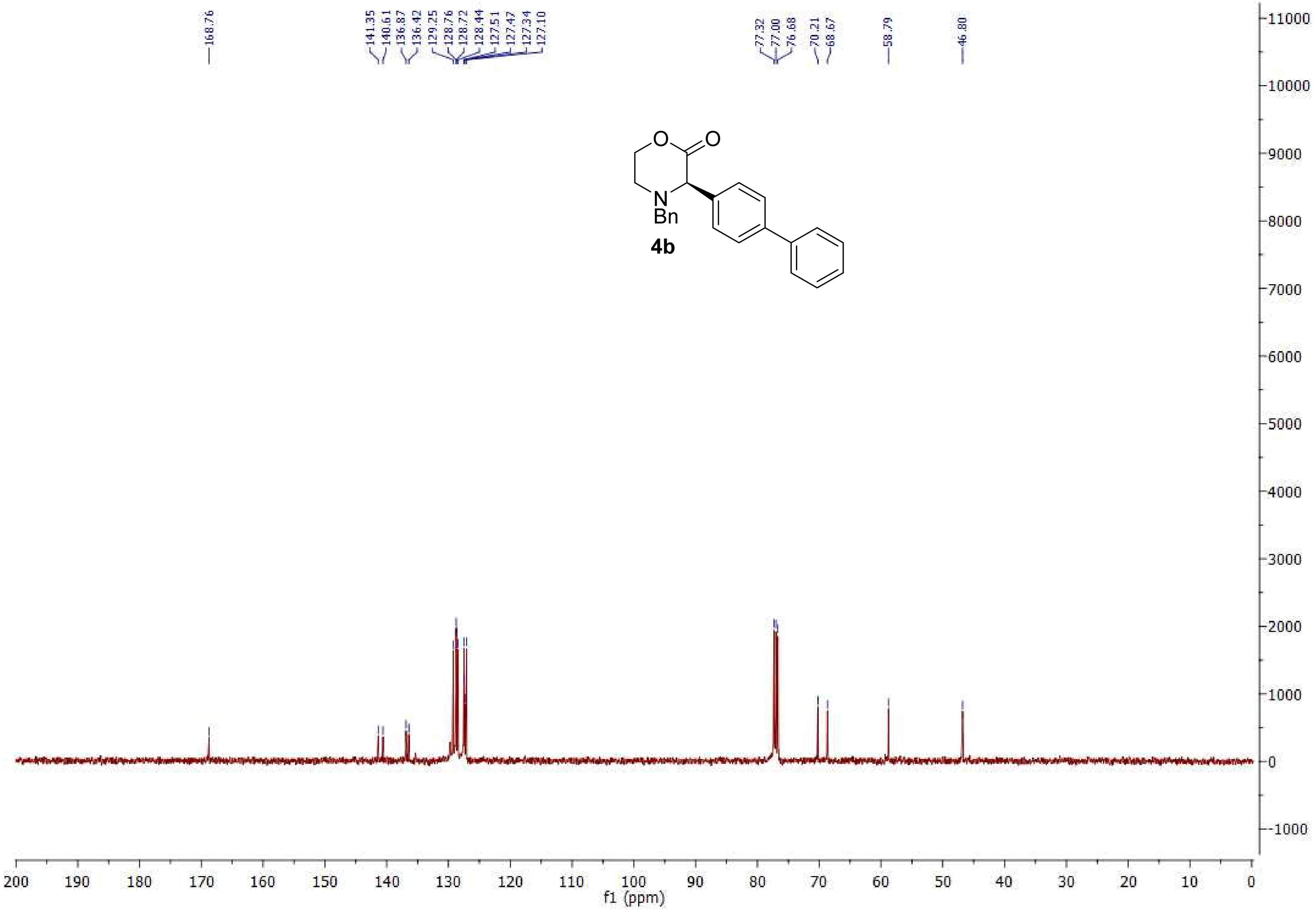

$^{1}H$ NMR in $CDCl_3$ (600 MHz)

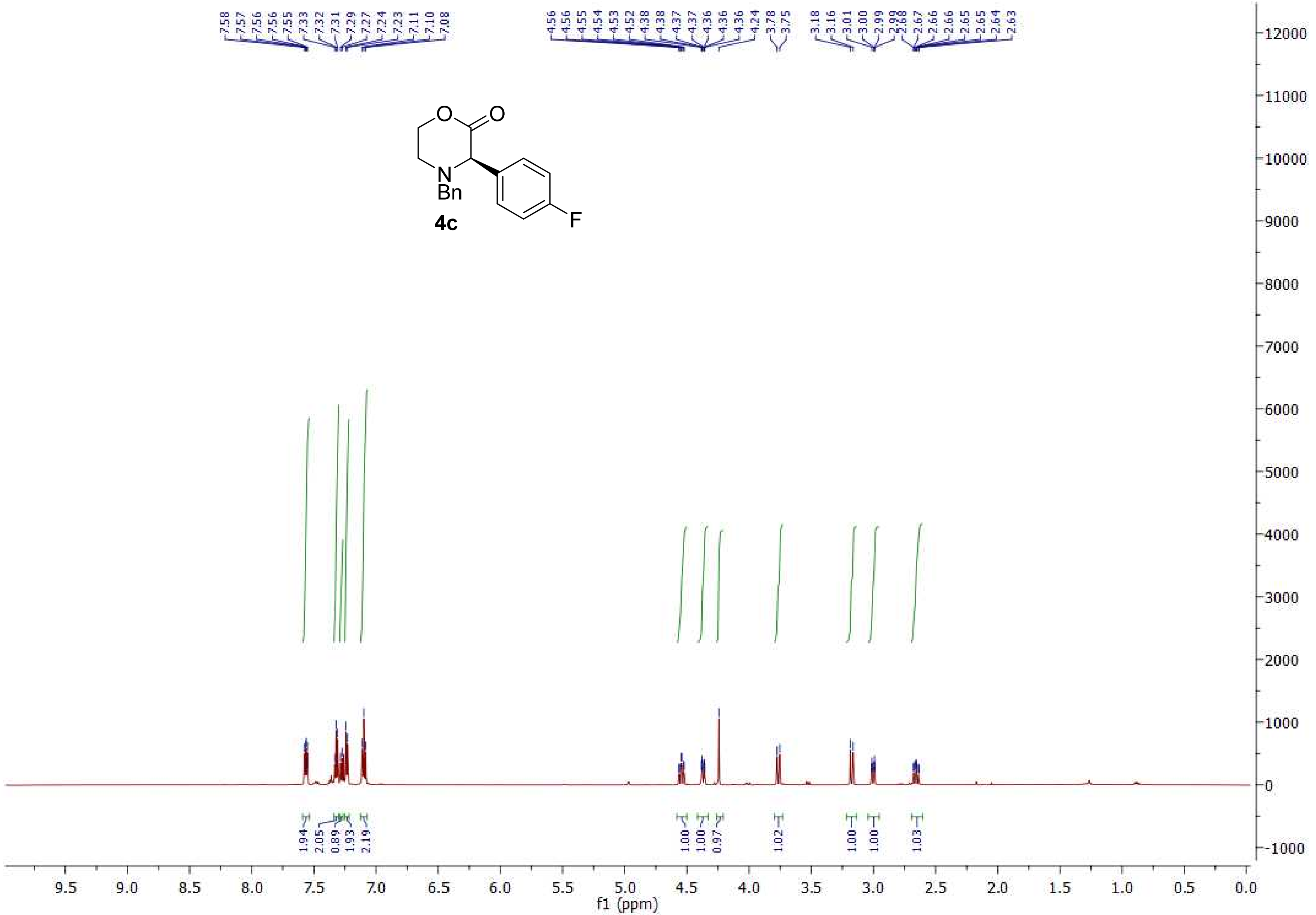

**$^{13}C\{^1H\}$** NMR in $CDCl_3$ (150 MHz)

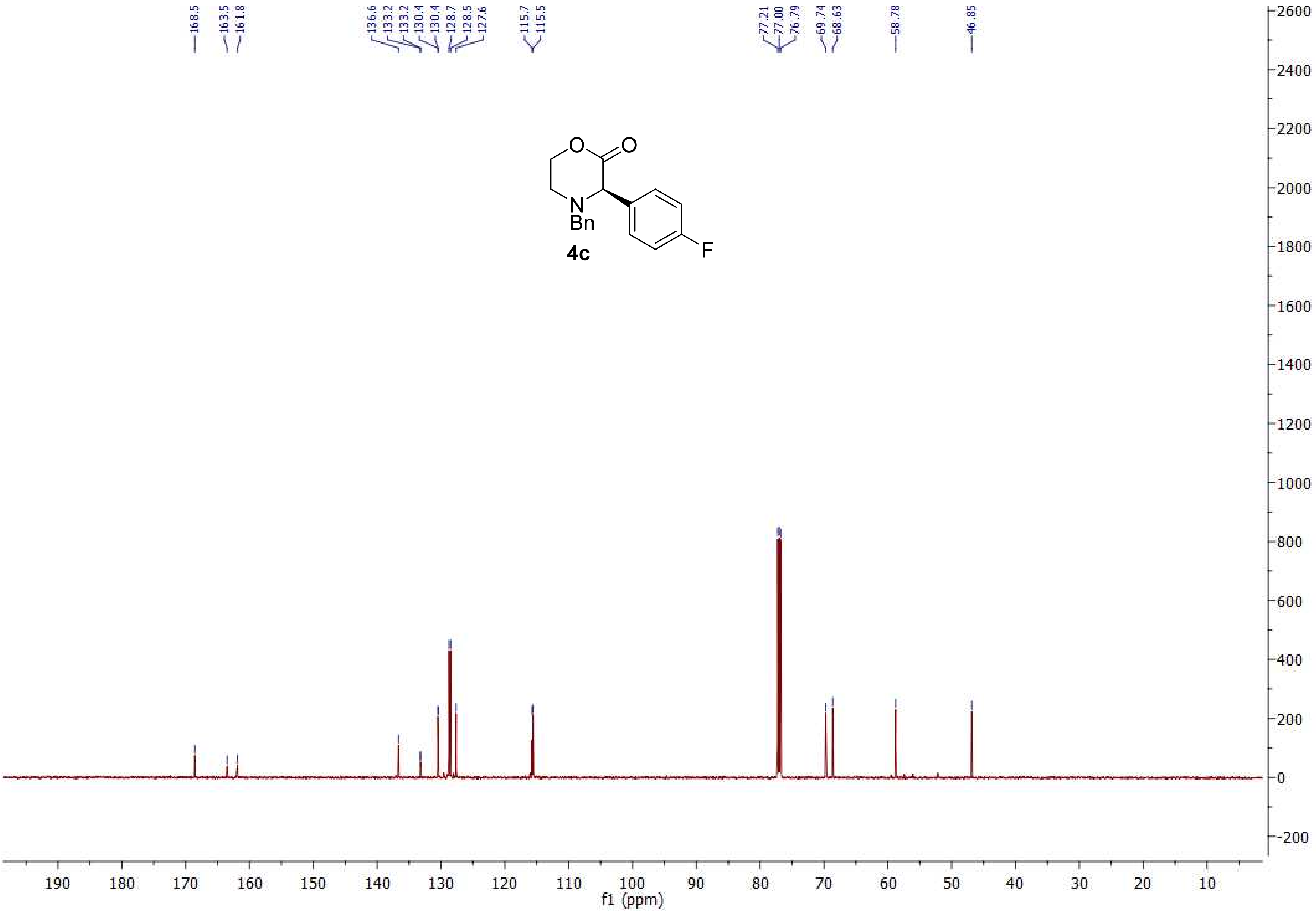

$^{1}$H NMR in $CDCl_3$ (600 MHz)

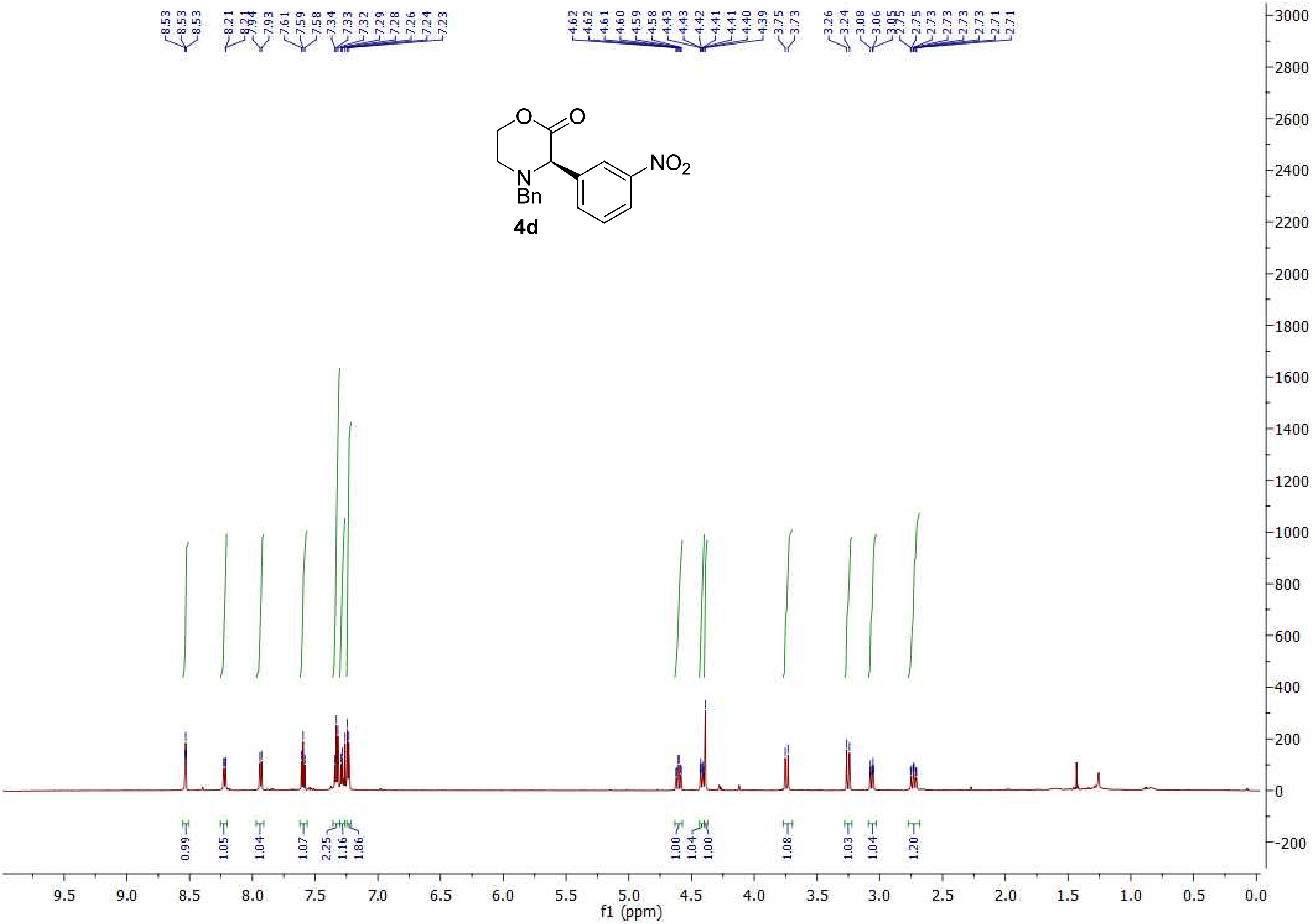

**$^{13}$C{$^{1}$H}** NMR in $CDCl_3$ (150 MHz)

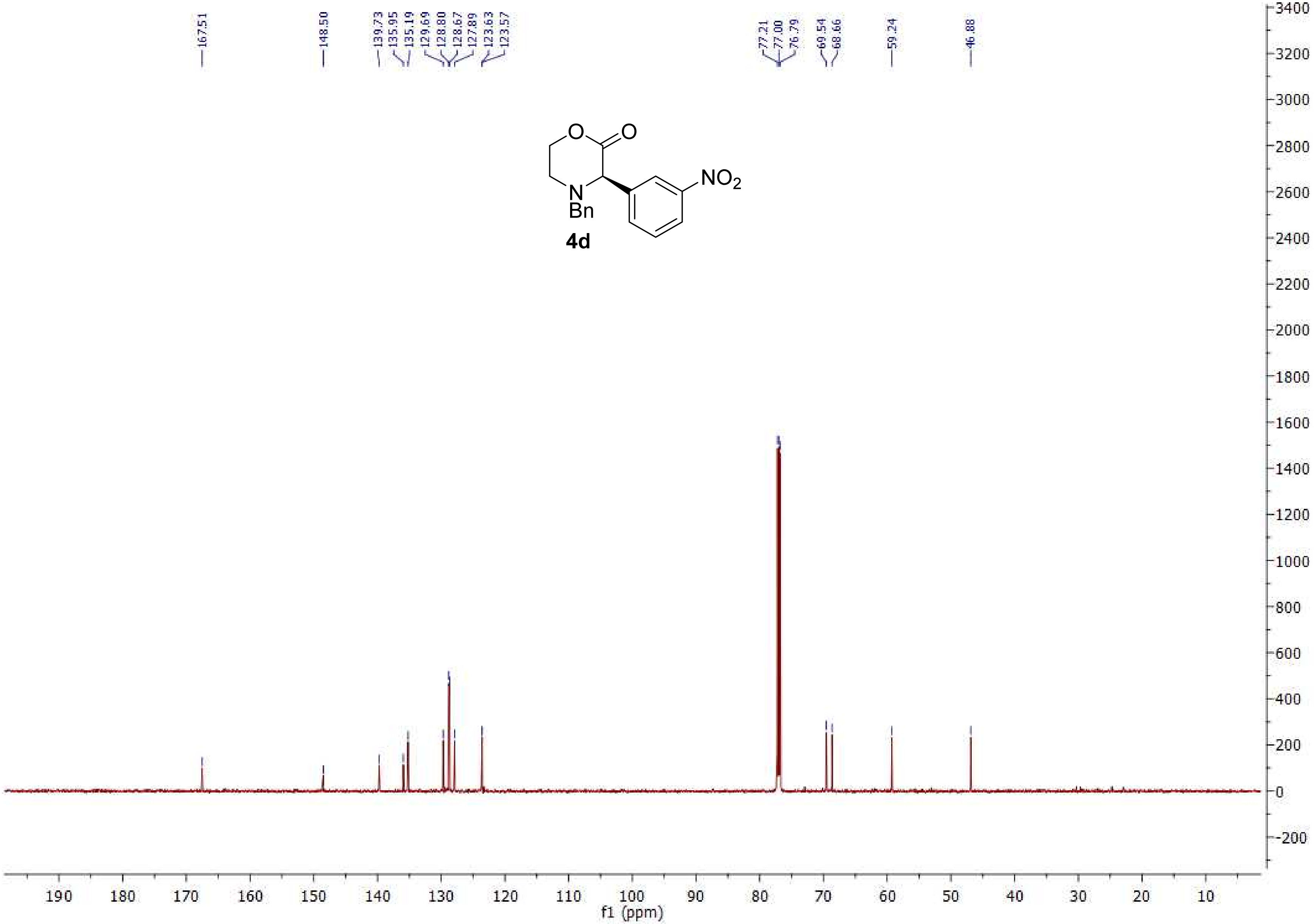

$^{1}H$ NMR in $CDCl_3$ (600 MHz)

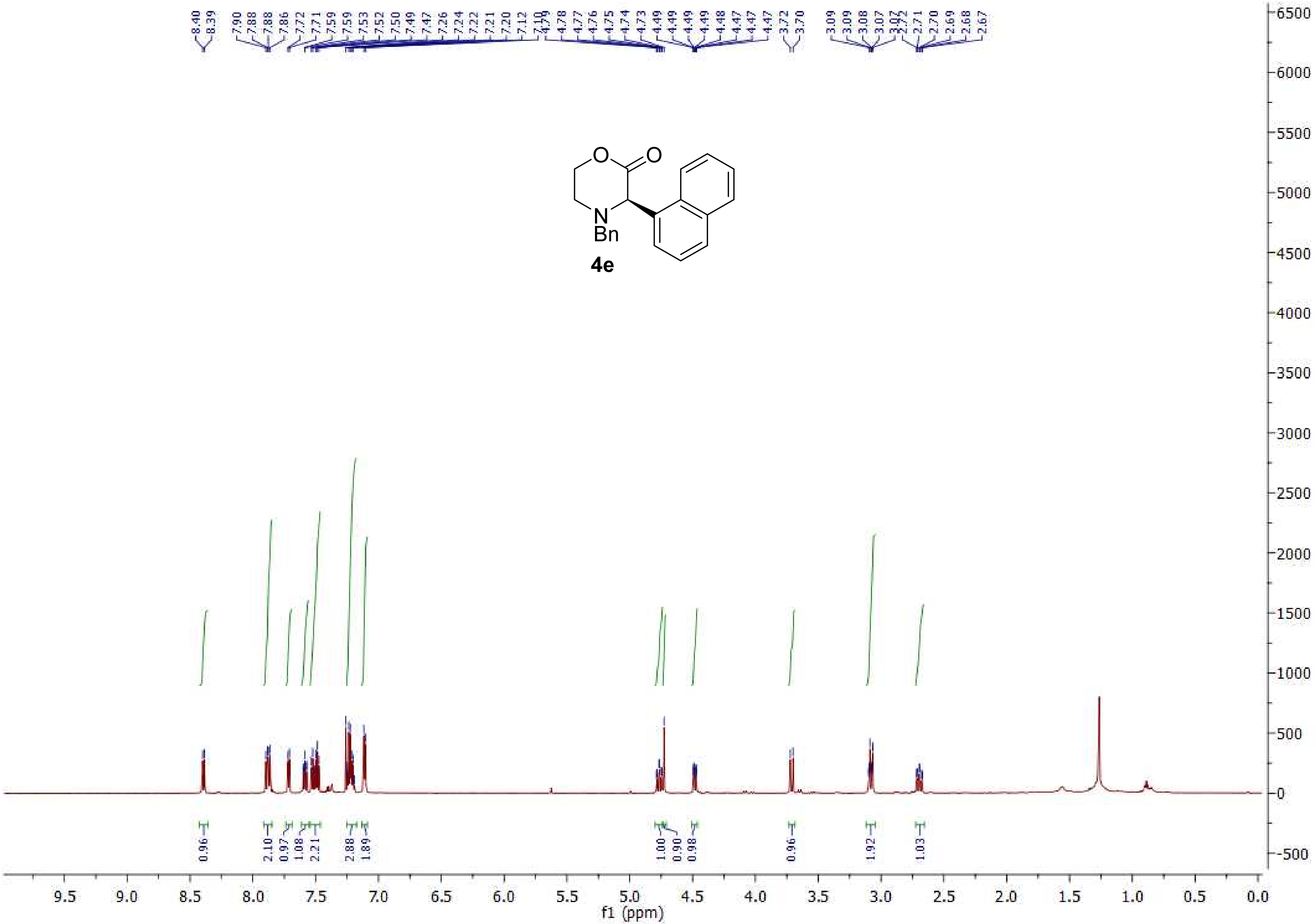

**$^{13}$C{$^{1}$H}** NMR in $CDCl_3$ (150 MHz)

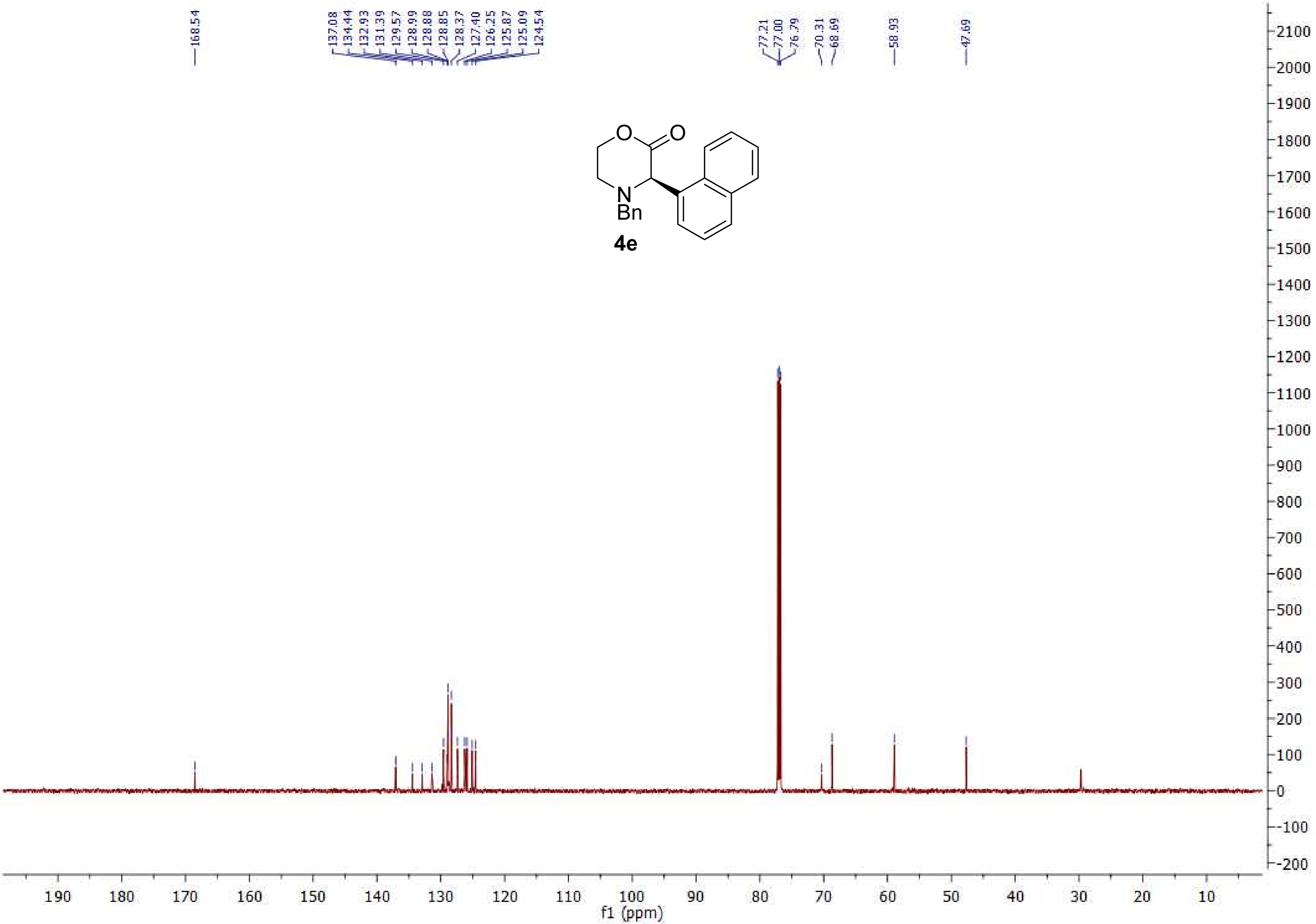

$^{1}H$ NMR in $CDCl_3$ (600 MHz)

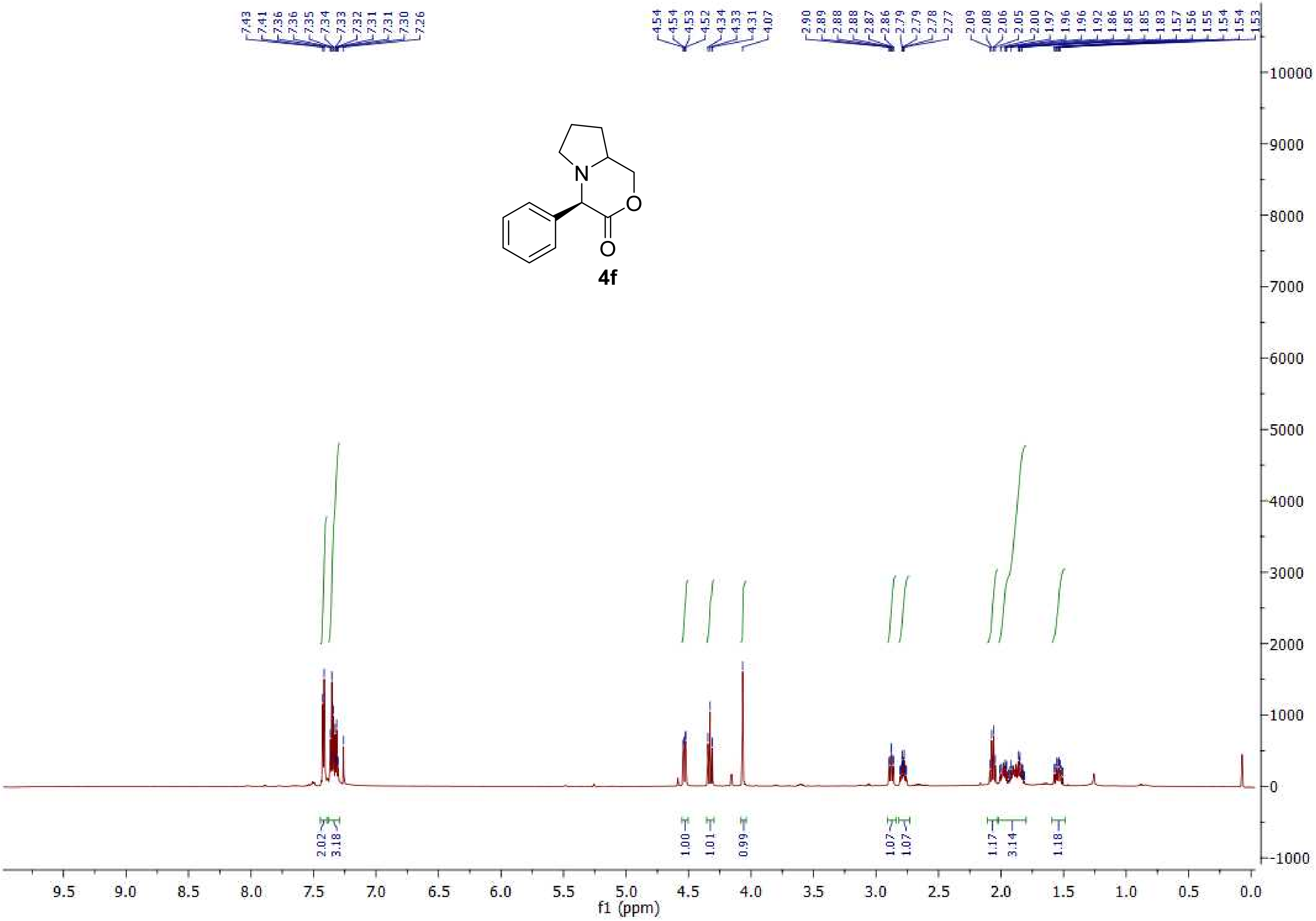

**$^{13}C\{^{1}H\}$** NMR in $CDCl_3$ (150 MHz)

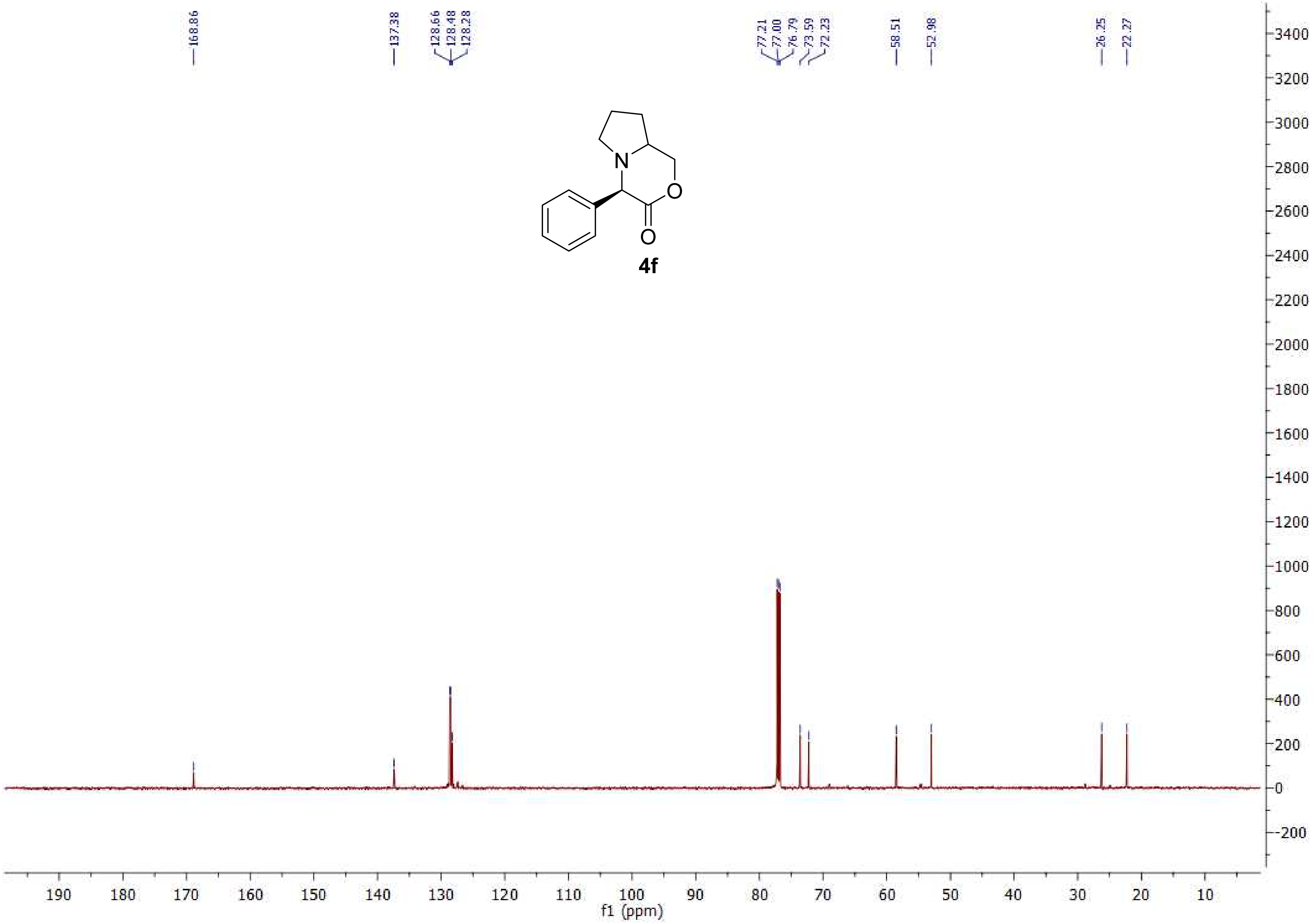

$^{1}H$ NMR in $CDCl_3$ (600 MHz)

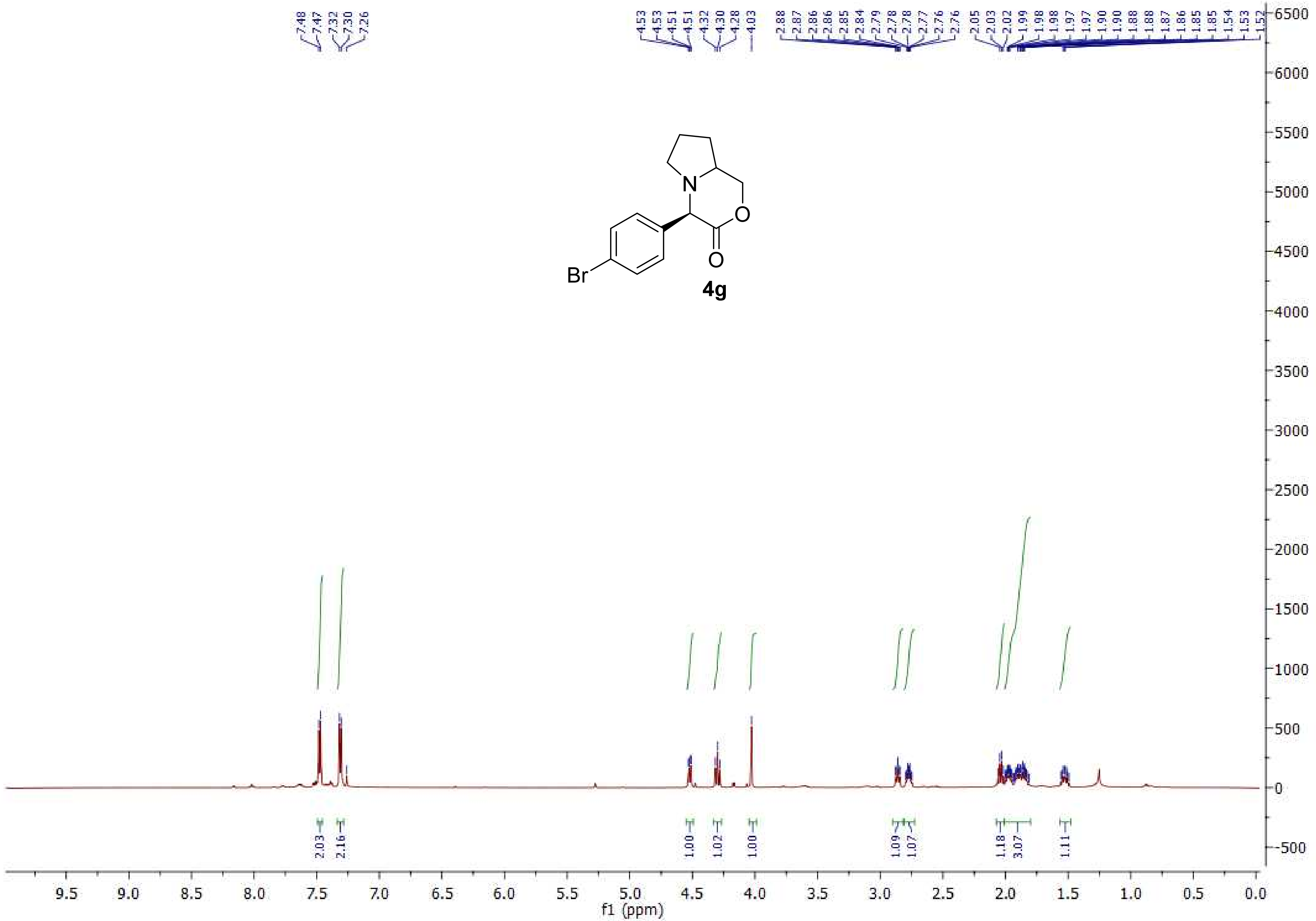

$^{13}$**C{$^{1}$H}** NMR in $CDCl_3$ (150 MHz)

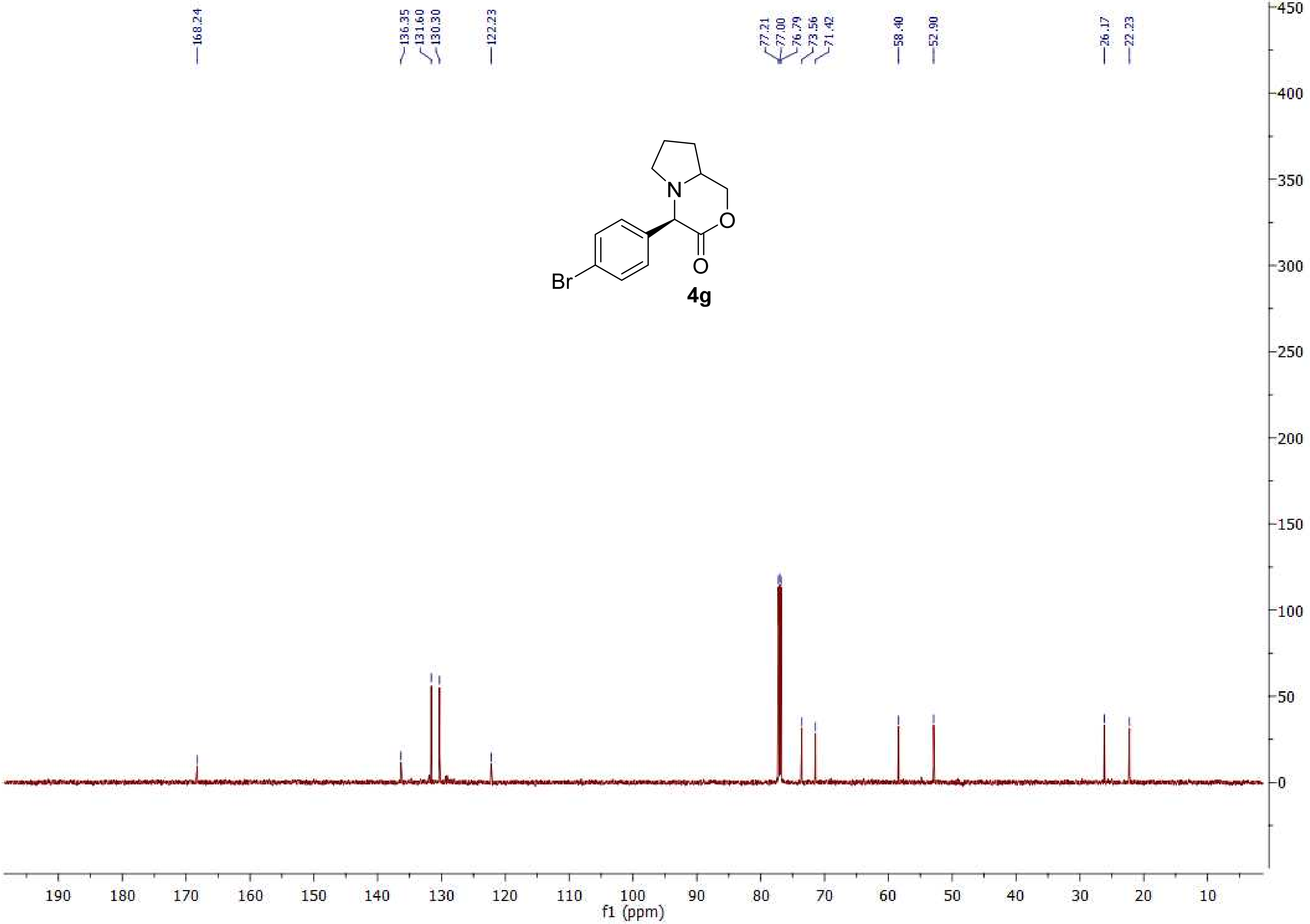

$^{1}$H NMR in $CDCl_3$ (300 MHz)

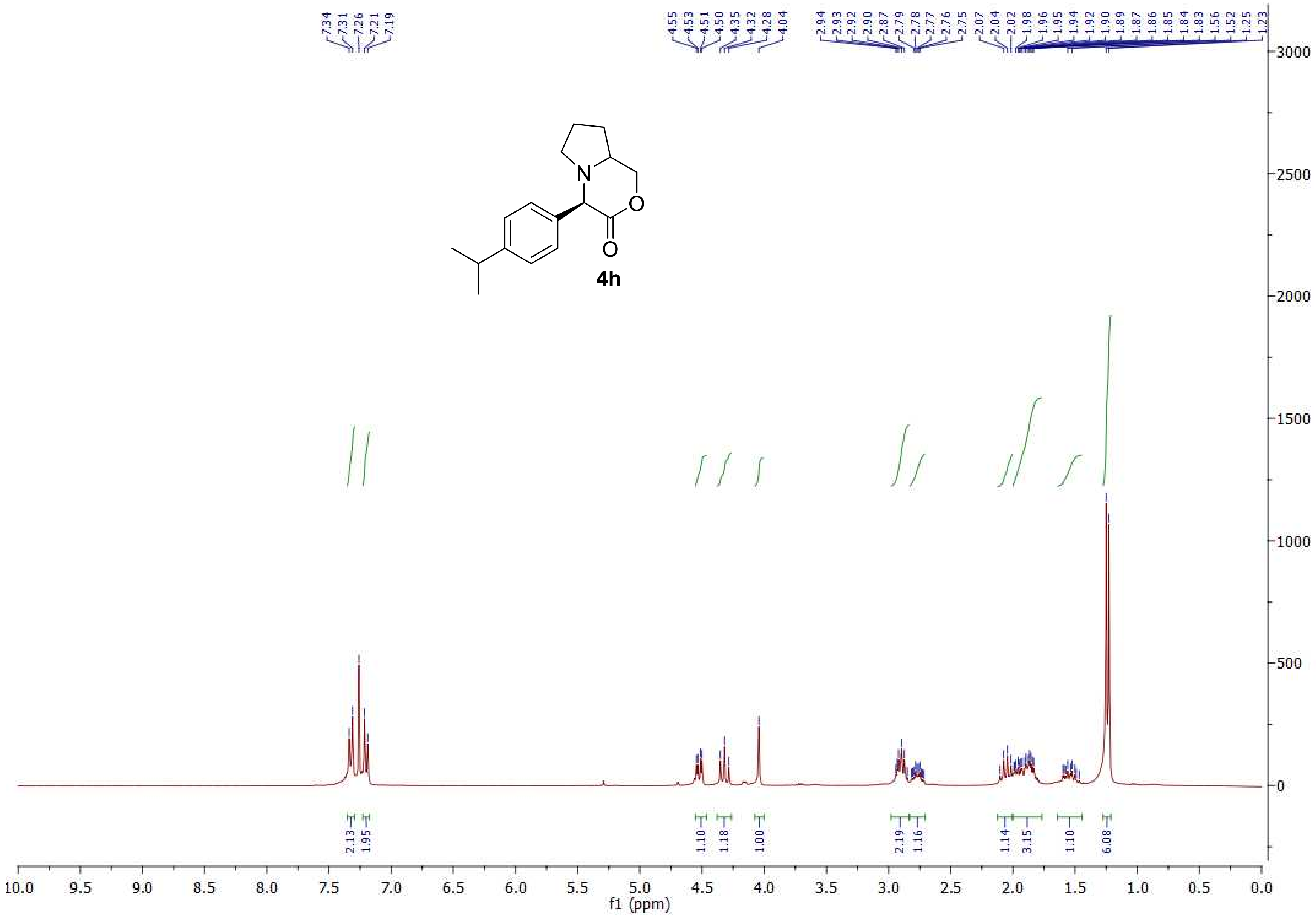

**$^{13}C\{^1H\}$** NMR in $CDCl_3$ (75 MHz)

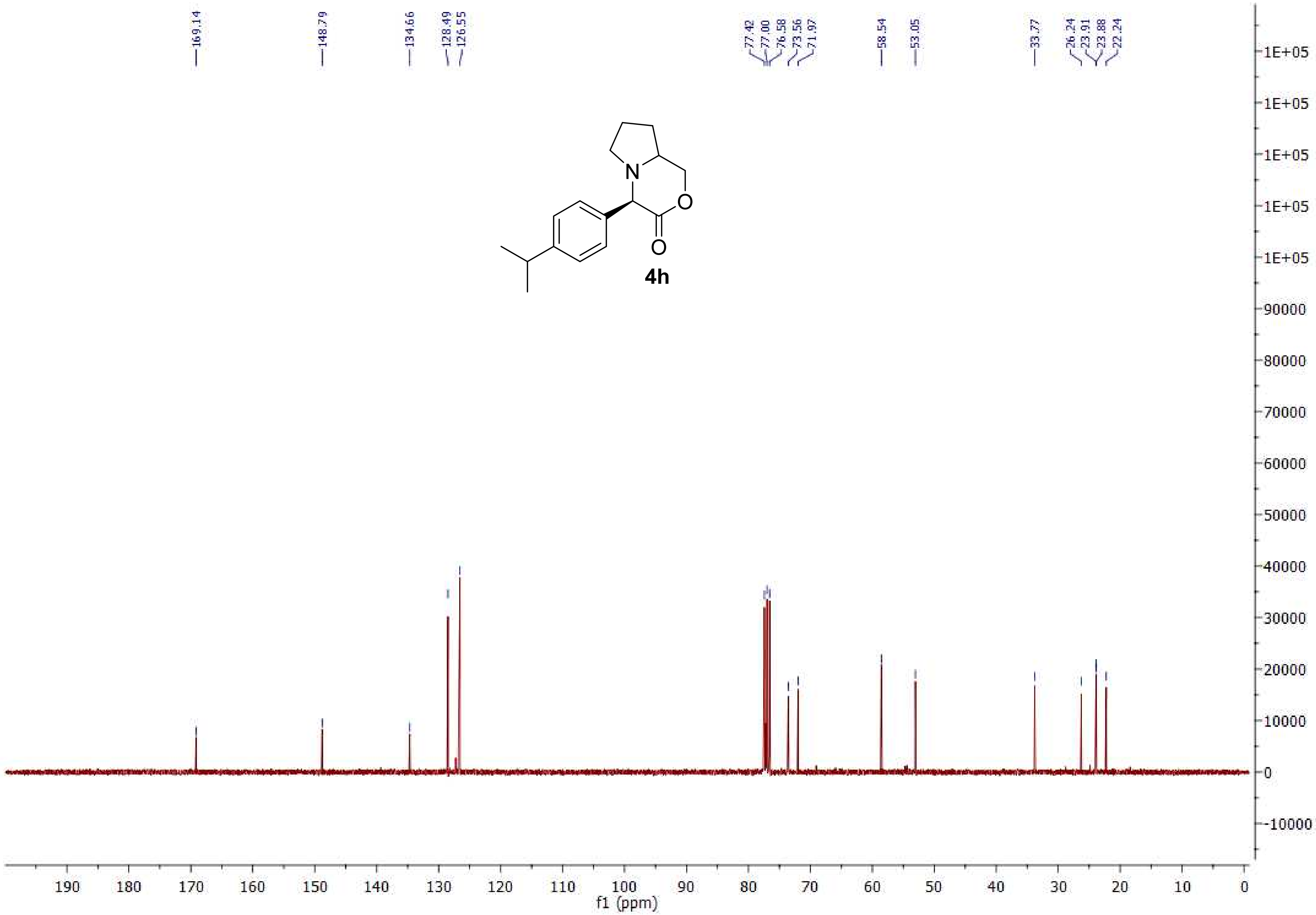

$^{1}H$ NMR in $CDCl_3$ (600 MHz)

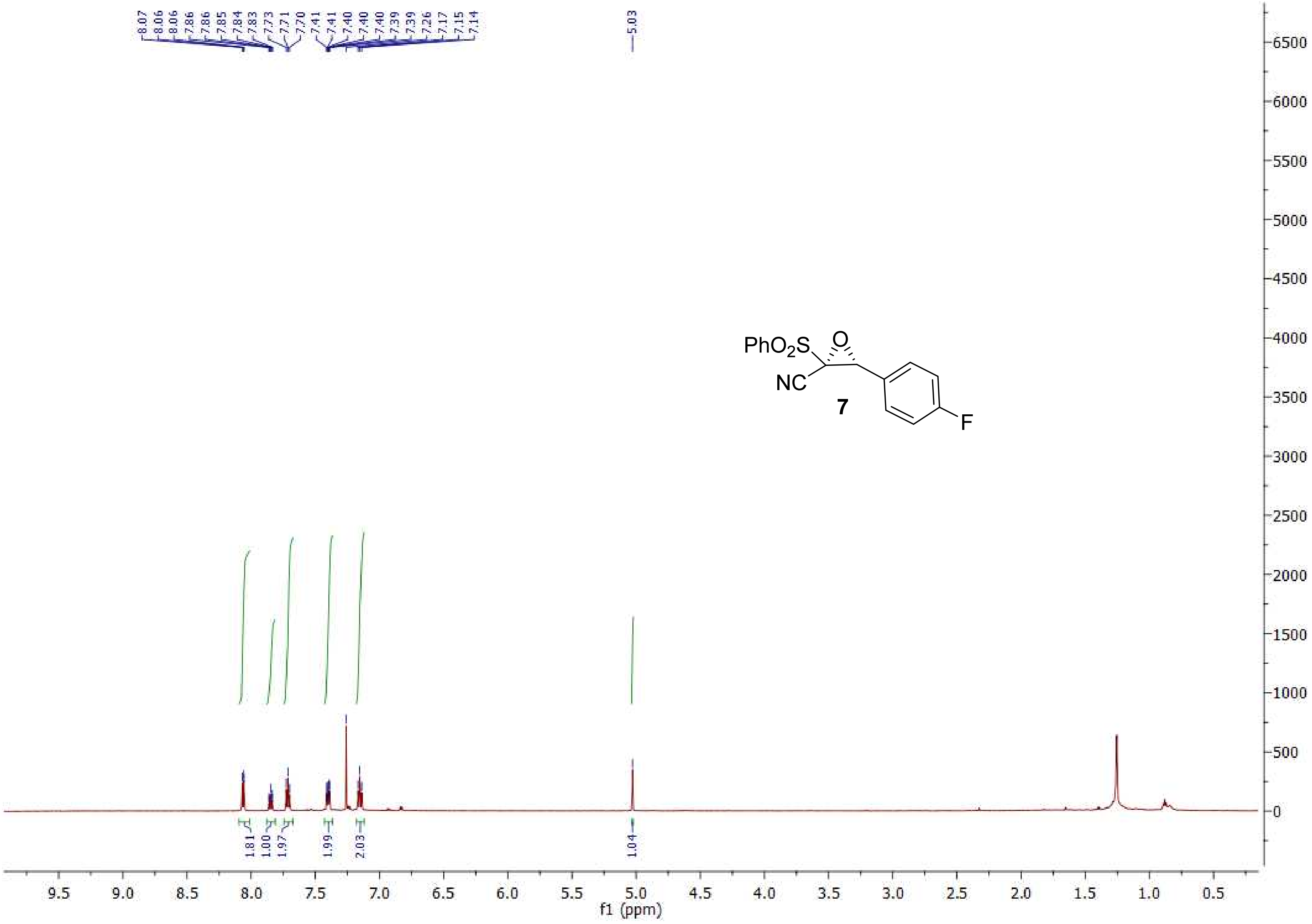

**$^{13}$C{$^{1}$H}** NMR in $CDCl_3$ (150 MHz)

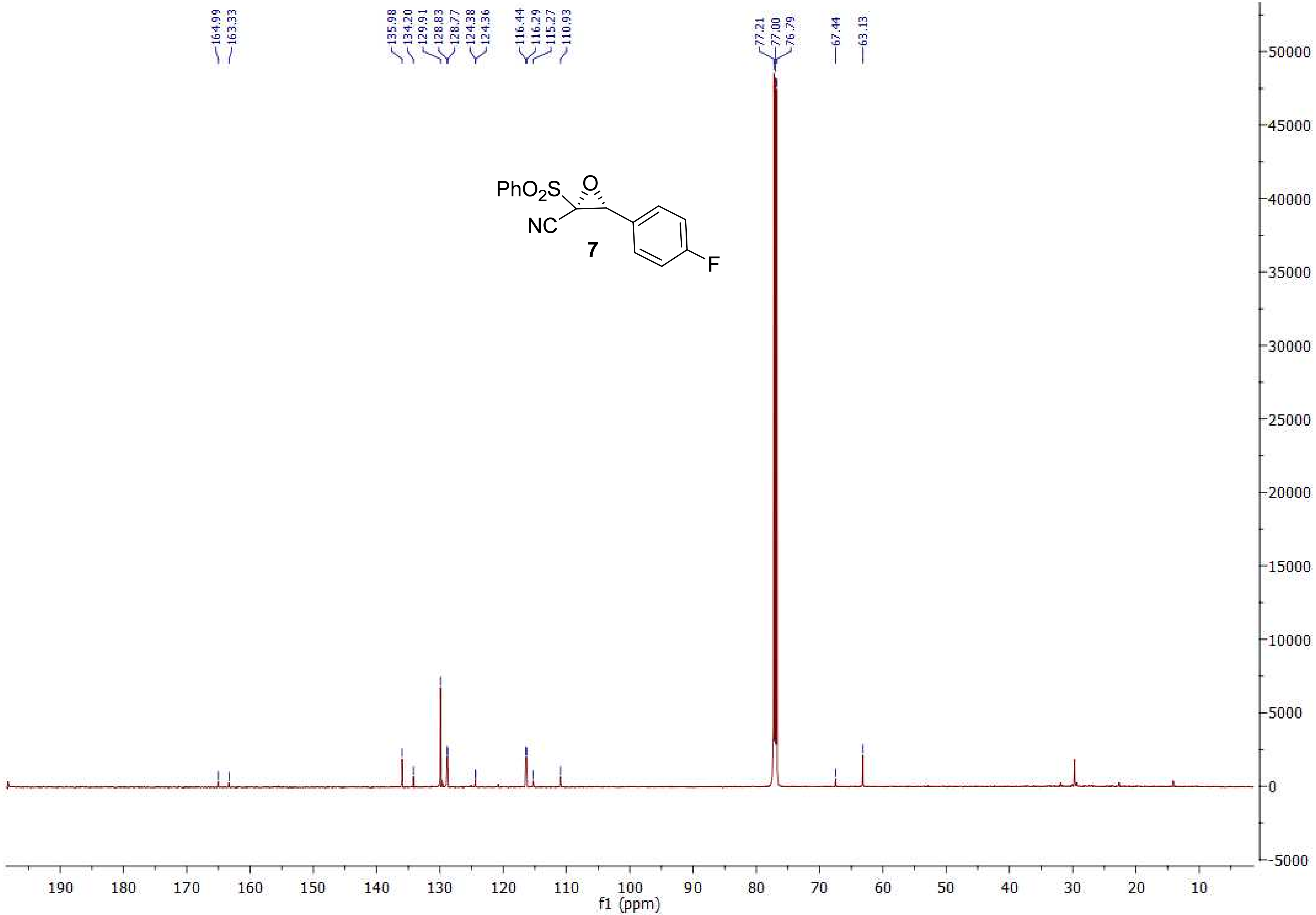

## HPLC chromatograms

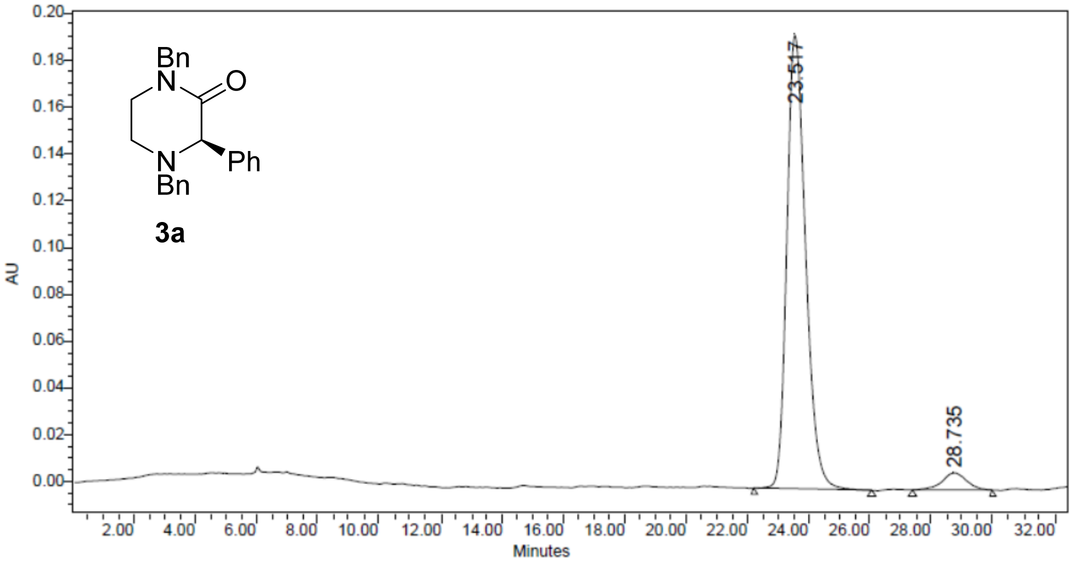


| | RT (min) | Area (□V*sec) | % Area | Height (□V) | % Height |
|---|---|---|---|---|---|
| 1 | 23.517 | 8265794 | 95.23 | 193732 | 96.37 |
| 2 | 28.735 | 414079 | 4.77 | 7298 | 3.63 |

**The ee value of compound 3a was determined using HPLC conditions reported in literature[5a,b]**

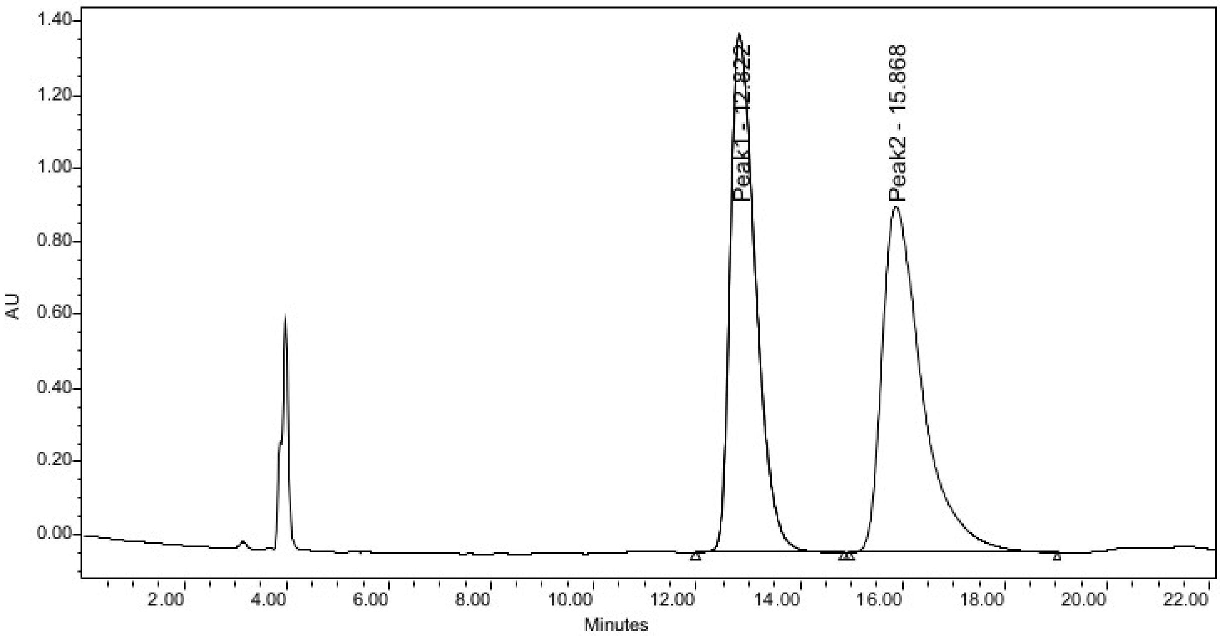


| | Peak Name | RT (min) | Area (V*sec) | % Area | Height (V) | % Height |
|---|---|---|---|---|---|---|
| 1 | Peak1 | 12.822 | 48935608 | 48.59 | 1413776 | 59.94 |
| 2 | Peak2 | 15.868 | 51780302 | 51.41 | 944814 | 40.06 |

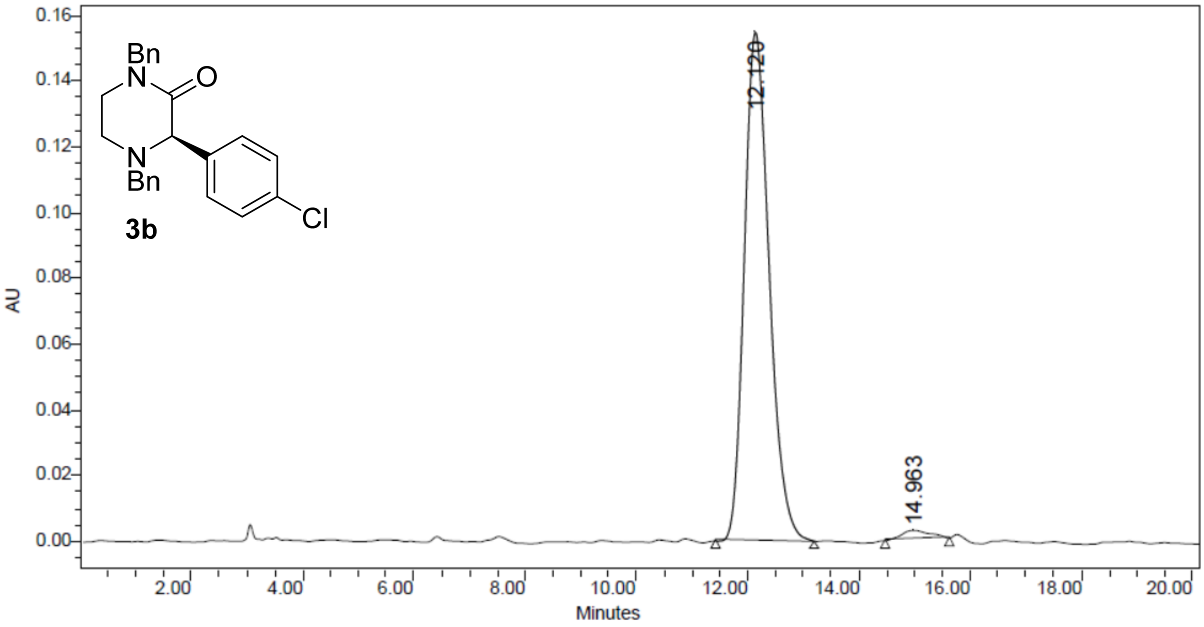


| | RT (min) | Area (□V*sec) | % Area | Height (□V) | % Height |
|---|---|---|---|---|---|
| 1 | 12.120 | 4820143 | 97.96 | 155118 | 98.24 |
| 2 | 14.963 | 100292 | 2.04 | 2771 | 1.76 |

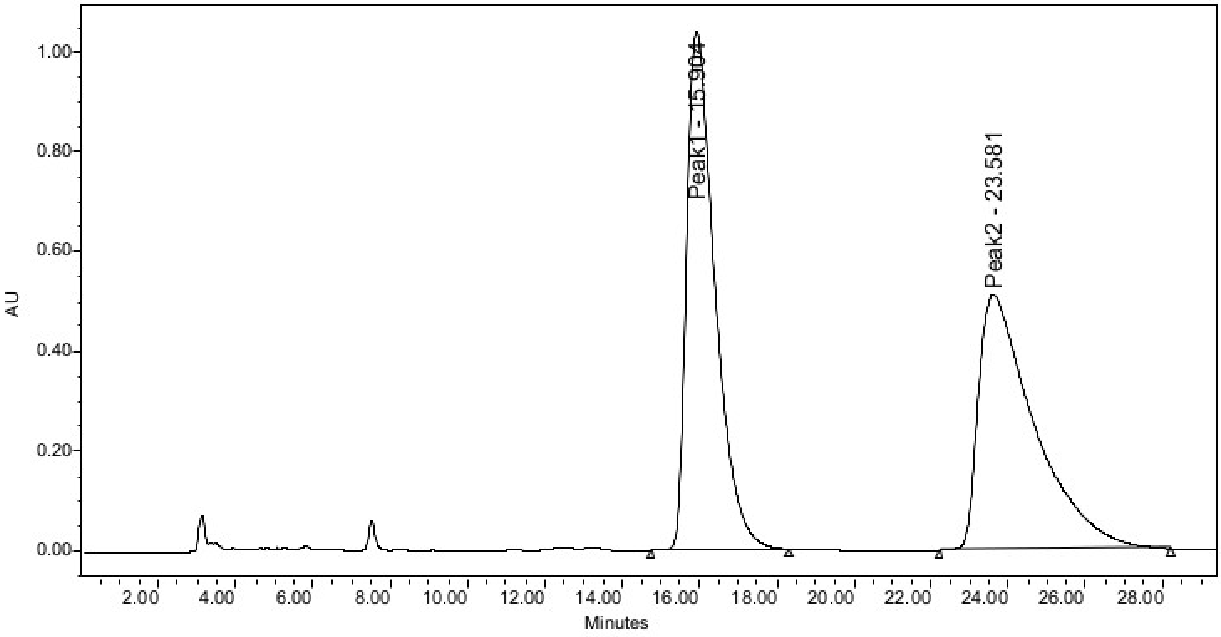


| | Peak Name | RT (min) | Area (V*sec) | % Area | Height (V) | % Height |
|---|---|---|---|---|---|---|
| 1 | Peak1 | 15.904 | 53163971 | 50.68 | 1038385 | 66.96 |
| 2 | Peak2 | 23.581 | 51743182 | 49.32 | 512360 | 33.04 |

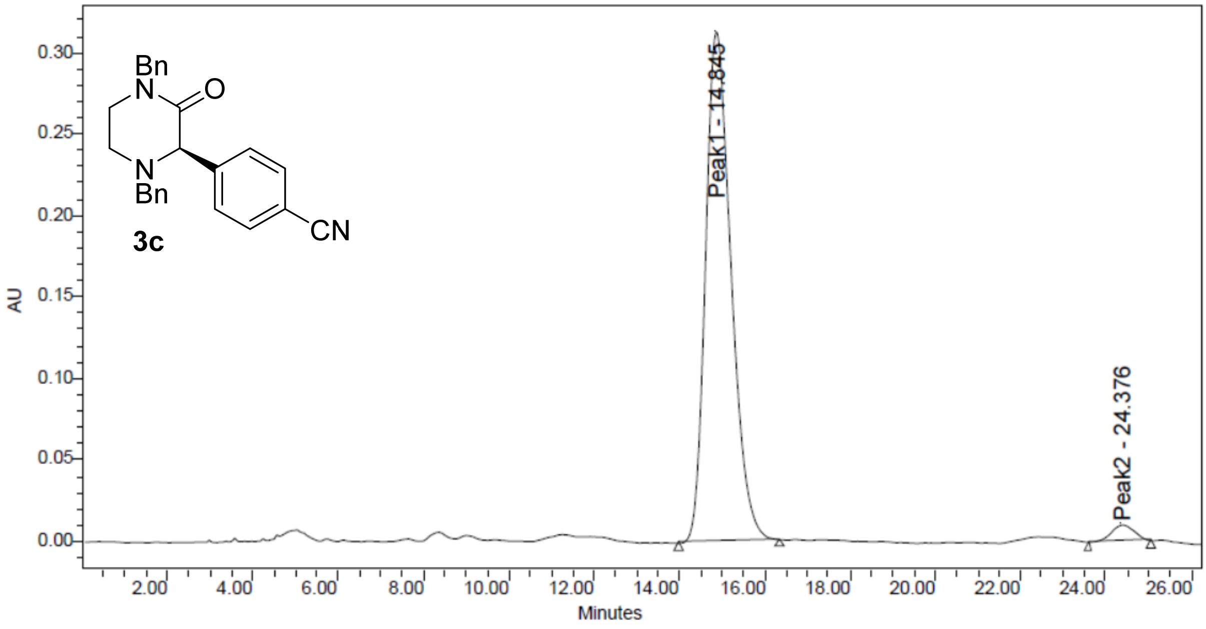


| | Peak Name | RT (min) | Area (□V*sec) | % Area | Height (□V) | % Height |
|---|---|---|---|---|---|---|
| 1 | Peak1 | 14.845 | 12996330 | 97.11 | 312969 | 96.84 |
| 2 | Peak2 | 24.376 | 386705 | 2.89 | 10200 | 3.16 |

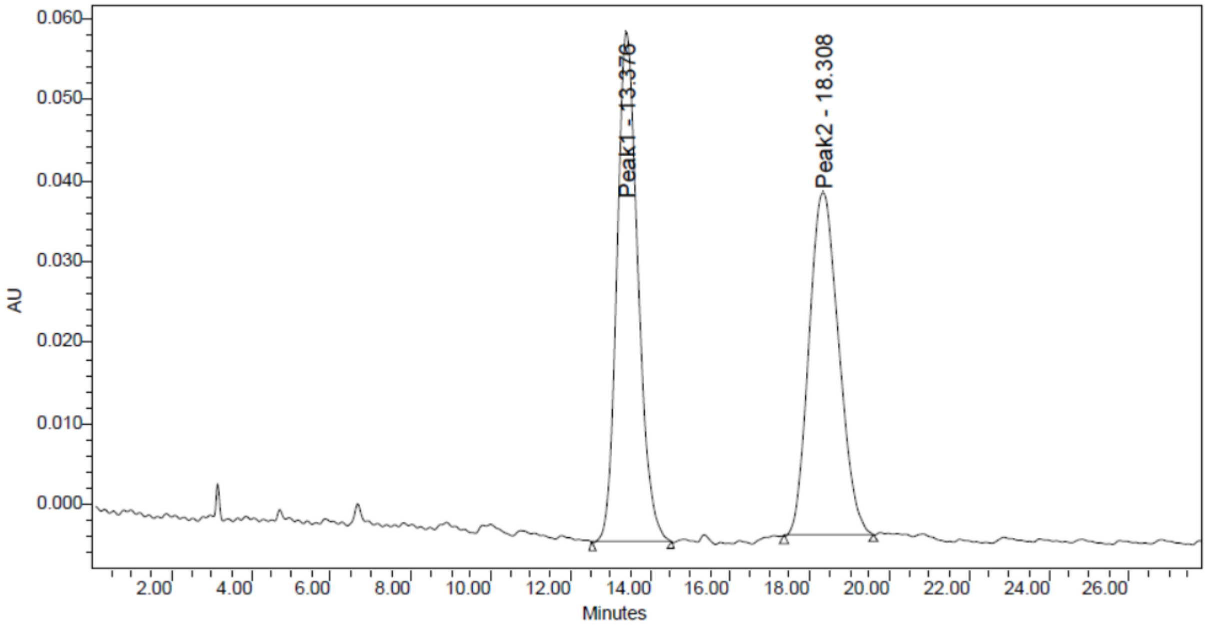


| | Peak Name | RT (min) | Area (□V*sec) | % Area | Height (□V) | % Height |
|---|---|---|---|---|---|---|
| 1 | Peak1 | 13.376 | 2310614 | 51.25 | 62910 | 59.82 |
| 2 | Peak2 | 18.308 | 2198195 | 48.75 | 42264 | 40.18 |

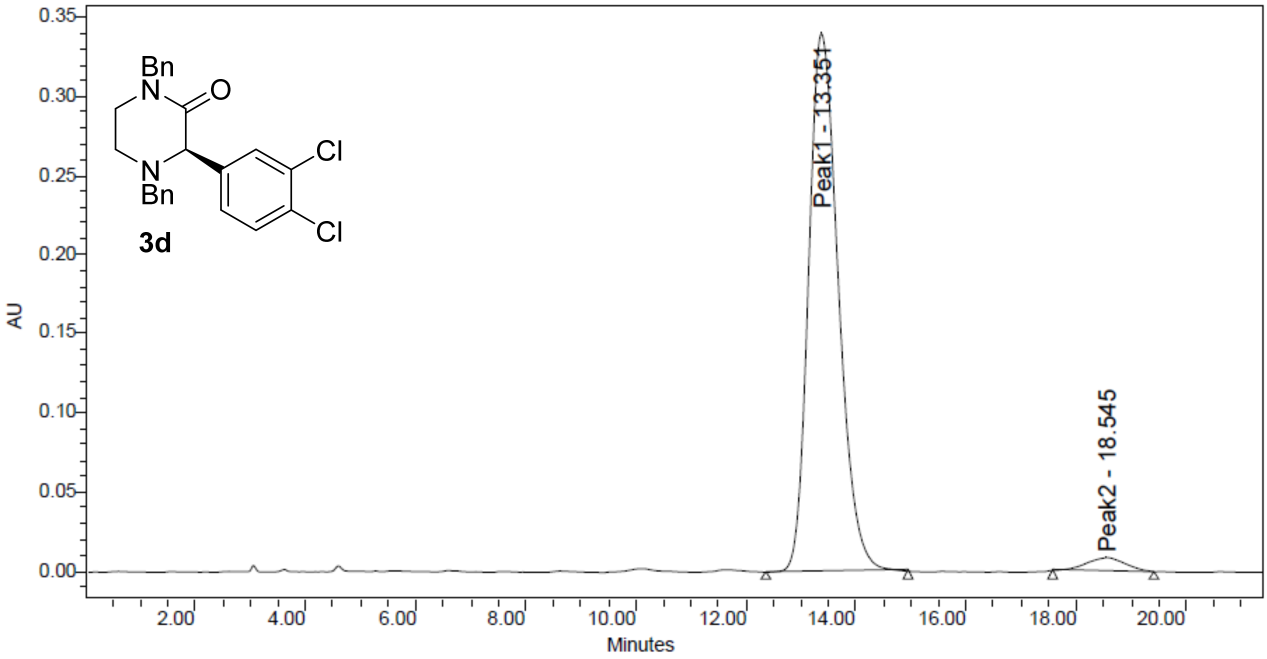


| | Peak Name | RT (min) | Area (□V*sec) | % Area | Height (□V) | % Height |
|---|---|---|---|---|---|---|
| 1 | Peak1 | 13.351 | 12825279 | 96.83 | 340318 | 97.56 |
| 2 | Peak2 | 18.545 | 420113 | 3.17 | 8521 | 2.44 |

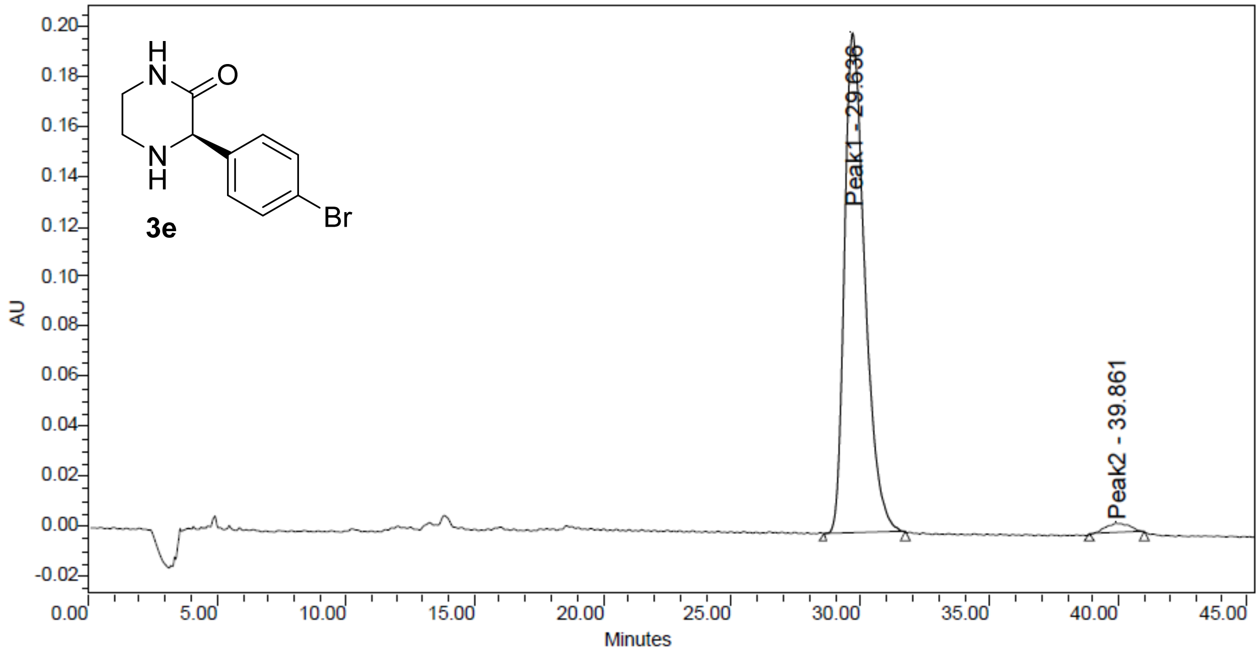


| | Peak Name | RT (min) | Area (□V*sec) | % Area | Height (□V) | % Height |
|---|---|---|---|---|---|---|
| 1 | Peak1 | 29.636 | 11005743 | 97.48 | 199862 | 98.01 |
| 2 | Peak2 | 39.861 | 283944 | 2.52 | 4051 | 1.99 |

**The ee value of compound 3e was determined using HPLC conditions reported in literature[6]**

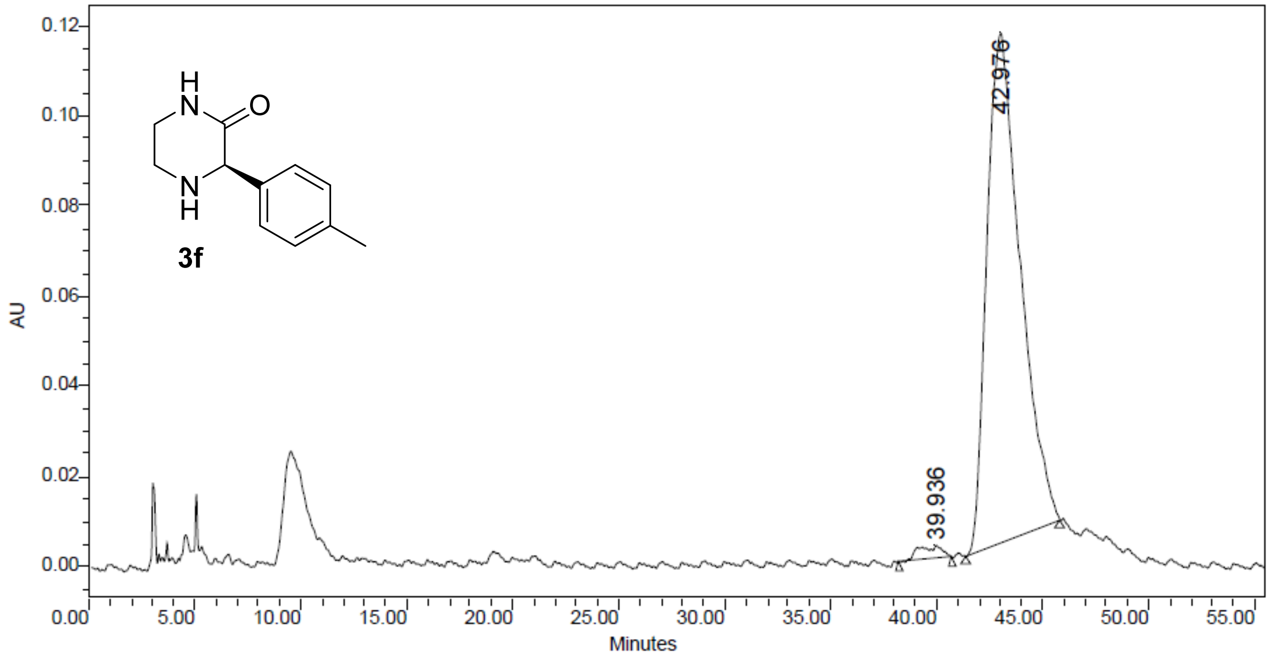


| | RT (min) | Area (▯V*sec) | % Area | Height (▯V) | % Height |
|---|---|---|---|---|---|
| 1 | 39.936 | 254153 | 2.01 | 3106 | 2.67 |
| 2 | 42.976 | 12418387 | 97.99 | 113138 | 97.33 |

**The ee value of compound 3e was determined using HPLC conditions reported in literature[6]**

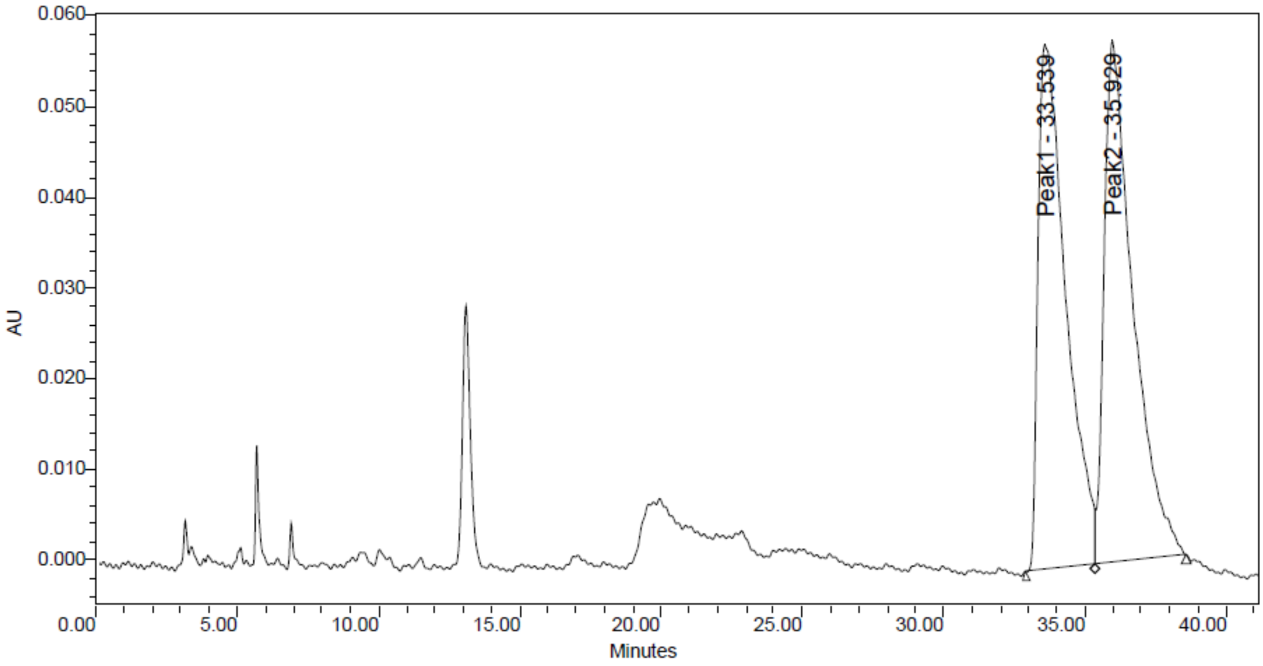


| | Peak Name | RT (min) | Area (▯V*sec) | % Area | Height (▯V) | % Height |
|---|---|---|---|---|---|---|
| 1 | Peak1 | 33.539 | 3990450 | 49.17 | 58006 | 50.15 |
| 2 | Peak2 | 35.929 | 4125411 | 50.83 | 57670 | 49.85 |

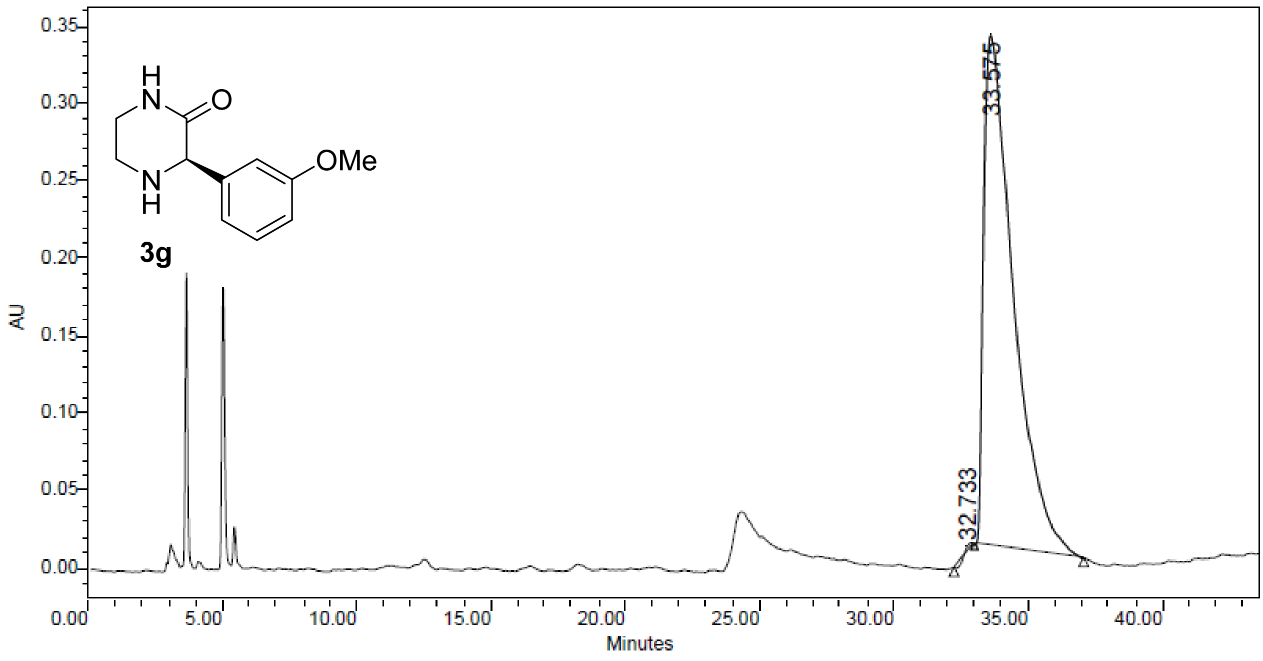


| | RT (min) | Area (▯V*sec) | % Area | Height (▯V) | % Height |
|---|---|---|---|---|---|
| 1 | 32.733 | 114071 | 0.45 | 5210 | 1.56 |
| 2 | 33.575 | 25288215 | 99.55 | 328503 | 98.44 |

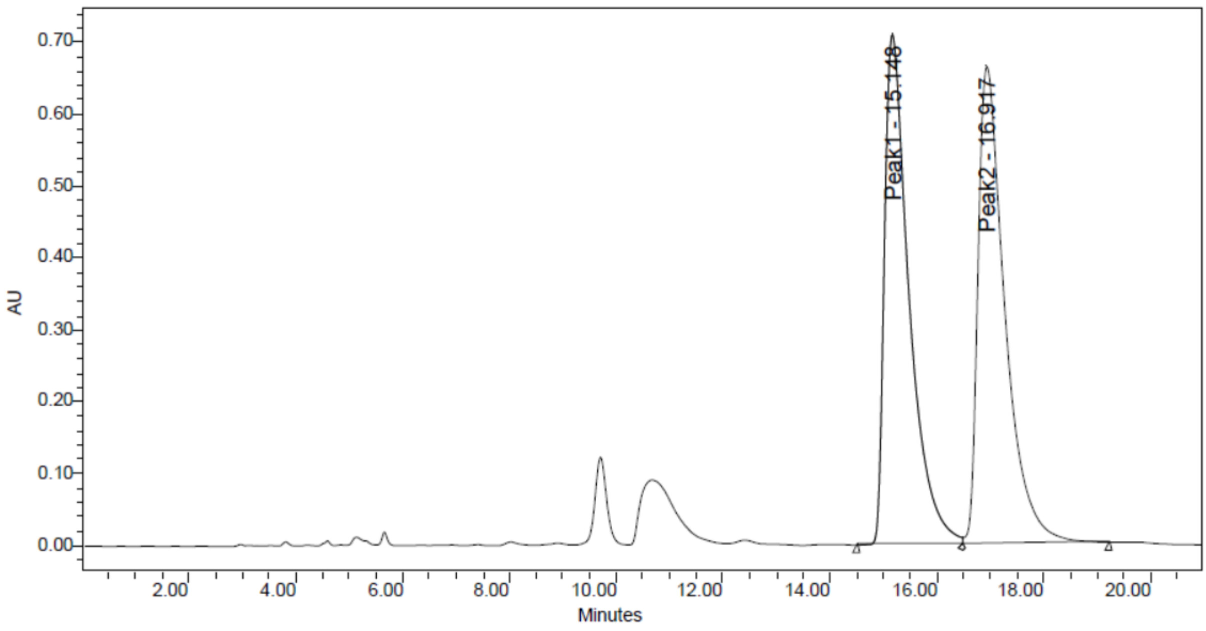


| | Peak Name | RT (min) | Area (□V*sec) | % Area | Height (□V) | % Height |
|---|---|---|---|---|---|---|
| 1 | Peak1 | 15.148 | 22809568 | 49.45 | 710915 | 51.67 |
| 2 | Peak2 | 16.917 | 23321424 | 50.55 | 664845 | 48.33 |

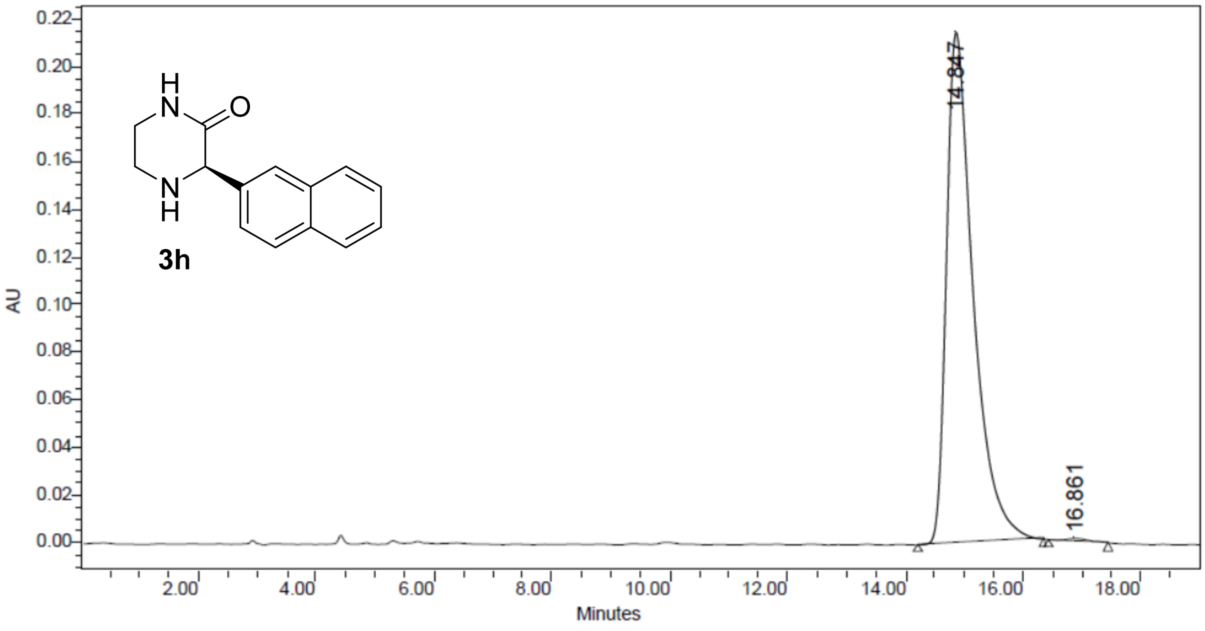


| | RT (min) | Area (□V*sec) | % Area | Height (□V) | % Height |
|---|---|---|---|---|---|
| 1 | 14.847 | 6768359 | 99.57 | 214395 | 99.47 |
| 2 | 16.861 | 29082 | 0.43 | 1145 | 0.53 |

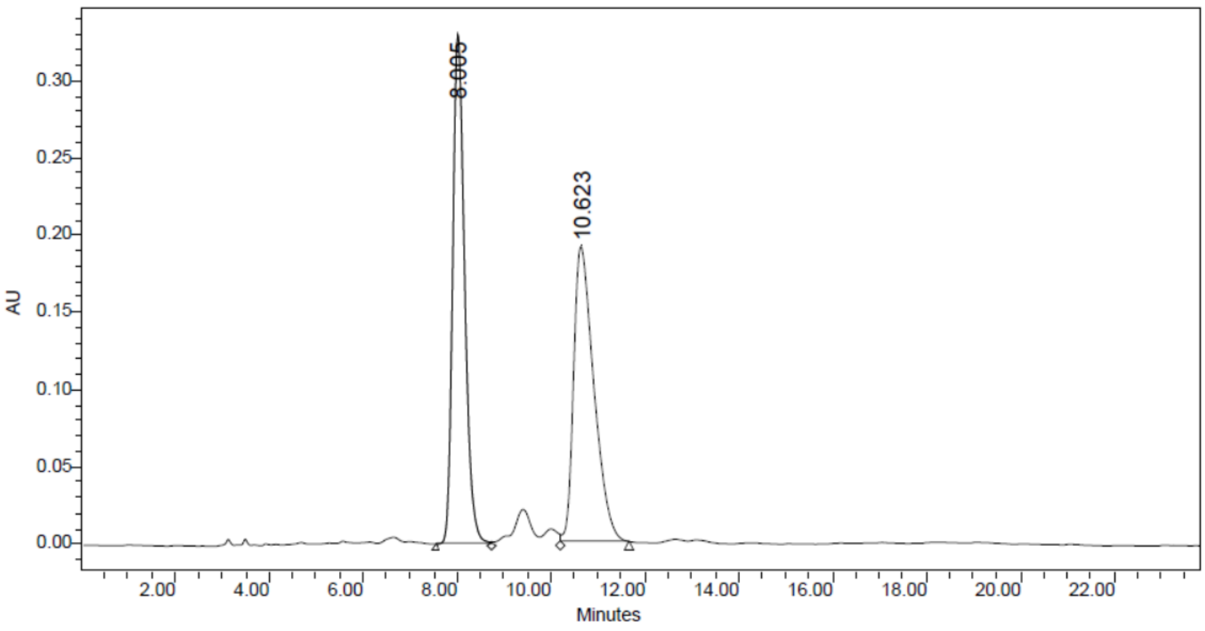


| | RT (min) | Area (□V*sec) | % Area | Height (□V) | % Height |
|---|---|---|---|---|---|
| 1 | 8.005 | 5813570 | 50.28 | 329585 | 63.26 |
| 2 | 10.623 | 5748614 | 49.72 | 191387 | 36.74 |

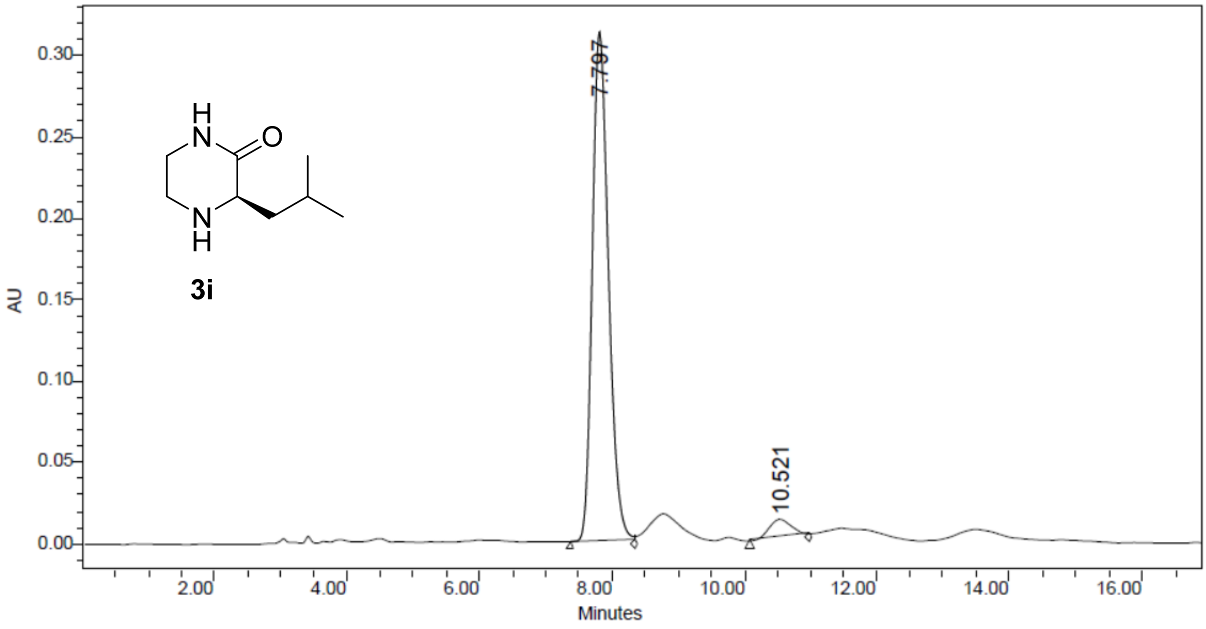


| | RT (min) | Area (□V*sec) | % Area | Height (□V) | % Height |
|---|---|---|---|---|---|
| 1 | 7.797 | 5324180 | 95.16 | 313702 | 96.49 |
| 2 | 10.521 | 270687 | 4.84 | 11403 | 3.51 |

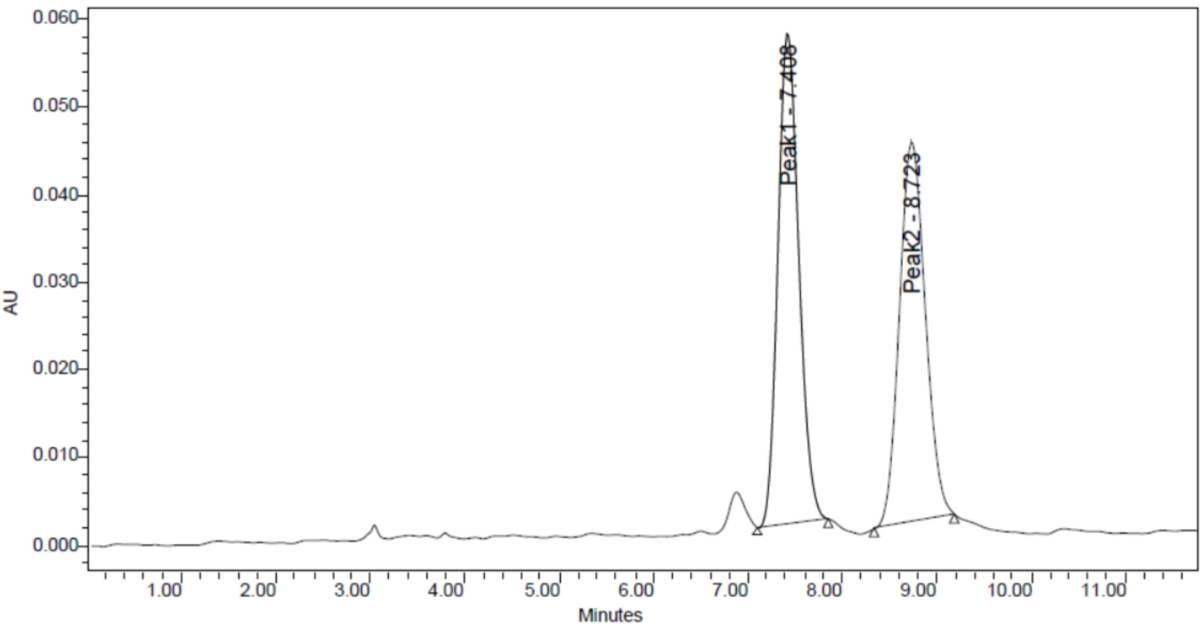


| | Peak Name | RT (min) | Area (□V*sec) | % Area | Height (□V) | % Height |
|---|---|---|---|---|---|---|
| 1 | Peak1 | 7.408 | 863626 | 51.03 | 56079 | 56.36 |
| 2 | Peak2 | 8.723 | 828723 | 48.97 | 43430 | 43.64 |

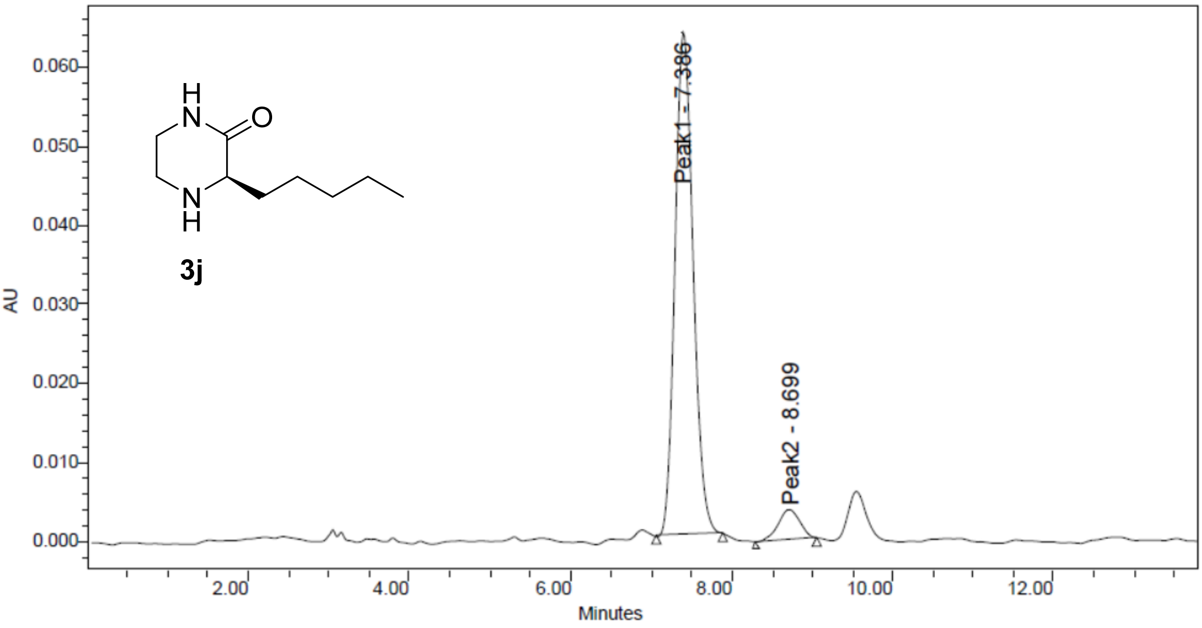


| | Peak Name | RT (min) | Area (□V*sec) | % Area | Height (□V) | % Height |
|---|---|---|---|---|---|---|
| 1 | Peak1 | 7.386 | 996619 | 93.24 | 63622 | 94.34 |
| 2 | Peak2 | 8.699 | 72205 | 6.76 | 3819 | 5.66 |

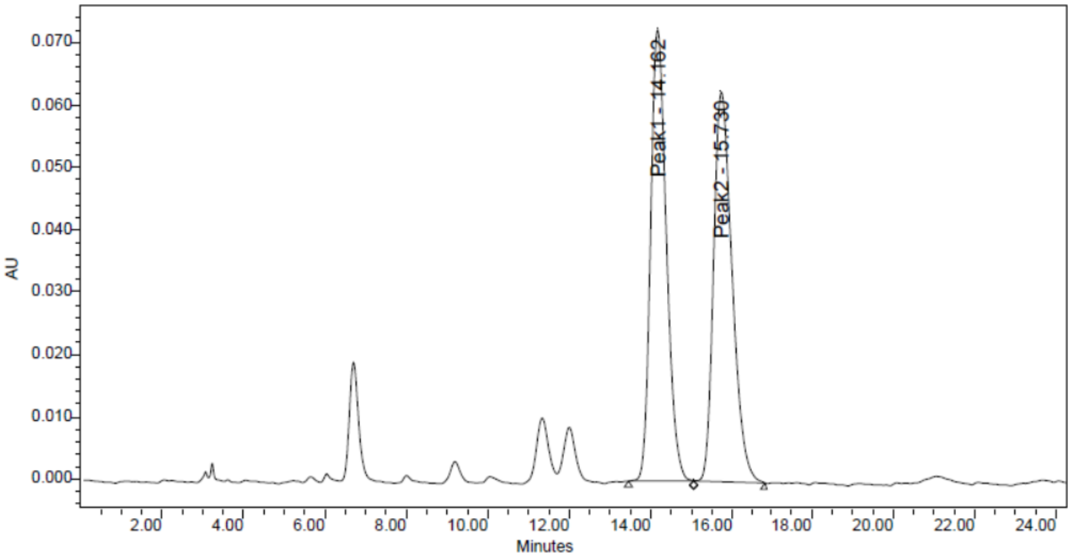


| | Peak Name | RT (min) | Area (µV*sec) | % Area | Height (µV) | % Height |
|---|---|---|---|---|---|---|
| 1 | Peak1 | 14.162 | 1960291 | 49.74 | 72463 | 53.66 |
| 2 | Peak2 | 15.730 | 1981116 | 50.26 | 62587 | 46.34 |

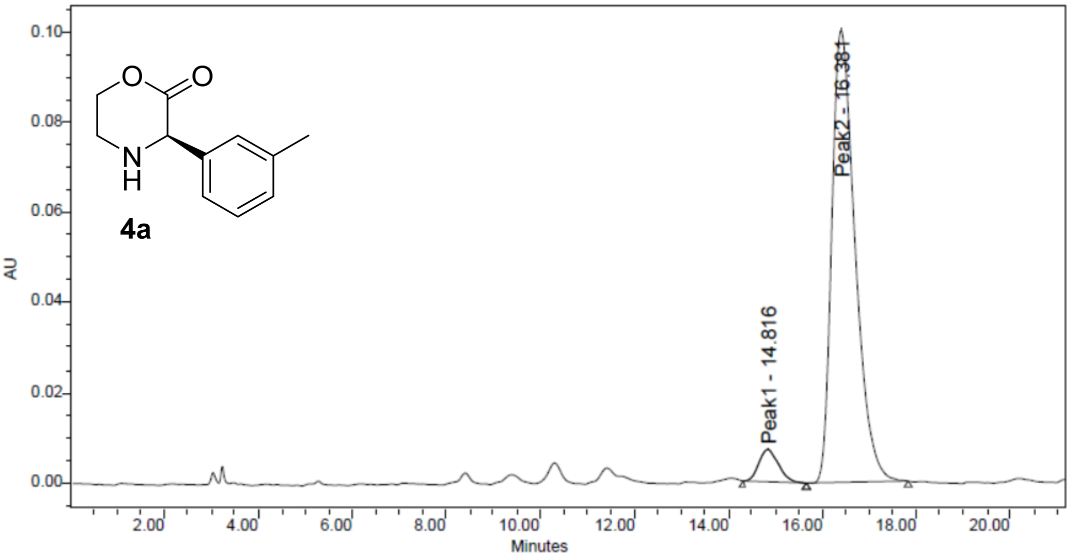


| | Peak Name | RT (min) | Area (µV*sec) | % Area | Height (µV) | % Height |
|---|---|---|---|---|---|---|
| 1 | Peak1 | 14.816 | 208768 | 5.71 | 7173 | 6.67 |
| 2 | Peak2 | 16.381 | 3450549 | 94.29 | 100383 | 93.33 |

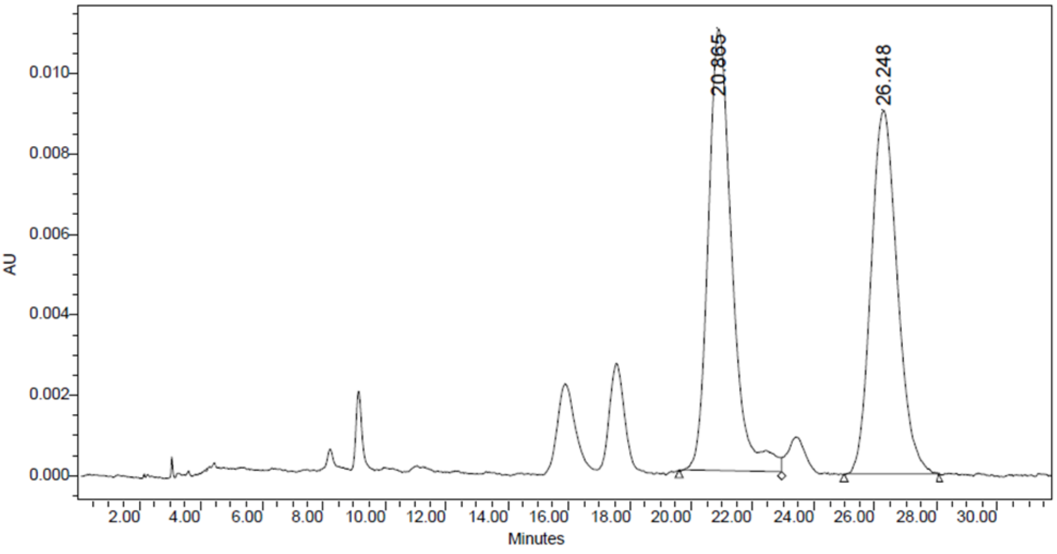


| | RT (min) | Area (µV*sec) | % Area | Height (µV) | % Height |
|---|---|---|---|---|---|
| 1 | 20.865 | 597805 | 52.27 | 11054 | 54.87 |
| 2 | 26.248 | 545892 | 47.73 | 9091 | 45.13 |

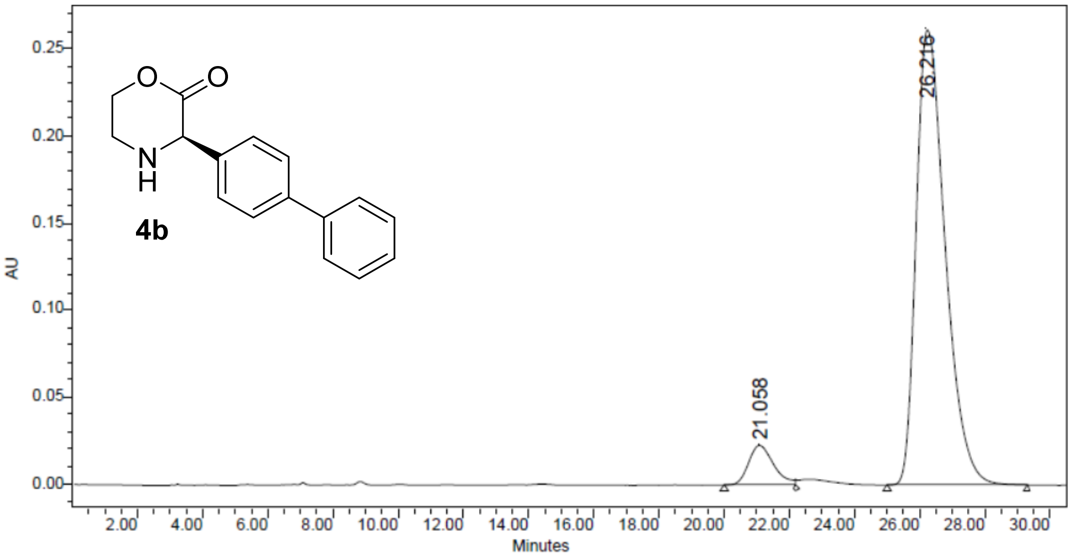


| | RT (min) | Area (µV*sec) | % Area | Height (µV) | % Height |
|---|---|---|---|---|---|
| 1 | 21.058 | 1232345 | 7.06 | 22808 | 8.01 |
| 2 | 26.216 | 16233051 | 92.94 | 261924 | 91.99 |

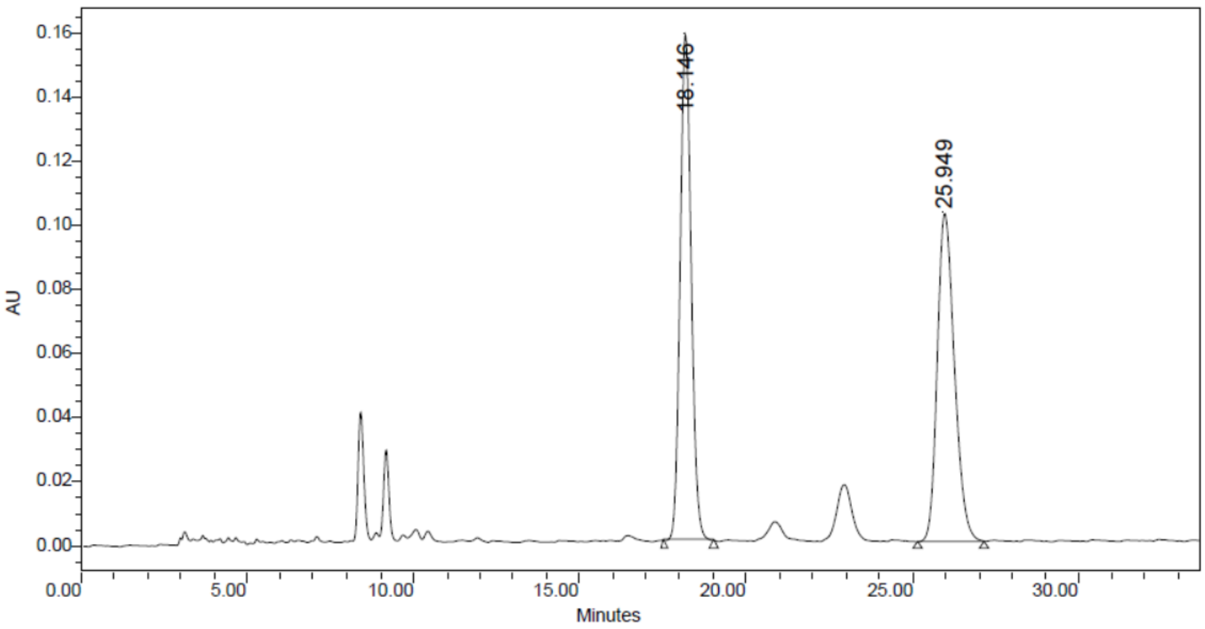


| | RT (min) | Area (µV*sec) | % Area | Height (µV) | % Height |
|---|---|---|---|---|---|
| 1 | 18.146 | 3647131 | 49.82 | 157600 | 60.67 |
| 2 | 25.949 | 3673944 | 50.18 | 102159 | 39.33 |

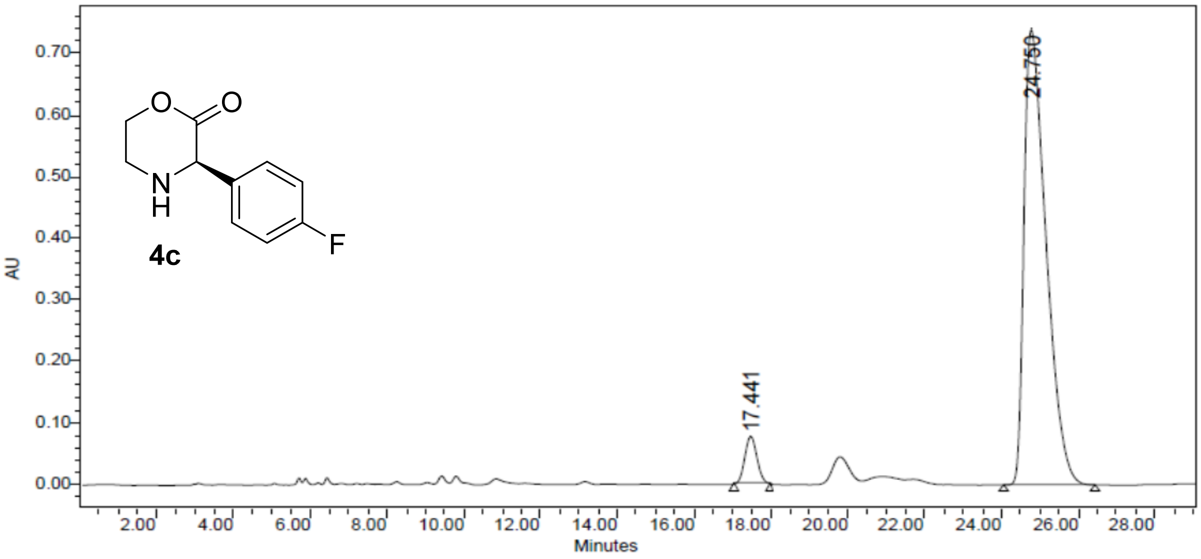


| | RT (min) | Area (µV*sec) | % Area | Height (µV) | % Height |
|---|---|---|---|---|---|
| 1 | 17.441 | 1705022 | 5.46 | 77088 | 9.44 |
| 2 | 24.750 | 29506758 | 94.54 | 739401 | 90.56 |

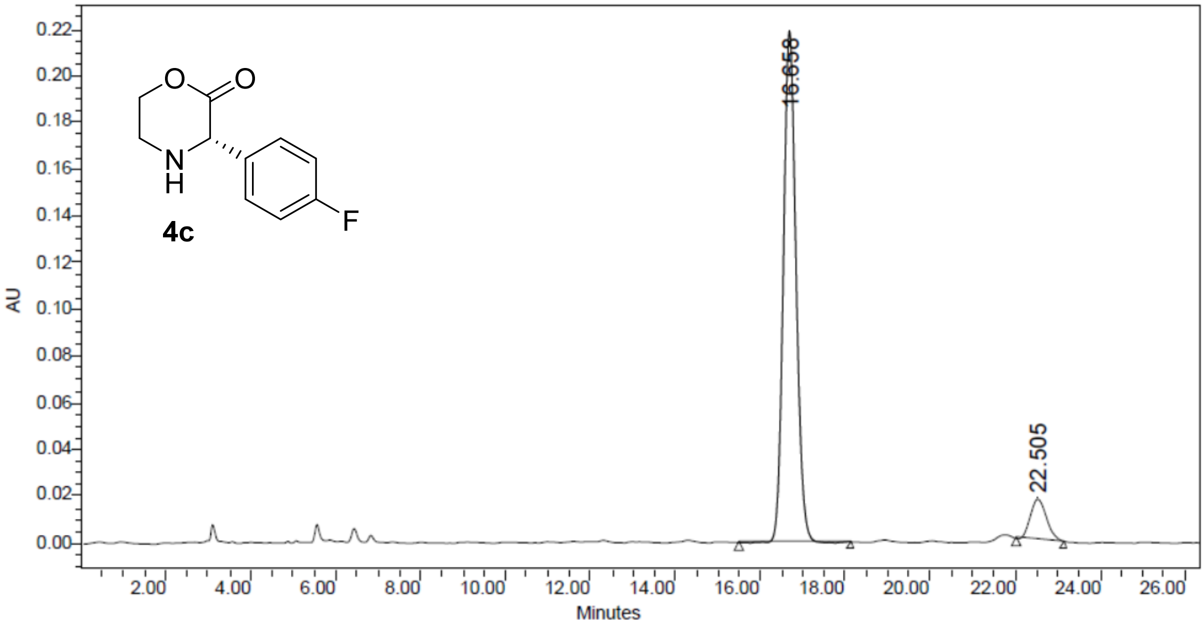


| | RT (min) | Area (µV*sec) | % Area | Height (µV) | % Height |
|---|---|---|---|---|---|
| 1 | 16.658 | 4528619 | 90.72 | 218868 | 92.62 |
| 2 | 22.505 | 463140 | 9.28 | 17428 | 7.38 |

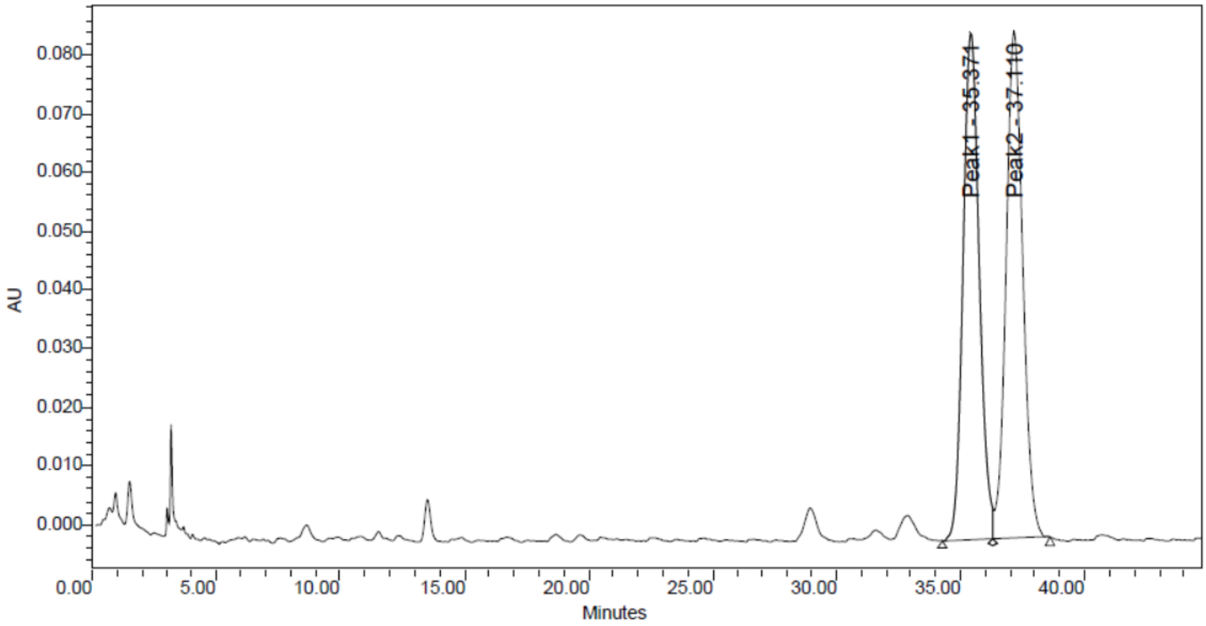


| | Peak Name | RT (min) | Area (µV*sec) | % Area | Height (µV) | % Height |
|---|---|---|---|---|---|---|
| 1 | Peak1 | 35.371 | 3945320 | 49.38 | 86379 | 49.98 |
| 2 | Peak2 | 37.110 | 4044130 | 50.62 | 86436 | 50.02 |

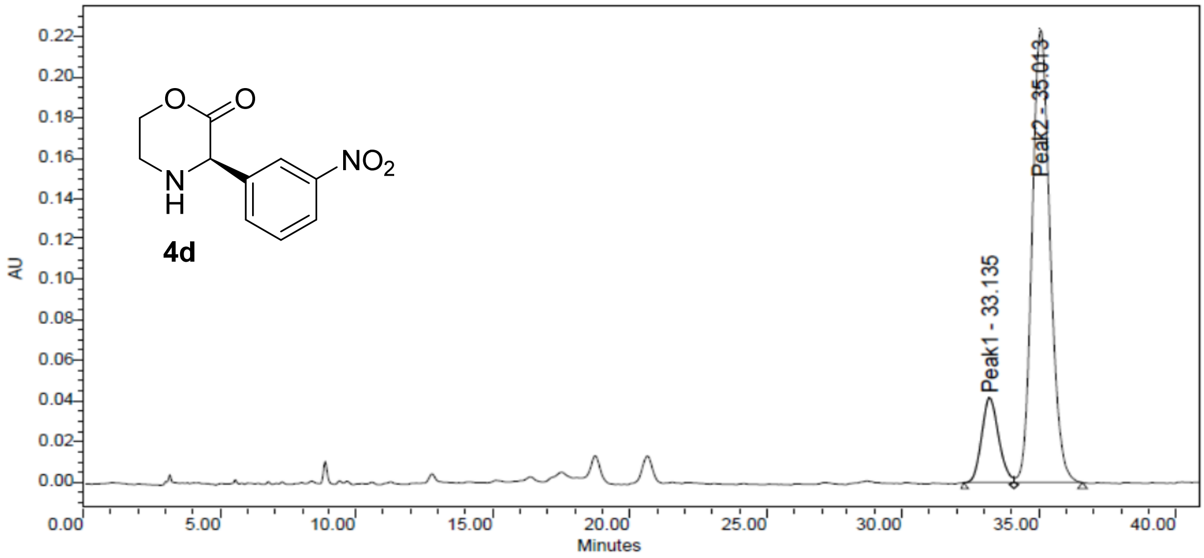


| | Peak Name | RT (min) | Area (µV*sec) | % Area | Height (µV) | % Height |
|---|---|---|---|---|---|---|
| 1 | Peak1 | 33.135 | 1819320 | 15.11 | 41768 | 15.79 |
| 2 | Peak2 | 35.013 | 10220710 | 84.89 | 222749 | 84.21 |

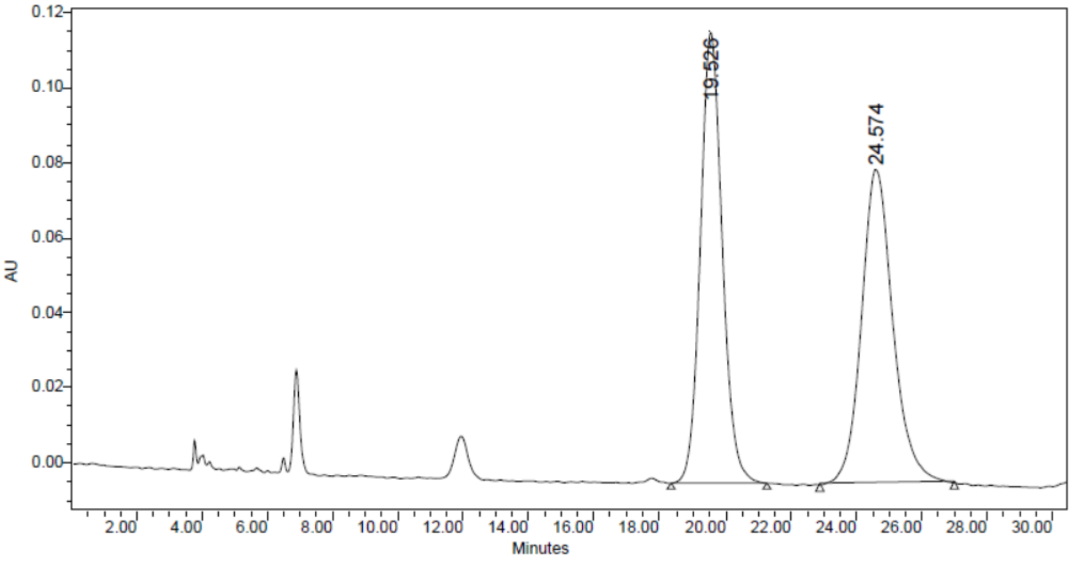


| | RT (min) | Area (µV*sec) | % Area | Height (µV) | % Height |
|---|---|---|---|---|---|
| 1 | 19.526 | 5606304 | 49.79 | 120246 | 58.90 |
| 2 | 24.574 | 5653756 | 50.21 | 83909 | 41.10 |

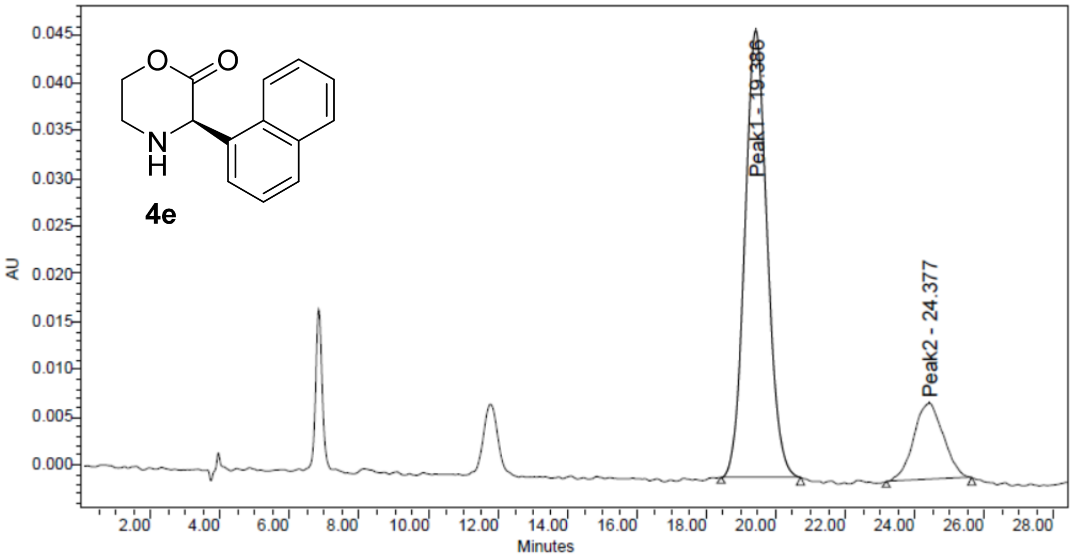


| | Peak Name | RT (min) | Area (µV*sec) | % Area | Height (µV) | % Height |
|---|---|---|---|---|---|---|
| 1 | Peak1 | 19.386 | 2101524 | 81.28 | 46909 | 85.37 |
| 2 | Peak2 | 24.377 | 484106 | 18.72 | 8041 | 14.63 |

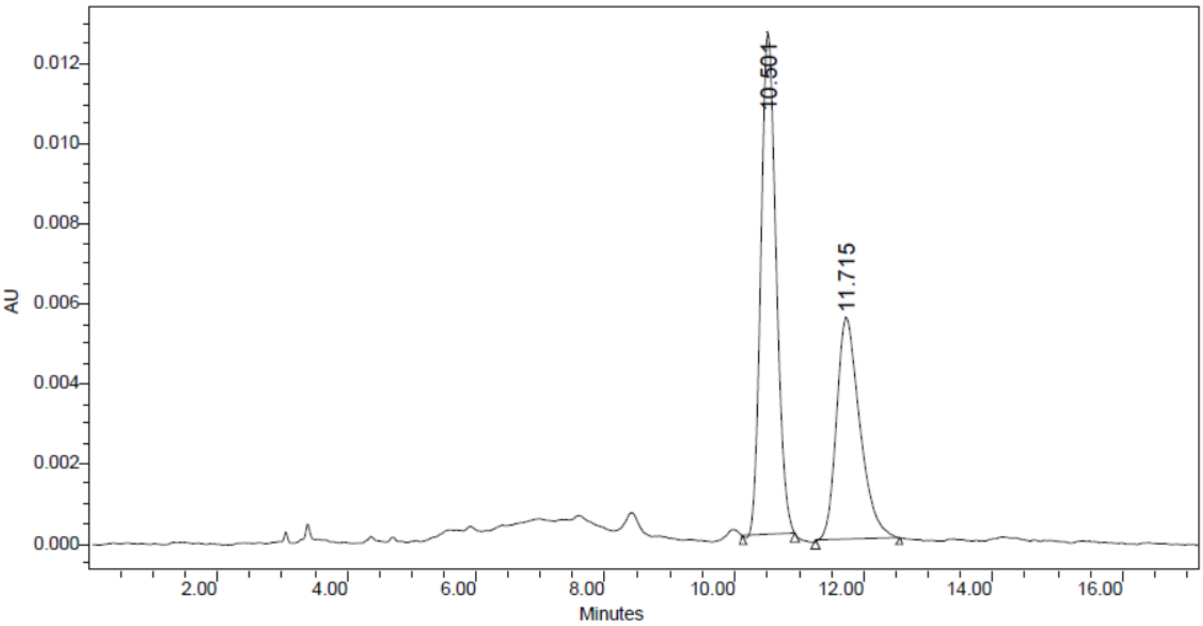


| | RT (min) | Area (□V*sec) | % Area | Height (□V) | % Height |
|---|---|---|---|---|---|
| 1 | 10.501 | 208558 | 59.73 | 12580 | 69.34 |
| 2 | 11.715 | 140618 | 40.27 | 5563 | 30.66 |

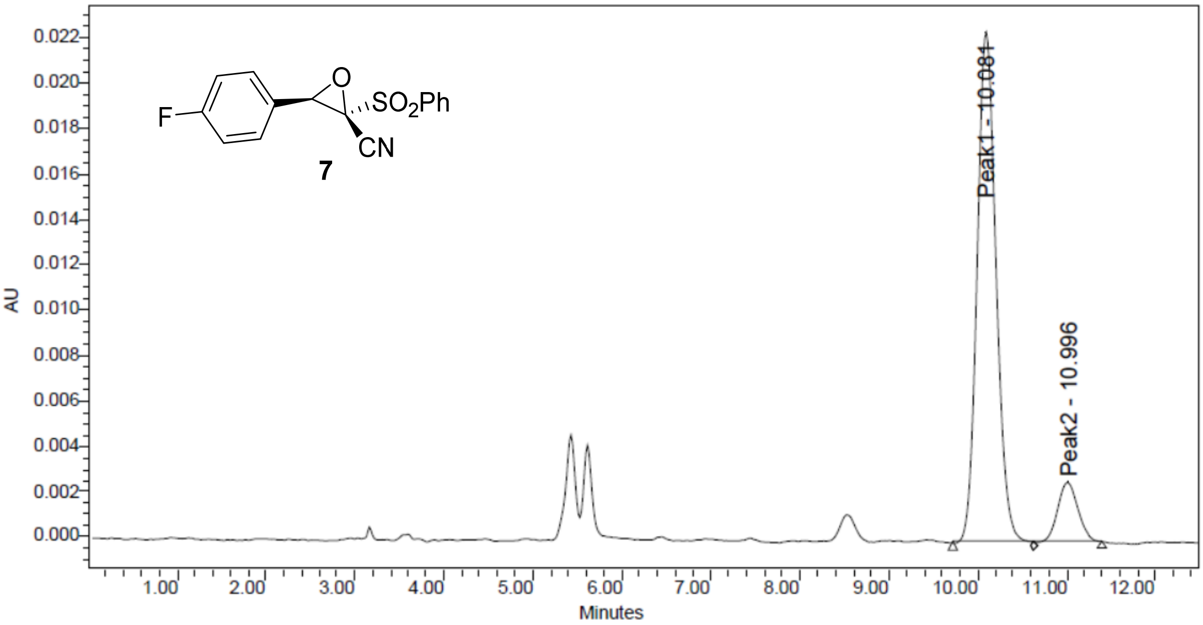


| | Peak Name | RT (min) | Area (□V*sec) | % Area | Height (□V) | % Height |
|---|---|---|---|---|---|---|
| 1 | Peak1 | 10.081 | 331591 | 88.78 | 22516 | 89.53 |
| 2 | Peak2 | 10.996 | 41908 | 11.22 | 2633 | 10.47 |
