# Supplementary figures and images for: Asymmetric Catalytic Access to Piperazin-2-ones and Morpholin-2-ones in a One-Pot Approach: Rapid Synthesis of an Intermediate to Aprepitant
Source: J Org Chem. 2023 Feb 21;88(12):7888–92. doi: 10.1021/acs.joc.2c02491 (PMC10278953; doi:10.1021/acs.joc.2c02491)

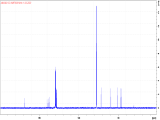

Supplement: Supplementary file 2 — jo2c02491_si_002.zip [file jo2c02491_si_002.zip › FID for publication/3a/13C/pdata/1/thumb.png]

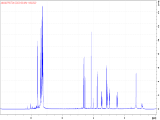

Supplement: Supplementary file 2 — jo2c02491_si_002.zip [file jo2c02491_si_002.zip › FID for publication/3a/1H/pdata/1/thumb.png]

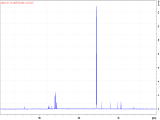

Supplement: Supplementary file 2 — jo2c02491_si_002.zip [file jo2c02491_si_002.zip › FID for publication/3b/13C/pdata/1/thumb.png]

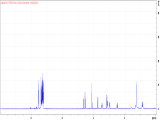

Supplement: Supplementary file 2 — jo2c02491_si_002.zip [file jo2c02491_si_002.zip › FID for publication/3b/1H/pdata/1/thumb.png]

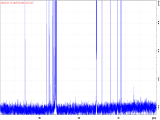

Supplement: Supplementary file 2 — jo2c02491_si_002.zip [file jo2c02491_si_002.zip › FID for publication/3d/13C/pdata/1/thumb.png]

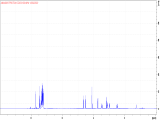

Supplement: Supplementary file 2 — jo2c02491_si_002.zip [file jo2c02491_si_002.zip › FID for publication/3d/1H/pdata/1/thumb.png]
