# Supplementary material for: Asymmetric Catalytic Access to Piperazin-2-ones and Morpholin-2-ones in a One-Pot Approach: Rapid Synthesis of an Intermediate to Aprepitant
Source: J Org Chem. 2023 Feb 21;88(12):7888–92. doi: 10.1021/acs.joc.2c02491 (PMC10278953; doi:10.1021/acs.joc.2c02491)
Supplement: Supplementary file 2 — jo2c02491_si_002.zip [file jo2c02491_si_002.zip › FID for publication/Information.docx]

Name of the manufacturer of the spectrometer used to collect the data:

^1^H NMR and ^13^C NMR spectra were recorded on Bruker Avance III HD 600, Bruker Avance-400, Bruker Avance-300 spectrometer in CDCl_3_

Acquisition software: Bruker TopSpin 3.5pl 7 on 600 MHz, Bruker TopSpin 1.3 on NMR 400 MHz, Bruker TopSpin 2.1 on NMR 300 MHz.

Processing programs used to analyze the data: Bruker TopSpin 2.1 and MestReNova

The field strength used to measure each nucleus:

**3a**: **^1^H NMR** 600 MHz; **^13^C NMR** 150 MHz

**3b**: **^1^H NMR** 600 MHz; **^13^C NMR** 150 MHz

**3c**: **^1^H NMR** 600 MHz; **^13^C NMR** 150 MHz

**3d**: **^1^H NMR** 600 MHz; **^13^C NMR** 150 MHz

**3e**: **^1^H NMR** 600 MHz; **^13^C NMR** 150 MHz

**3f**: **^1^H NMR** 600 MHz; **^13^C NMR** 150 MHz

**3g**: **^1^H NMR** 600 MHz; **^13^C NMR** 150 MHz

**3h**: **^1^H NMR** 600 MHz; **^13^C NMR** 150 MHz

**3i**: **^1^H NMR** 600 MHz; **^13^C NMR** 150 MHz

**3j**: **^1^H NMR** 300 MHz; **^13^C NMR** 75 MHz

**4a**: **^1^H NMR** 600 MHz; **^13^C NMR** 150 MHz

**4b**: **^1^H NMR** 400 MHz; **^13^C NMR** 100 MHz

**4c**: **^1^H NMR** 600 MHz; **^13^C NMR** 150 MHz

**4d**: **^1^H NMR** 600 MHz; **^13^C NMR** 150 MHz

**4e**: **^1^H NMR** 600 MHz; **^13^C NMR** 150 MHz

**4f**: **^1^H NMR** 600 MHz; **^13^C NMR** 150 MHz

**4g**: **^1^H NMR** 600 MHz; **^13^C NMR** 150 MHz

**4h**: **^1^H NMR** 300 MHz; **^13^C NMR** 75 MHz

**7**: **^1^H NMR** 600 MHz; **^13^C NMR** 150 MHz
